# Standardized Pre-clinical Surgical Animal Model Protocol to Investigate the Cellular and Molecular Mechanisms of Ischemic Flap Healing

**DOI:** 10.1186/s12575-023-00227-w

**Published:** 2024-01-17

**Authors:** Edita Aksamitiene, Ryan N. Heffelfinger, Jan B. Hoek, Edmund deAzevedo Pribitkin

**Affiliations:** 1 Department of Otolaryngology – Head and Neck Surgery, Thomas Jefferson University, 925 Chestnut St., 6Th floor, Philadelphia, PA 19107 USA; 2 Present address: Beckman Institute for Advanced Science and Technology, University of Illinois Urbana-Champaign, 405 N. Mathews Ave | M/C 251, Room 4357, Urbana, IL 61801 USA; 3 Department of Pathology, Anatomy and Cell Biology, Thomas Jefferson University, 1020 Locust St, Room 527, Philadelphia, PA 19107 USA; 4 Sidney Kimmel Medical College, 31st Floor, 1101 Market Street, Philadelphia, PA 19107 USA

**Keywords:** Excisional wound healing, Bilateral flap, Pedicled flap, Fasciocutaneous flap, Superficial inferior epigastric vessels, Axial pattern flap survival, Primary ischemia, Reperfusion injury, Secondary ischemia, Autologous tissue transfer

## Abstract

**Background:**

Some of the most complex surgical interventions to treat trauma and cancer include the use of locoregional pedicled and free autologous tissue transfer flaps. While the techniques used for these reconstructive surgery procedures have improved over time, flap complications and even failure remain a significant clinical challenge. Animal models are useful in studying the pathophysiology of ischemic flaps, but when repeatability is a primary focus of a study, conventional in-vivo designs, where one randomized subset of animals serves as a treatment group while a second subset serves as a control, are at a disadvantage instigated by greater subject-to-subject variability. Our goal was to provide a step-by-step methodological protocol for creating an alternative standardized, more economical, and transferable pre-clinical animal research model of excisional full-thickness wound healing following a simulated autologous tissue transfer which includes the primary ischemia, reperfusion, and secondary ischemia events with the latter mimicking flap salvage procedure.

**Results:**

Unlike in the most frequently used classical unilateral McFarlane’s caudally based dorsal random pattern skin flap model, in the herein described bilateral epigastric fasciocutaneous advancement flap (BEFAF) model, one flap heals under normal and a contralateral flap—under perturbed conditions or both flaps heal under conditions that vary by one within-subjects factor. We discuss the advantages and limitations of the proposed experimental approach and, as a part of model validation, provide the examples of its use in laboratory rat (*Rattus norvegicus*) axial pattern flap healing studies.

**Conclusions:**

This technically challenging but feasible reconstructive surgery model eliminates inter-subject variability, while concomitantly minimizing the number of animals needed to achieve adequate statistical power. BEFAFs may be used to investigate the spatiotemporal cellular and molecular responses to complex tissue injury, interventions simulating clinically relevant flap complications (e.g., vascular thrombosis) as well as prophylactic, therapeutic or surgical treatment (e.g., flap delay) strategies in the presence or absence of confounding risk factors (e.g., substance abuse, irradiation, diabetes) or favorable wound-healing promoting activities (e.g., exercise). Detailed visual instructions in BEFAF protocol may serve as an aid for teaching medical or academic researchers basic vascular microsurgery techniques that focus on precision, tremor management and magnification.

**Graphical Abstract:**

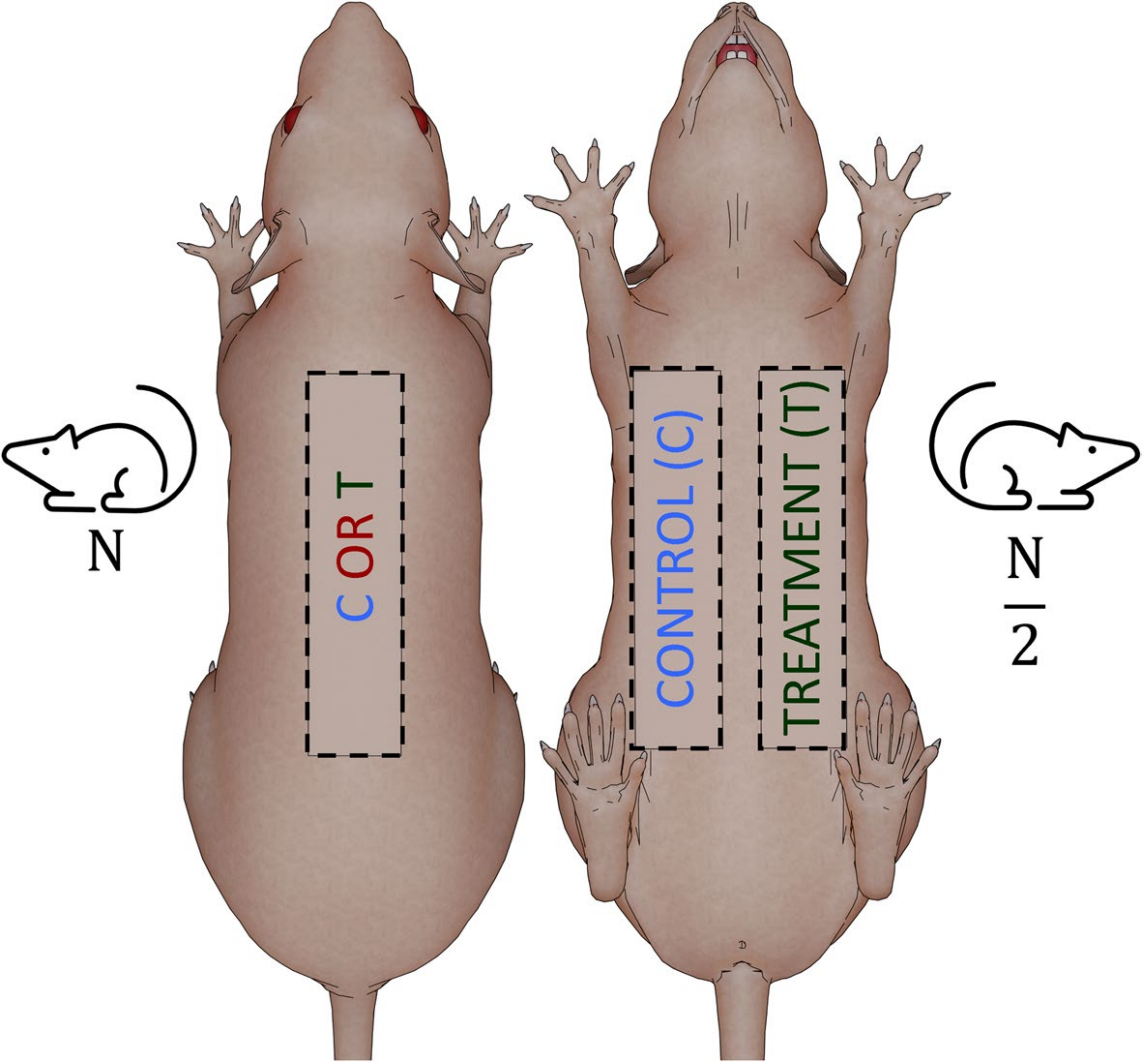

**Supplementary Information:**

The online version contains supplementary material available at 10.1186/s12575-023-00227-w.

## Background

**Fig. 1:**
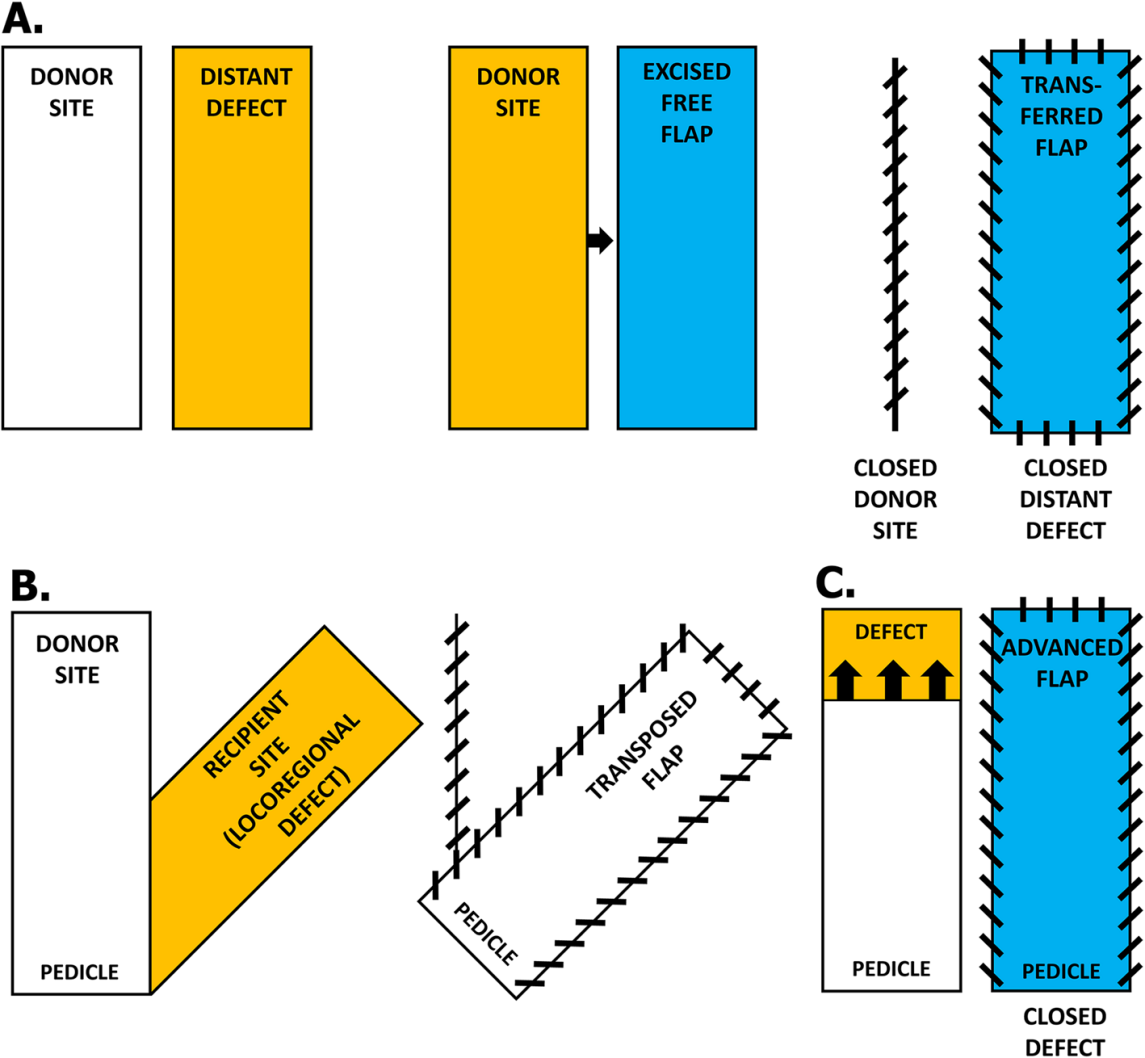
Example of surgical flap classification based on the method of tissue transfer onto the soft tissue defect (wound). Principles of creating the microvascular free flap (**A**), single-pedicled transposition flap (**B**) or single-pedicled advancement flap (**C**) during reconstructive surgery. Diagonal lines represent running sutures

Many trauma, oncological malignant or benign tumor resection and other developed soft tissue defect (e,g,, arterial or venous ulcers, pressure sores, hernia, necrolytic skin infections) cases require immediate or planned surgical intervention using flaps. Flaps are classified based on their [a] tissue composition (e.g., cutaneous, fasciocutaneous, myocutaneous, osteomusculocutaneous), [b] location (e.g., local, regional, distant); [c] arrangement of blood supply; [d] configuration and [e] the method of transfer onto the wound [1, 2]. For instance, a microvascular free flap is elevated from an uninjured donor site, fully detached, and transferred to the remote recipient site (Fig. 1A). Such flap is exposed to varying times of *primary ischemia* (PI) before being reconnected to recipient vessels (anastomosis). By contrast, a local or regional pedicled flap remains attached at the harvest site by vascular subcutaneous tissue base known as a *pedicle*. During surgery, such a pedicled flap can be rotated a certain number of degrees around a pivotal point located at the base of the flap to reach and cover an adjacent, nearby, or distant recipient area (rotational, transposition or interpolated flaps) (Fig. 1B). In other cases, a pedicled flap is simply moved over a local or distant defect without altering the plane of the pedicle (advancement flaps) (Fig. 1C) [3, 4].

Preclinical wound healing models distinguish among incisional and excisional wounds, flaps, and skin grafts. Unlike small incisional and excisional wounds, flaps do not heal by contraction. A graft has no intact blood supply and therefore relies entirely on diffusion of nutrients and a number of new blood vessels derived from the recipient wound bed. By contrast, a flap contains native blood supply. In an *axial pattern flap* (APF) the blood is delivered via a dominant and well-defined cutaneous artery that is often accompanied by a large-scale vein. In a *random pattern flap* (RPF), the blood is supplied via an unnamed subdermal perforator plexus. Due to their increased length and more robust inherent blood supply, APFs are ideally suited for reconstruction of large soft tissue defects that are beyond the reach of shorter and less predictable RPFs. Nevertheless, the survival pattern of APFs is typically based on the integrity and extent of their main feeding and draining vessel(s). Thus, a distal portion (the very tip) of the APF that exceeds the distance of the underlying longitudinal artery may behave as a graft or an RPF: after a reconstructive surgery, these distal tissues stay deprived of oxygen (ischemic) significantly longer depending on the rate of reperfusion, angiosome formations and extent of neovascularization, which appears later in wound healing process, but in most animal models – typically within a week [5–7]. To a certain degree, naturally occurring hypoxia (the lack of oxygen) will stimulate the APF-infiltrated and APF-residing cells to release pro-inflammatory cytokines and angiogenic factors contributing to the inosculation (connection of pre-existing vessels to the vessels of recipient wound bed and sidewalls) and angiogenesis (the ingrowth of new vessels). But if hypoxia persists, sustained inflammation combined with local accumulation of reactive oxygen species and lactic acid buildup will lower the intracellular pH and cause severe DNA damage. As a result of extensive apoptosis-related protein activation, depleted bioenergetic resources such as nicotinamide adenine dinucleotide (NAD +) would trigger massive cell death and, subsequently, devitalized tissue breakdown (necrosis) [8–10].

Despite recent advances in surgical techniques and post-operative monitoring that contribute to a high (~ 85–95%) flap success rate [11–14], complications associated with vascular compromise and ischemic partial- or full-thickness necrosis remain a significant clinical problem, particularly in angiopathic, obese, post-irradiated, actively smoking and/or alcohol-abusing individuals [15–24]. In addition to inducing debilitating patient stress, flap failures inordinately contribute to health care costs due to prolonged hospitalization, readmission and revision surgery [25]. The success rate of salvaged microvascular free flaps ranges from 28% to over 90%, depending on the type of the flap being used, the etiology of its failure, the timing of its salvage and the overall experience of medical center surgeons and staff [26–28].

*Experimental translational plastic and reconstructive surgery research animal models of complex full-thickness wound closure and healing using RPFs or AFPs* are indispensable tools in studying physiological and pathophysiological responses to acute intermittent hypoxia and ischemic tissue injury [29]. These models provide insights into a spatiotemporally regulated activation machinery of integrated signaling pathways that induce transcription of genes controlling the survival, motility, proliferation, and differentiation of multiple functionally distinct cell populations. Under the influence of diverse endogenous stimuli these locally residing, or newly arrived wound-bed cells are capable of either maintaining or ceasing inflammation. Some of them promote vascularization through angiogenesis and lymphangiogenesis, change hemodynamics and induce tissue remodeling. A complete understanding of each physical and biological facet of the reparative ladder resulting in a fully recovered flap and completely closed wound is essential to predicting the clinical outcomes of the reconstructive surgery.

Many preclinical research animal wound healing models are primarily designed to investigate tissue ability to progress through different phases/stages of wound healing under unperturbed or minimally perturbed (control/sham) and experimental “treatment” conditions. The latter includes irradiation [30], photodynamic therapy [31], treatment with pharmacological agents [32–34], recombinant peptides [35], plasmid DNA or siRNA compounds [36], synthetic or natural products [37, 38]) etc. Sometimes the investigators aim to mimic severe medical complications (e.g., vascular thrombosis, edema, ulcerative and infected wounds) [39] or reproduce well-known comorbidities (e.g., obesity, diabetes, ageing, smoking, alcohol intake etc.) [40–42] or stimulatory responses (e.g. exercise) [43]. Different routes and/or regimen of single or combined drug administration [44, 45], ischemic preconditioning [46, 47], delaying [48] and numerous perioperative flap treatment or handling strategies [49–53] have been evaluated for their efficacy at reducing the risk of compound flap loss. However, in all *conventional* experimental studies one randomized animal group typically serves as a control, while another group undergoes the desired treatment(s). The results obtained from such studies suffer from reduced statistical power due to high subject-to-subject variability. Pre-surgical fluctuations in baseline levels of activated proteins/genes and circulating factors may mask the moderate and less robust effects of chosen treatment(s), especially if it is applied directly onto the flap. Thus, to achieve stronger statistical power, a larger number of animals must be used.

Here we present a detailed experimental protocol to develop a standardized, versatile, scalable, transferable, and reproducible full-thickness wound closure and healing model, which simulates an actual clinical procedure of autologous free tissue transfer and is based on a *bilateral* *flap*. In this modified surgical pre-clinical animal model, a treated or perturbed ventral flap is paired with a contralateral control ventral flap on the same animal which heals under similar physiological or pathological conditions. Significantly greater control for between-animal variability is obtained while sacrificing fewer animals. The presented method is a *fusion* of three clinically relevant experimental techniques: pedicled APF advancement, free microvascular flap surgery and flap salvage. We provide step-by-step guided visual instructions on creating the *bilateral epigastric fasciocutaneous advancement flap* (BEFAF) model for the intermittent collection of tissue biopsies and a final evaluation of ischemically-challenged flap survival at one designated endpoint in laboratory rat (*Rattus norvegicus*), because training with live rats is considered the gold standard in the current microsurgical training [54]. The chosen rats may be any desired strain, sex, age, and weight – due to the differences in skin anatomy and ischemic tolerance (female vs male), or wound healing efficacy (young vs old)—they all come with different advantages. These animals may be housed under routine or specialized husbandry conditions and fed ad libitum or a specialized (e.g., alcohol-containing) diet. They may be randomly selected and divided into two or more experimental groups receiving any control or experimental treatment prior to flap raising surgery. We overview data collection and analysis strategies showing representative micrographs of flap tissue sections obtained from male or female animals subjected to identical or similar surgical procedures. Finally, we discuss potential model applications, limitations, and anticipated challenges.

## Materials and Methods

### Design of BEFAF Model

In the current adaptation of the APF model to reconstruct an abdominal wound, we employ a bilateral epigastric fasciocutaneous advancement flap (BEFAF), which is based on two ventral APFs supplied by the caudal superficial inferior epigastric artery (SIEA) and drained by superficial inferior epigastric vein (SIEV). SIEA and SIEV branch from the femoral artery/vein in the left and right groin regions. In clinical practice, SIEA-based epigastric flaps are used either as unipedicled or bipedicled advancement or transposition flaps to cover ipsilateral or contralateral wounds on the abdomen, perineum or hindlimbs, or as free flaps to repair facial or distant breast defects as a result of trauma, burns, tumor resection or infection [55–63].

 First, we create a full-thickness soft tissue defect in the upper abdomen region: a piece of fasciocutaneous tissue measuring between 0.5 × 3 cm and 1 × 3 cm is excised from the top portion of a flap prior to its elevation. Due to the absence of extensive physical manipulation, the excised tissue thus reflects the basal activation, expression and/or spatial distribution patterns of genes, microRNAs, and proteins. In routine histological tissue sample analysis, it represents morphologically normal unwounded skin (control). The qualitative features as well as quantitative measures obtained at this baseline (0 h) can be readily compared to the ones obtained within the proximal (P), middle (M) and distal (D) flap segments at specific time points following an excisional and ischemic injury. Taking a second biopsy from the D segment of the raised flap prior to its advancement and final wound closure results in a *fully advanced* APF with a clinically recommended 3:1 or 2.5:1 height to width ratio, although these guidelines are not absolute. If the combined excised tissue area is insufficient for analysis (less than 1.5 × 3 cm), then a third biopsy may be acquired at any intermediate time point via minor survival surgery. The degree of tissue necrosis and overall survival, expressed as flap viability percentage (FV%), is evaluated by *digital planimetric analysis* using digital photographs of ready-to-be harvested flaps at any designated postoperative day (POD) [51]. These endpoints typically represent the peaks of major wound healing stages: inflammation (POD1 to POD3), proliferation (POD 5 to POD 7) and short-term or long-term remodeling (POD 9 to 14 and beyond). The *rate of flap healing/failure* may be calculated through this sequential planimetric analysis. The most common variables for comparative BEFAF analysis are listed in Table 1.

**Table 1:** Suggested pre-operative or perioperative flap handling or treatment conditions for the comparative short-, intermediate- or long-term whole flap or segmental gene or protein expression, cell signaling, extracellular vesicle release, neovascularization, tissue survival and other analyses within one subject

Left flap	Right flap
Control naïve flap (raised and placed down)	Ischemic flap (e.g., 2 h of PI)
Control naïve flap (raised and placed down)	Flap with inserted sterile silicone sheet
Control naïve flap (raised and placed down) APF	Permanently ligated artery, vein, or both^a^ RPF
Global PI (e.g., 2 h)	Global PI (e.g., 6 h)
Global PI (e.g., 2 h) REP_1_ (e.g., 4 h) Global SI (e.g., 2 h)	Global PI (e.g., 2 h) REP_1_ (e.g., 12 h) Global SI (e.g., 2 h)
Global PI (e.g., 4 h) REP_1_ (e.g., 12 h) Global SI by AVO (e.g., 2 h)	Global PI (e.g., 4 h) REP_1_ (e.g., 12 h) Global SI by AVO (e.g., 6 h)
Global PI (e.g., 2 h) REP_1_ (e.g., 6 h) SI by AO (e.g., 2 h)	Global PI (e.g., 2 h) REP_1_ (e.g., 6 h) SI by VO (e.g., 2 h)
Global PI (e.g., 2 h) REP_1_ (e.g., 6 h) SI by AO (e.g., 2 h)	Global PI (e.g., 2 h) REP_1_ (e.g., 6 h) SI by AO (e.g., 6 h)
Global PI (e.g., 2 h) REP_1_ (e.g., 6 h) SI by VO (e.g., 1 h)	Global PI (e.g., 2 h) REP_1_ (e.g., 6 h) SI by VO (e.g., 3 h)
Ischemic preconditioning (e.g., 10 min × 3 times)	No ischemic preconditioning Control naïve flap (raised and placed down)
Delayed flap (e.g., 7 days)	No delay
Implantation of control PVA sponges	Implantation of drug-preloaded PVA sponges
Saline injection	Drug injection
Intradermal delivery of non-targeting control siRNA, shRNA, or antisense oligonucleotide	Intradermal delivery of targeting siRNA, shRNA, or antisense oligonucleotide
Topical treatment with control vehicle	Topical treatment with an agent of interest
No anastomosis applied	Anastomosis

*Abbreviations: PI* Primary ischemia, *SI* Secondary ischemia, *AO* Arterial occlusion, *VO* Venous occlusion; *AVO* Arteriovenous occlusion, *REP*_*1*_ Primary reperfusion

^a^flap ligation is a positive control used to estimate the maximal extent of flap failure upon complete blockade of blood flow through the main artery. This procedure turns the APF into RPF

Importantly, with this BEFAF model, one may fully recreate *microvascular free flap surgery*. One or both flaps can be entirely detached from their primary blood supply followed by end-to-patch anastomosis. Alternatively, atraumatic vascular clamps may be gently applied onto both feeding and outflow vessels in one or both flaps for as long as it would presumably take the surgeon to separate the tissue, transfer and reconnect it to the exposed vessels of the wounded area. The global *primary ischemia* (PI) typically lasts no more than 2 h, but depending on the experimental aims, flap type and animal species used, the induced PI time may be tailored to be either shorter or longer. Furthermore, the BEFAF model presents an opportunity to examine the effects of acute or late *secondary ischemia* (SI). SI may be artificially induced by temporary closure of the SIEA, SIEV or both vessels after a certain *primary* *reperfusion* (REP_1_) period following global PI. Such experimental design directly and selectively mimics *arterial* (AO), *venous* (VO) or *mixed* *arterio-venous* (AVO) *occlusion*. These types of vascular complications most frequently occur within first 72 h after free tissue transfer and are a major contributor to flap loss [64–67]. In this BEFAF model, flap relief from the SI would simulate a clinical *flap re-exploration and salvage* procedure. Once the congestion is alleviated, the flap would go through the *secondary reperfusion* (REP_2_) and recovery period.

Both REP_1_ and REP_2_ are else known as reoxygenation events during which blood supply returns to the ischemic regions of the tissue. Depending on the degree of flap hypoxia and the rate of blood return, the restoration of circulation may result in mild or severe inflammation and oxidative damage through the induction of oxidative stress rather than (or along with) restoration of normal tissue function. This phenomenon of *ischemia–reperfusion* *injury* (IRI) is well-known and has been widely explored mechanistically [68, 69]. The BEFAF model allows investigators to assess the tolerance of male vs female or treated vs untreated animal flaps to the selective PI and/or SI events, generating probit analysis curves and calculating the *critical ischemic time* (CIT_50_) values when 50% of flaps become totally necrotic (Table 2) [32]. In the BEFAF surgery workflow provided below, the left-side flap is subjected to 4 h of global PI, 2 h of REP_1_ and 4 h SI by VO, while right-side flap is subjected to 4 h of global PI, 2 h of REP_1_ and 4 h of SI by AO.

**Table 2:** Experimental research animal model designs for estimating the critical primary and secondary ischemia times for single-pedicled flaps in male and female subjects at 95% power and planned minimum 3 h time gap between two anesthesia events on the same subject

Animal number per group excluding attrition		Left flap	Right flap
**Study Design A**			
**Group A**	**Group B**		
7 female	7 male	No PI	Ligation^a^
7 female	7 male	PI for 2 h	PI for 8 h
7 female	7 male	PI for 4 h	PI for 10 h
7 female	7 male	PI for 6 h	PI for 12 h
*N* = 28	*N* = 28	*n* = 7, *r* = 1 per group	*n* = 7, *r* = 1 per group
**Study Design B**			
7 female	7 male	No PI	PI for 2 h
7 female	7 male	PI for 2 h, REP for 6 h, SI for 1 h	PI for 2 h, REP for 6 h, SI for 4 h
7 female	7 male	PI for 2 h, REP for 6 h, SI for 2 h	PI for 2 h, REP for 6 h, SI for 6 h
*N* = 21	*N* = 21	*n* = 7, *r* = 1 per group	*n* = 7, *r* = 1 per group
**Study Design C**			
7 female	7 male	No PI	PI for 2 h
7 female	7 male	PI for 2 h, REP for 12 h, SI for 1 h	PI for 2 h, REP for 12 h, SI for 5 h
7 female	7 male	PI for 2 h, REP for 12 h, SI for 3 h	PI for 2 h, REP for 12 h, SI for 7 h
*N* = 21	*N* = 21	*n* = 7, *r* = 1 per group	*n* = 7, *r* = 1 per group
**Study Design D**			
7 female	7 male	No PI	PI for 2 h
7 female	7 male	PI for 2 h, REP for 24 h, SI for 2 h	PI for 2 h, REP for 24 h, SI for 8 h
7 female	7 male	PI for 2 h, REP for 24 h, SI for 4 h	PI for 2 h, REP for 24 h, SI for 10 h
7 female	7 male	PI for 2 h, REP for 24 h, SI for 6 h	PI for 2 h, REP for 24 h, SI for 12 h
*N* = 28	*N* = 28	*n* = 7, *r* = 1 per group	*n* = 7, *r* = 1 per group

^a^vessel ligation is a positive control used to estimate the maximal extent of flap failure upon complete blockade of blood flow through the main artery

One additional advantage of using the BEFAF model is that during an inflammatory phase of wound healing such flap produces copious amounts of *wound exudate* (WE). This growth factor-, cytokine-, chemokine-, metalloproteinase- and extracellular vesicle (EV)-enriched fluid freely travels from the caudal to the cranial regions of ventral flaps facilitating the diffusion of vital healing factors that in turn promote cell migration across the wound bed, cell proliferation, metabolism and aid the autolysis of damaged tissues [70]. The flap can be pre-opened for the manual collection of WE into a sterile 1000 μL pipette tip after a flap is gently squeezed at the base. But more conveniently, one or more sterile polyvinyl alcohol (PVA) sponges can be implanted underneath the flap to absorb the WE over time [71]. Control sponges may be subcutaneously placed in the midline between the left and right flap or outside of each flap further away from the wound. The sponges are eventually removed by opening the pockets in the respective portion(s) of the flap(s) through minor survival and/or non-survival (endpoint) surgery. Alternatively, the PVA implants may be preloaded or continuously injected with a control vehicle or an experimental treatment solution to evaluate its impact on the migratory properties of different wound-bed cell populations, rate of granulation tissue (GT) formation and its health (thickness, vascularity), lymphangiogensis as well as deposition of extracellular matrix and collagen fibers [72–75].

The materials required for an optional WE extraction from implanted PVA sponges and an optional isolation of tissue-derived proteins, nucleic acids (mRNA), or EVs are provided in the Additional File 2.

### Animals

Male and female 10–18-month-old retired-breeder Sprague–Dawley (SD) rats (Charles River Laboratories, Malvern, PA and Envigo, Indianapolis, IN) with an average weight of 310 g and 570 g or male 10-month-old retired-breeder Lewis rats with an average weight of 542 g (Envigo, Indianapolis, IN) were used in this study. Rats of same sex were acclimated at least 7 days prior to surgery under standard laboratory conditions (temperature 21 °C ± 2 °C, humidity 55% ± 10%, 12:12 h light–dark-cycle). Due to the weight (> 500 g) and retired breeder status males were housed singly. Females were housed in tandem. Food and water were provided in a standard manner. Some animals received liquid ethanol-supplemented diet. We show representative photos of experimental male or female animals.

### General Laboratory Equipment and Materials for the Surgeon

This procedure used standard ACS ≥ 95% purity or molecular biology grade laboratory reagents/chemicals as well as standard veterinary surgery and microsurgery equipment/tools. In some instances, the shortened links to the randomly selected vendors that can supply specific surgical instruments or reagents used in this study are provided next to the item.

1. Personal protective equipment (PPE): surgical gown, ear loop face mask, hair cap, shoe covers, sterile surgical gloves, non-sterile gloves, safety glasses.
2. Stainless steel instrument tray with flap cover – for autoclaving and storage of surgical instruments
3. Stainless steel Grafco Flat Type instrument tray – for placing the surgical items during the procedure (Additional File 2, Fig. S1)
4. Sharps disposal container – for disposal of needles and syringes
5. Biohazard disposal container and biohazard bags – for safe disposal of biohazardous trash, including pipette tips.
6. Countdown timers
7. Digital camera with macro-view function

Optional:

8. Magnifying spectacles or loupes (2.5–3 × , depending on the eyesight of the surgeon) with/without illumination or separate LED headlamp (> 500 lumens).

### Materials for Animal Preparation for the Surgery and Flap Design

1. Single or dual-channel anesthesia system with gas vaporizer, inlet flowmeter, charcoal filters, high pressure oxygen hose, oxygen regulator, and low-profile anesthesia masks adapted for rodents.
2. Small-animal scales
3. Secure animal transportation cart
4. Small animal heating pad
5. Handheld hair clipper and/or trimmer (e.g., WAHL for Home Pet).
6. Ophthalmic veterinary ointment (e.g., Puralube).
7. Betadine (10% povidone iodine) antiseptic solution.
8. Isoflurane inhalant anesthetic for veterinary use (USP)
9. Sterile non-woven or gauze sponges (e.g., Dermacea 4″ × 4″, 6-Ply by Covidien, #441405)
10. Disposable surgical under pads:
  X-large 30″ × 36″ Wings Ultra by Covidien.
  Small 17″ × 24″ or medium 23″ × 24″ Dri-Sorb by Attends.
11. C-fold paper towels (e.g., Kleenex)
12. Sanitizing 70% ethanol spray
13. Sterile disposable skin marking pen with ruler (Viscot, #1422SRL9-100)
14. 1:3 or 1:2.5 ratio flap design template made from sturdy card-paper with extra 1–2 cm in length if distal biopsy collection is intended.

### Materials for Major or Minor Survival Surgery

1. Pre-operative analgesic (opioids such as sustained release buprenorphine hydrochloride are typically obtained from LAS veterinarian staff). Otherwise, take a new sterile 1 ml syringe, slowly draw in analgetic solution into the syringe. Point the syringe upward to bring any air bubbles to the top of the needle. Plunge the syringe slowly to expel any air until only liquid expels from the syringe. Double check volume, place the needle cap and set a syringe aside.
2. Sterile Dulbecco's Phosphate-Buffered Saline (DPBS) (Cytiva, #SH30028.02) or HyClone™ Hank's Balanced Salt Solution (HBSS) without calcium, magnesium, and phenol red (Cytiva, #SH30588.01) – for irrigation of raised flaps while maintaining cell tonicity and viability.
3. 1% Lidocaine HCl—for topical irrigation of pedicle to prevent or reduce traumatic vasospasm(s). Prepare 1% solution using sterile DPBS.
4. 10 mL saline pre-filled syringes (e.g., BD PosiFlush™ by BD Biosciences).
5. 1 mL sterile disposable syringes with Luer-Lok tips
6. Sterile 21 gauze hypodermic needles
7. Hot glass bead dry sterilizer (e.g., Germinator 500 by BrainTree Scientific) – for surgical instrument sterilization in between two successive animals that undergo flap raising surgery on the same day.
8. Multi-Enzymatic Cleanser – for surgical instrument cleaning (McKesson, #484,478)
9. Sterile surgical instruments (available from Electron Microscopy Sciences (EMS; Hatfield, PA), Kent Scientific Corporation (KSC; Torrington, CT), Fine Science Tools (FST, Foster City, CA), World Precision Instruments (WPI; Sarasota, FL) or other commercial sources):
  Iris Supercut scissors (curved or straight)—for tissue cutting.
    Example: https://tinyurl.com/5n7r8mez
  Metzenbaum-Nelson scissors (straight) – for excisional biopsy cutting.
    Example: https://tinyurl.com/3jmc5r8d
  Mayo dissecting scissors (curved with beveled blades, medium or large size) – for tissue dissection.
    Example: https://tinyurl.com/3p3nnb68
  McPherson Vannas or Noyes Iris micro scissors (straight with beveled smooth blunt/blunt or sharp/sharp blades) – for dissection of tissue around pedicle and vessel separation.
    Example: https://tinyurl.com/2p8mfwc9
  Halsey micro needle driver/holder with serrated tips – for holding a needle during vessel separation.
    Example: https://tinyurl.com/mvm7nepx
  Kelly Hemostat forceps (straight with serrated tips) – for suture needle grasping and holding.
    Example: https://tinyurl.com/3trzus3n
  Adson tissue forceps with 1 × 2 teeth – for lifting tissue during suturing.
    Example: https://tinyurl.com/6kbsap4m
  Micro/fine forceps (slightly and fully curved, smooth jaws) – for lifting the pedicle and fascia. 
    Example: https://tinyurl.com/yckfr564
  Harms micro suturing (straight) and Mcpherson tying forceps (angled) – for performing anastomosis (optional)
    Example: https://tinyurl.com/422r2252 and https://tinyurl.com/bddtpus3
  Flat-tip tweezers with upward bent tips (EMS #78336-36A) – for handling PVA sponges
    Example: https://tinyurl.com/33rbmyfv

10. Atraumatic vascular clamps:
  7 mm B-1 V, 3.5 × 1 mm (FST, #00396–01) – for vein occlusion
  11 mm B-2 V, 5.5 × 1.5 mm ((FST, #00398–02) – for artery occlusion and global ischemia
  16 mm – B-3 V, 7.5 × 1.75 mm (FST, #00400–03) for occlusion of larger diameter vessels in larger laboratory animals.
    Example: https://tinyurl.com/yc22wa4m
11. Clamp applying forceps with lock (FST, #00071–14) – for handling vascular clamps.
12. Disposable or reusable battery-operated high temperature cautery with fine tip (e.g., Bovie Medical #AA01; EMS #72,976–31) – to prevent bleeding during microvascular tissue dissection.
13. Sterile 4–0 size sutures with 16 mm (for female rats) or 19 mm (for male rats) 3/8 circular reverse cutting needle:
  Non-absorbable 18″ or 30″ POLYPROPYLENE or NYLON – to place running sutures.
  Non-absorbable 18″ or 30″ SILK – to place temporary sutures and vascular separation.
  Absorbable 18″ or 30″ VICRYL Polyglactin 910 – to place buried simple-interrupted sutures.
14. Elizabethan collars (small, medium, large, or x-large).
15. Small or medium size LIGACLIP® Multiple-Clip Applier (MCA) – for permanent occlusion of vessels (e.g., Ethicon, #MCS20 or #MCM20).

Optional:

16. 10 mL BD PosiFlush™ Pre-Filled Heparin Lock Flush Syringes (100U/mL) – for topical dropwise irrigation of vascular lumen prior to vascular occlusion (BD Biosciences, #306512).

### Materials for Tissue Biopsy and Flap Excision

1. Liquid nitrogen (LN_2_) supply and portable LN_2_ dewar (e.g., 4L by Nalgene)
2. Cryogenic flat round-tip tweezers
3. 18 mL high-density polyethylene (HDPE) scintillation vials with threaded cap (e.g., Fisher Scientific, # 14–955-393) – for tissue sample storage and easy access
4. Fine-tip histology marker and LN_2_-proof cryo-marker (e.g., Dual-point UltraCruz, Santa Cruz Biotechnologies,# sc-360977)
-80°C freezer – for long-term tissue and sample storage.
6. Animal euthanasia cart with CO_2_ gas tank supply
7. Small biohazardous waste bags or specified animal carcass disposal bags

Optional: Sterile Disposable McKesson Biopsy Punches (available in 2—10 mm sizes).

### Tissue Preparation for Histology

1. Unisette™ 1-chamber histology embedding/processing cassettes with 1 mm openings (Ted Pella)—for smaller size and leaner specimens:
  Outside dimensions: 28.5 × 41 × 6.7 mm (1.12" × 1.61" × 0.26").
  Inside dimensions: 26 × 30 x 5 mm (1.02" × 1.18" × 0.2").
2. Macrosette™ 1-chamber histology embedding/processing cassettes with 1 mm openings (Ted Pella)—for thicker specimens
3. Micromesh™ 4-compartment biopsy cassettes – for excised pedicles
4. Cassette marking histology pen (e.g., Statmark by StatLab)
5. 90 ml containers with prefilled 10% Neutral Phosphate-Buffered Formalin (pH 7.2 ± 0.5; vials) (e.g., StatLab, # NB0345)
6. 1X PBS buffer without magnesium and calcium—for cassette washing after fixation. Store at RT.
7. 70% Ethanol solution – for cassette storage until analysis at + 4°C.
8. Large glass staining dishes.
9. Stains of interest, e.g., Masson’s Trichrome (MT) and Hematoxylin & Eosin (H&E).
10. Classical histology lab equipment if not using Core Histology facility.

### Experimental Animal Preparation for Survival Surgery

To reduce the time of animal exposure to anesthesia during the major survival surgery, and to minimize the risk of overlapping PI or SI time between the sequentially operated animals, the animals are shaved 16—24 h prior to flap raising procedure:

1. The heating pad is set for 122°F (50 °C) and covered with two surgical underpads.
2. The anesthesia machines is turned on to prefill the padded rodent sedation chamber with 4–5% induction isoflurane gas.
3. The animal is placed in the chamber and kept there for up to 4 min.
4. 3% isoflurane flow is then redirected into the nose cone attached to the anesthesia machine.
5. The sedated rat is removed from the chamber and placed onto the heating pad in a prone position.
6. The lubricate ointment is generously applied onto animal’s eyes.
7. The rat is placed back into a supine position and its fur is shaved until the nude skin is exposed.
8. The top surgical pad containing shaved fur is removed.
9. Shaved skin areas are wiped with betadine solution, which is copiously applied onto a non-woven sponge.
10. Animal weight is recorded in the post-operative animal monitoring sheet (POAMS) (see Additional File 1).
11. The animal is returned to the cage and monitored until awakened.

NOTE: If less than three consecutive animal surgeries are anticipated one after the other within the same day, animal shaving should be performed on the same day as major survival surgery without an animal being awakened. With experience and proper equipment, animal fur clipping takes less than 12 min. Chemical hair removal creams such as “Nair” are not recommended as they may affect both baseline and injury-inducible biological molecule distribution and/or expression patterns, cause skin irritation or allergic reaction.

### Major Survival Surgery

As a visual aid, we used black non-absorbable silk sutures in every but the final suturing step. These sutures must be replaced with non-absorbable sutures where and when indicated in the protocol.

#### Preparation

1. Appropriate PPE is donned by the surgeon.
2. The HDPE tubes, 10% formalin-prefilled containers and histology cassettes are labeled for appropriate specimen.
3. The vascular clamps, that are being stored in enzymatic solution, are removed, washed with distilled water, and then placed in an Eppendorf tube containing 70% alcohol.
4. The hot glass bead sterilizer is turned on to preheat.
5. The heating pad is set to reach 122°F (50 °C). It is covered with four surgical underpads: 1 extra-large and 3 medium or small.
6. If an optional PVA sponge implantation is desired, then two sterile 9 mm × 15 mm ear wicks are placed onto 100 × 20 mm tissue culture dish prefilled with sterile PBS. These wicks are then cut into three pieces per each using a disposable scalpel. The lid of tissue dish is closed to prevent the PVA sponges from drying.
7. The dose of analgesic agent is prepared for each individual animal based on the recorded animal weight (for example, our rats received 1.0–1.2 mg/kg of Buprenorphine-HCl-SR formulation subcutaneously (sc)).
8. Dewar flask is prefilled with liquid nitrogen (LN_2_).
9. Isoflurane and oxygen tank levels are checked, and the proper amount of isoflurane is added to the gas anesthesia machine chamber before the experiment if needed. The anesthesia machine is turned one to prefill the padded rat sedation chamber with 4–5% isoflurane gas.

#### Flap Design

To prevent tension-associated flap failure, the BEFAF model allows taking only a limited number of tissue biopsies from the distal clinically most relevant part of the flap prone to failure. Additional biopsies, if required, may be obtained from the regions that lie along and close to the suture line, however, such biopsies introduce an additional variable. Therefore, in flap survival studies, no interference other than collection of biopsies from the distal portion of the flap throughout the entire course of experiment is strongly recommended. For a flap with initial measurements of 9.5 × 2.5 cm or 9.5 × 3 cm, the maximal flap advancement is 2 cm, which leaves a healing area of 3:1 or 2.5:1 length to width ratio. Consequently, one can obtain two 1 cm or four 0.5 cm wide biopsies from each flap. The 1 × 3 cm tissue strip is big enough to be divided into three or four segments for postoperative imaging or biochemical analysis, especially if sample pooling is allowed. On the other hand, such long and wide initial flap can be created only in adult, over 500 g rats. These are usually males. Female rats rarely exceed 350 g of weight. Therefore, for smaller animals, flap dimensions should be downsized appropriately, keeping in mind that a healing flap shrinks approximately 0.5 – 1 cm, which may partially limit animal movements and cause distress.

1. The animal is placed into the anesthetic sedation chamber and kept there for up to 4 min.
2. 3% isoflurane flow is redirected into the nose cone attached to the anesthesia machine.
3. The sedated rat is removed from the chamber and placed onto the heating pad in a prone position.
4. Animal’s eyes are lubricated by applying the ointment, and analgesia is injected sc.
5. The animal is turned back to the supine position. The use of medical tape for animal stabilization during the flap design procedure is not recommended, as the animal skin should be in the most natural and relaxed condition.
6. Flap boundaries are identified by applying a pre-cut cardboard 9.5 × 3 cm flap design template and drawing a single dotted line with a skin marker. After removing the template, a second dotted line is drawn above the first just a few millimeters apart. Finally, a solid line is drawn through the inner dots as shown in Fig. 2A.

**Fig. 2:**
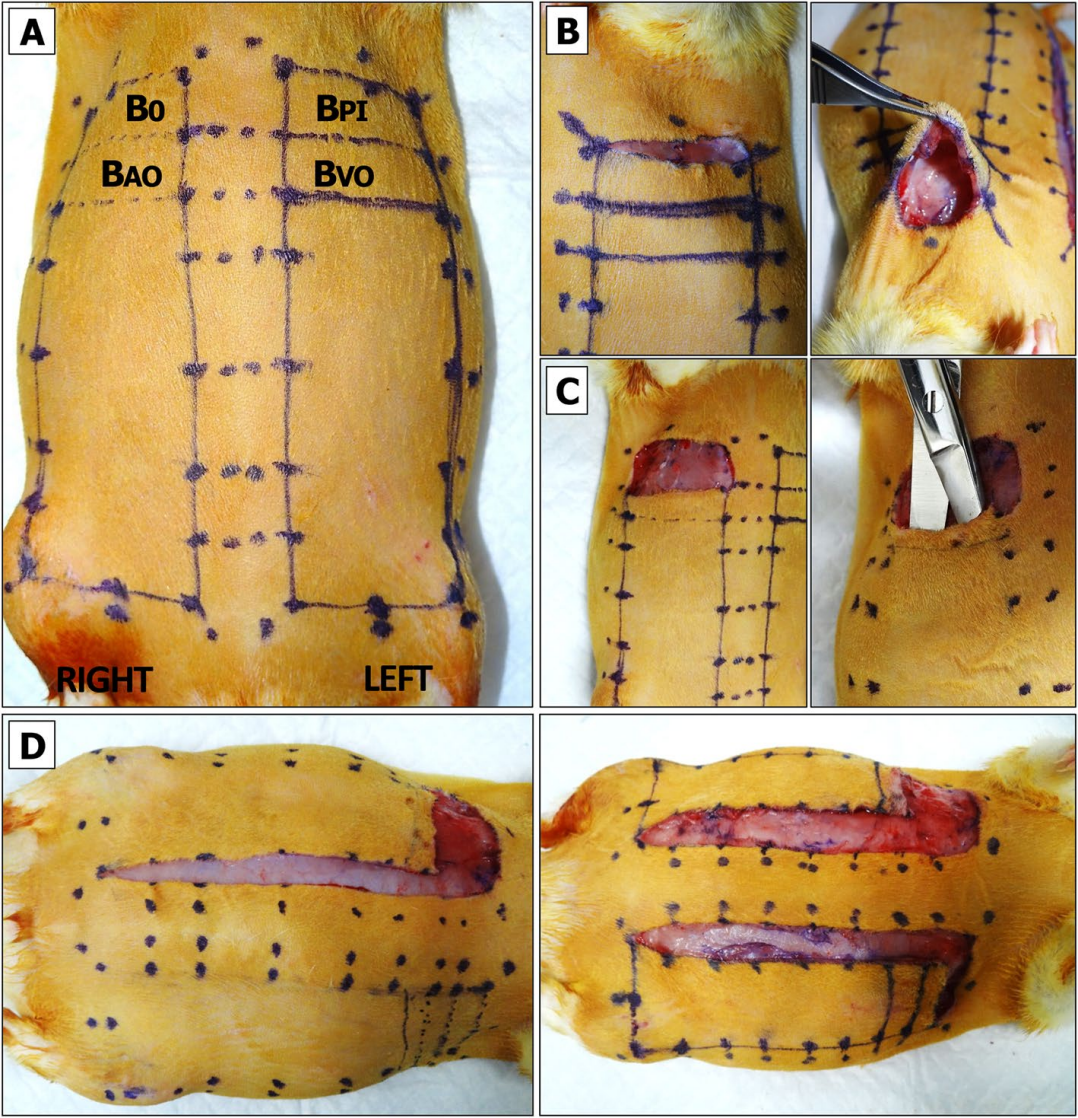
Design of BEFAF. **A** Contours for flap elevation and biopsies. **B** Preparation of flap for baseline biopsy (B_0_) excision. **C** Flap pocket after B_0_ excision is used for blinded dissection and partial separation of fasciocutaneous tissue from the muscle. **D** Superficial cutting of inner flap sidewalls
7. Flap areas that will be used for the excisional biopsies are then marked. In the example provided herein we defined four 1 × 3 cm biopsy areas:
  A) B_0_ – baseline.
  B) B_PI_ – to be excised after the end of global primary ischemia (PI) and prior to reperfusion.
  C) B_VO_ – to be excised after the end of secondary venous ischemia.
  D) B_AO_ – to be excised after the end of secondary arterial ischemia (Fig. 2A).

Assuming the morphology and cellular activities in left and right side are similar under identical conditions, we aim to take B_0_ and B_PI_ biopsy only from one of the flaps. NOTE: in an alternative experimental design, one can excise four 1 × 1.5 cm biopsies out of each flap that would include in a dedicated B_0,_ B_PI_ and B_VO_ or B_AO_ sample plus one extra sample, e.g., B_REP1_—to be taken after the end of primary reperfusion prior to SI. However, the created gap resulting from partial tissue excision would need to be protected by gauze during intermediate incubation periods prior to final wound closure (see Additional File 2, Fig. S2).

#### Flap Raising

The tissues being raised in this protocol consist of skin and fascia but exclude muscle. Depending on the research objective, other types of composite flaps (e.g. myocutaneous flap) could be raised instead, but they may require another suturing approach, and due to different sensitivity to ischemia – adjusted PI and SI times [76, 77].

**Fig. 3:**
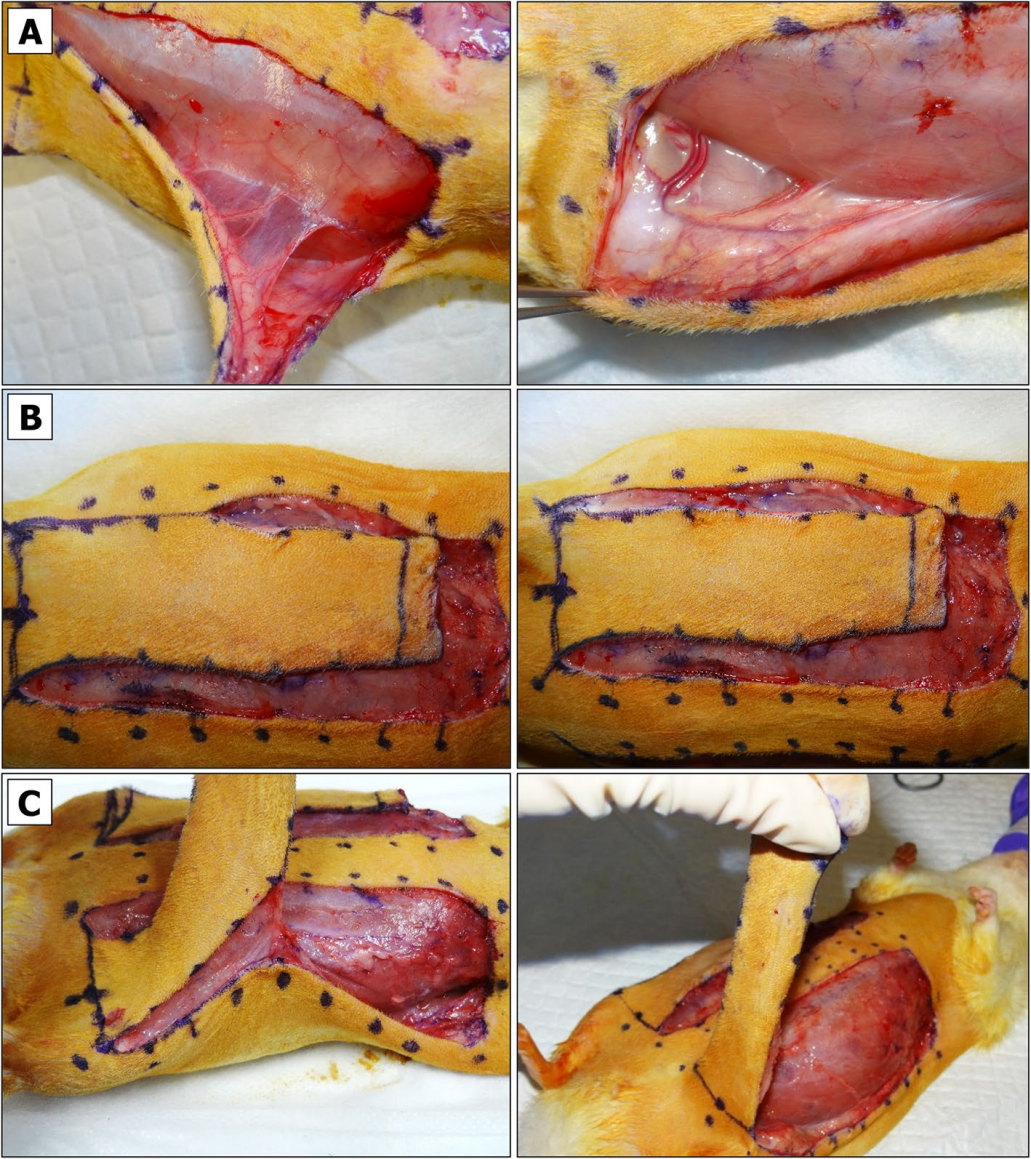
Raising BEFAF.** A** Full separation of fasciocutaneous tissue from the underlying muscle. **B** Superficial cutting of outer flap sidewall to preserve the lateral branch of SIEA vessel. **C** Flap positioning for electrocautery (left panel) and flap elevation on its base post-cautery (right panel)

1. An incision on the top of the flap is made using straight Iris Supercut scissors. The cut is deepened. A portion of biopsy area is released from the fascia using Mayo dissecting scissors (Fig. 2B).
2. The baseline (B_0_) biopsy area is lifted using Adson tissue forceps. This portion of skin is then cut out using Metzenbaum-Nelson scissors (Fig. 2C, left panel).
3. For HIS/IHC, the excised specimen is divided using a straight Iris Supercut scissors. The specimen is placed into a pre-labeled histology cassette, which is submerged in 10% formalin. For long-term tissue storage, the remaining specimen pieces are placed into the pre-labeled HDPE tube, which is sealed and submerged in LN_2_.
4. After a re-entry to the existing pocket, the tissue is dissected underneath the flap by slowly moving Mayo scissors towards the pedicle (Fig. 2C, right panel). It is important not to open the scissors too wide beyond the medial boundaries of the designed flap.
5. Straight Iris Supercut or Metzenbaum-Nelson scissors are used to cut through the inner sidewall of the flap up to its end (Fig. 2D, left panel). The same is done for the opposite side (Fig. 2D, right panel). Some portions of the flap may still be attached to the underlying muscle by vascularized fascia (Fig. 3A, left panel). If so, a flap is lifted, and the fascia is cauterized so that the flap can be freed up to its base (Fig. 3A, right panel).
6. Straight Iris Supercut or Metzenbaum-Nelson scissors are used to cut through the lateral solid line of the flap roughly to its midline (Fig. 3C, left panel). From this point forward only the superficial layer of the flap should be cut through to avoid the damage of lateral branch of the SIEA (Fig. 3C, right panel). The same is done for the opposite side.
7. The cautery device is used to cut through the tissue of the elevated flap until its base (Fig. 3D). The same is done for the opposite side.
8. Both flaps are returned into their original horizontal position.

### Physical Vessel Separation and Selective Clamping to Induce Primary Ischemia (PI)

**Fig. 4:**
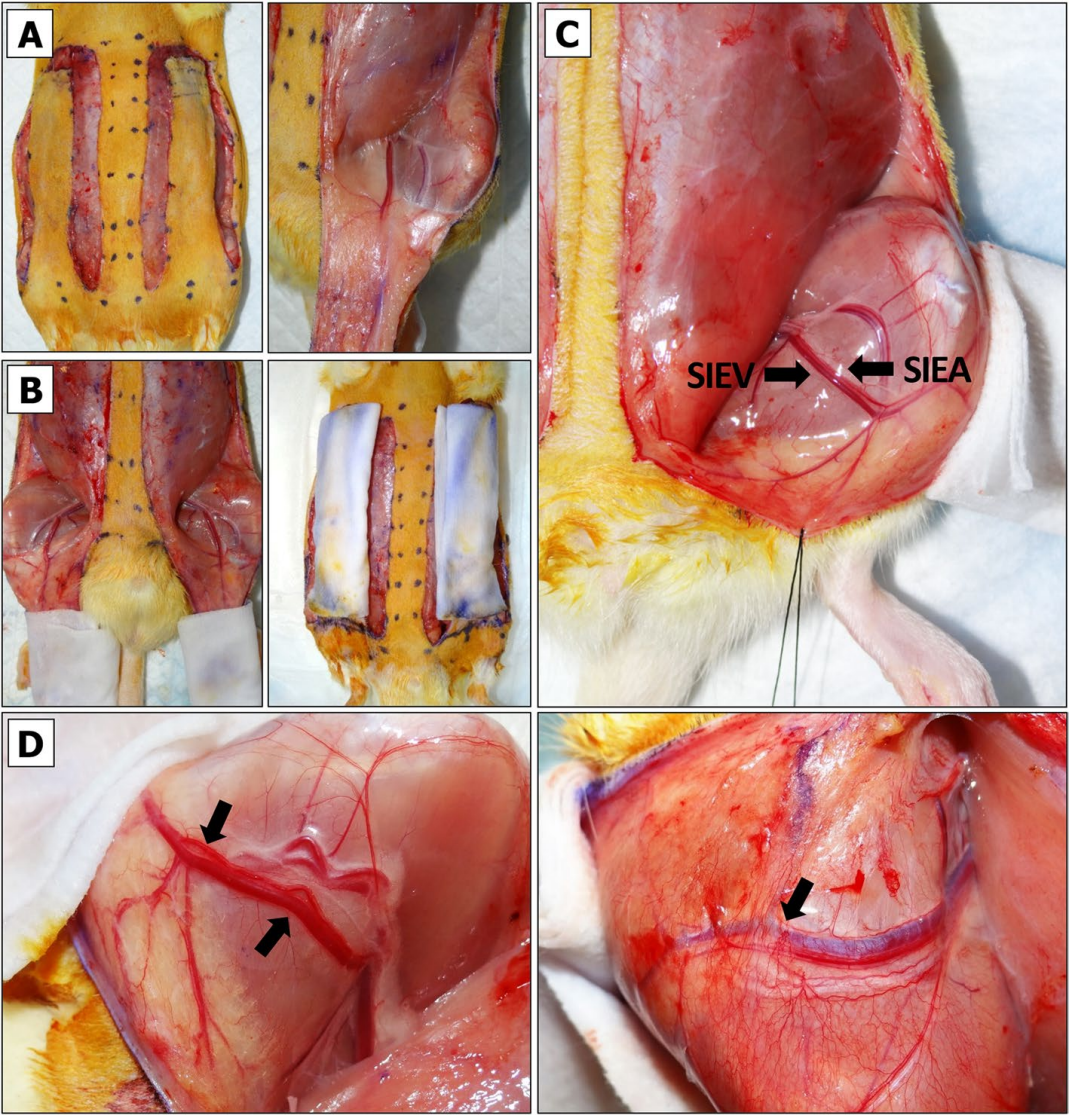
Exposure of BEFAF pedicle.** A** Flaps in their original closed and opened positions. **B** Flap irrigation. **C** Exposure of left pedicle once the flap is in an extended position. **D** Representative anatomy of the pedicle in female (left panel) and male (right panel) rats. Black arrows indicate the best target points to pass a suture for the most effective and safest SIEA and SIAV vessel separation

**Fig. 5:**
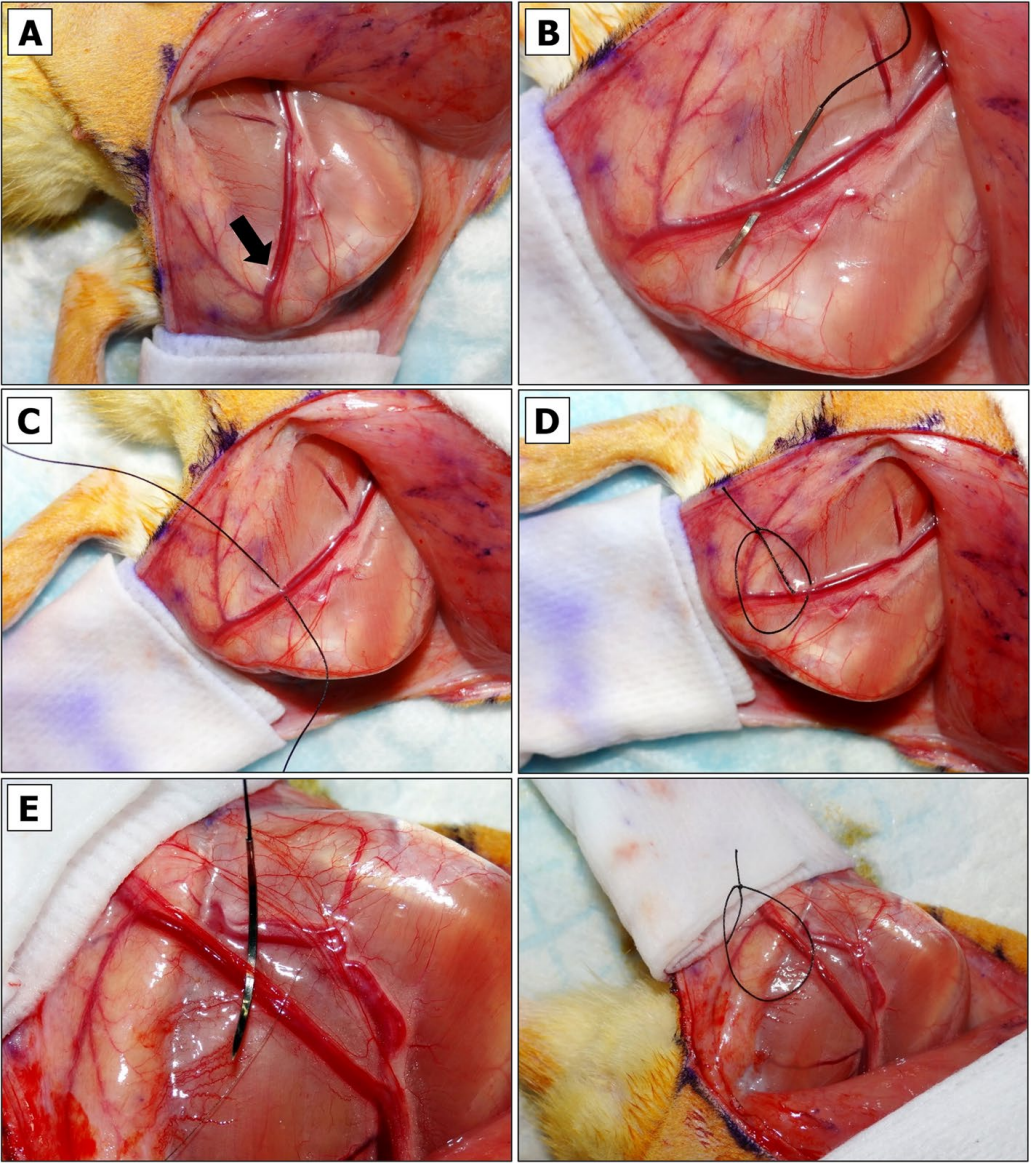
Vascular separation. For the isolation of SIEV, the largest gap between SIEV and SIEA is identified when the flap is in an extended position (black arrow, **A**). The needle is inserted from the outside of vessel bundle towards the middle (**B**). Silk suture is passed (**C**), cut and tied around SIEV (**D**). SIEA is similarly separated and isolated by looping the vessel on the opposite side (**E**)

**Fig. 6:**
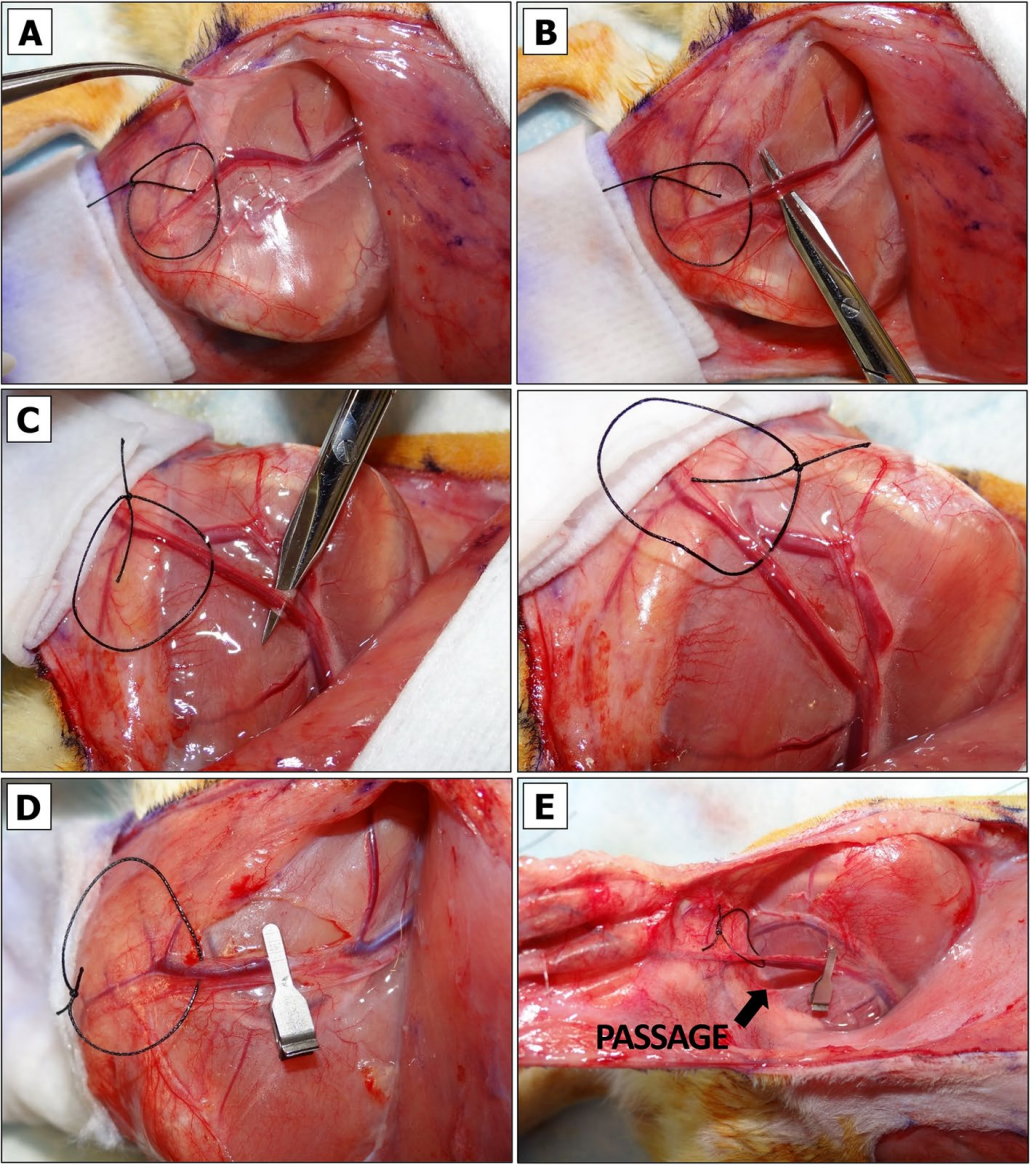
Induction of primary ischemia (PI). A passage underneath each pedicle is made (**A**-**C**) large enough for passing B-2 V clamp to occlude both SIEA and SIEV vessels (**D**). After clamping, the passage is widened to form a ~ 45–60° angle with flap’s base (**E**)

**Fig. 7:**
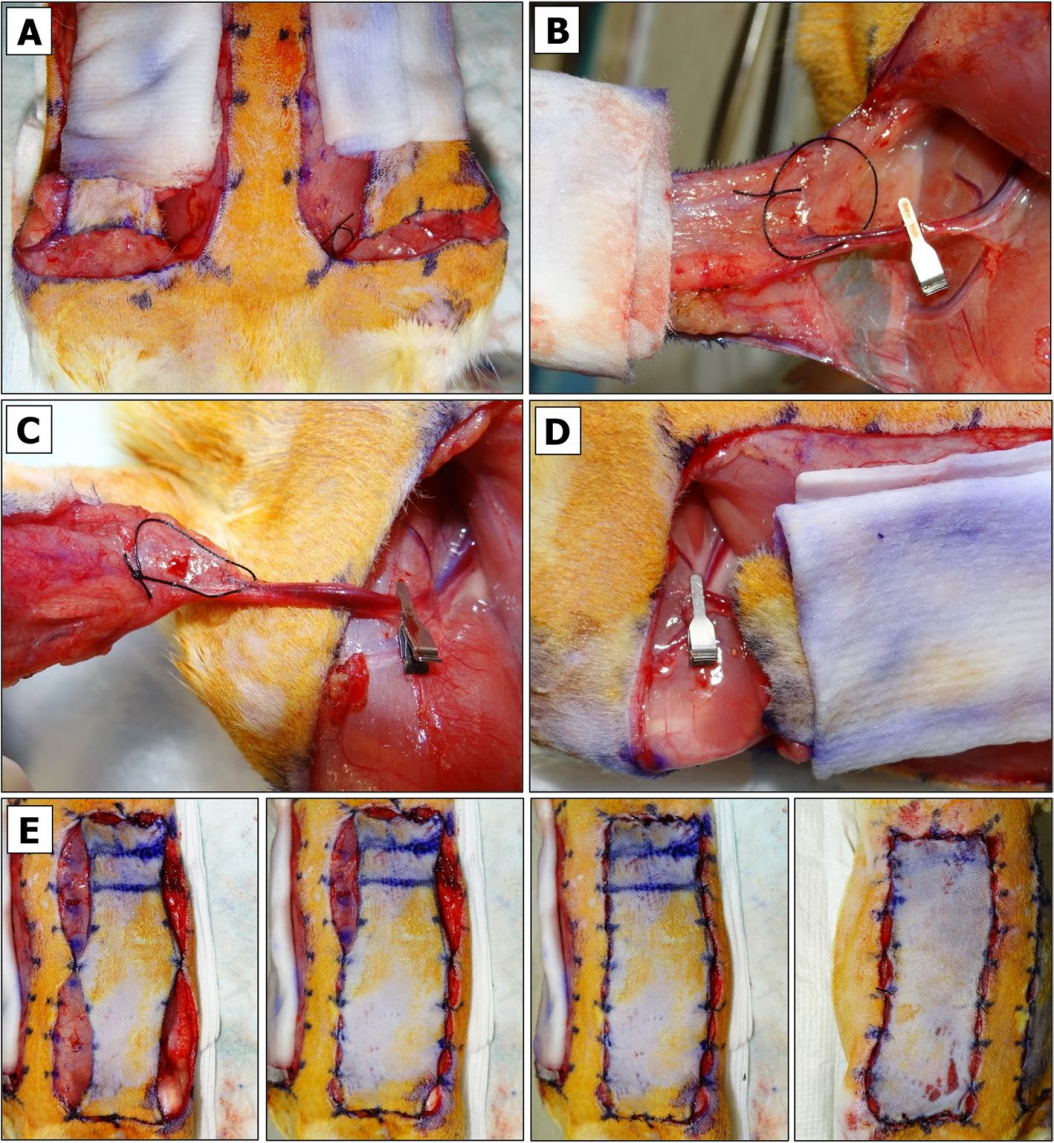
Isolation of the pedicle and temporary closure of the flap. A top (**A**) and rear (**B**) view of superficial cut of flap’s base. **C** An isolated single-pedicled flap after a complete removal of residual microvascular tissue. **D** Placement of isolated pedicle into its original position. **E** Steps for temporary left-side flap securing with non-absorbable sutures. **F** Top-view of right-side fully secured flap

1. The pedicle is exposed. Some rats have abundant fatty tissue obscuring a view to the emerging SIEA/SIEV bundle from femoral artery/vein. Such cases require a deeper dissection and removal of more fascia around the thigh and the vessels (Fig. 4A). Once the vessels appear clean, each flap is wrapped in sterile gauze moistened with sterile DPBS to preserve them from drying and return to the original horizontal position (Fig. 4B). NOTE: to minimize variation, the temperature of DPBS should be kept constant unless the objective is to compare the viability of flaps exposed to warm or cold ischemia.
2. Isoflurane flow should be decreased to 2%.
3. Holding the tip and the center portions of the wrapped flap, a flap is positioned over the animal’s thigh so that the pedicle appears in an extended position (Fig. 4C).
4. The SIEA and SIEV vessels are irrigated in 1% Lidocaine HCl solution applied dropwise.
5. At this point the vessels of the pedicle may be examined more closely. The most suitable site for physical SIEV or SIEA separation is based on vessel anatomy and the surgeon’s left- or right-handedness. Vessel anatomy in male and female rats slightly differs. In contrast to females (Fig. 4D, left panel), male SIEA may be less pronounced and run very close to SIEV without making large loops (Fig. 4D, right panel), which may pose a challenge for their separation without an aid of magnifying spectacles and LED headlight.
6. For SIEV isolation the Halsey micro needle holder scissors are used. Once the largest gap between the SIEA and SIEV before the point of SIEV bifurcation is identified (Fig. 5A), a 4–0 size needle with attached silk suture is passed between the SIEA and SIEV in a direction shown in Fig. 5B. When the needle shows on the opposite side of SIEV, the scissors are released. The needle is grabbed close to the tip and slowly pulled upwards until the suture is underneath the SIEV (Fig. 5C). CRITICAL: if one of the vessels gets accidentally punctured, the bleeding can be stopped by clamping, raising both vessels and ligating them with LigaClip tool. When properly ligated and cut in half, vessels should not bleed (see Additional File 2, Fig. S3A and Fig. S3B). A ligated flap may serve as a negative control for BEFAF survival. The flap (right or left) that has been ligated and the time of ligation should be immediately marked in the POAMS “Surgery notes” field (Additional File 1).
7. The suture is cut, and a loose knot is tied around the isolated vessel (Fig. 5D). NOTE: The 4–0 size needle should make a passage wide enough for a subsequent application of vascular clamp. It is possible to carefully widen this gap by using microscissors and lifting the attached suture loop, but it may increase the risk of vessel damage.
8. Similarly, the SIEA is separated on the opposite flap (Fig. 5E). If the SIEA forms a significant branch, a suture should pass right before the bifurcation point as shown in Additional File 2, Fig. S3C.
9. In the POAMS form (Additional File 1) the “Surgery Notes” field is used to indicate which vessel has been isolated by looping on the left and the right side of the animal.
10. Slightly or fully curved smooth jaws micro forceps are used to lift fascia. Then Iris microscissors are used to make a small tunnel below the tied loop on the top and bottom of the pedicle (Fig. 6A). This passage is widened by inserting the microscissors underneath the pedicle and slowly opening them to dissect the remaining tissue (Fig. 6B). This step is repeated for the opposite side (Fig. 6C).
11. Vascular clamp-applying forceps are used to insert one B-2 V vascular clamp onto the pedicle with an already separated SIEV (Fig. 6D). Using slightly curved microforceps, the flow below an applied clamp is gently pushed towards the femoral vein to make sure that the blood outflow from the vein is fully ceased. It is normal to see some backflow from the femoral vein. NOTE: If the venous blood surpasses the clamp, there may be too much fascia left around the vessel. If a problem persists after repositioning of a clamp, a second (smaller) clamp may be applied making sure there is no blood entrapped between the two clamps (see Additional File 2, Fig. S3D).
12. Once the clamp is in a proper position, a countdown timer is immediately set and started for the desired duration of the PI (e.g., 4 h).
13. Steps 11 and 12 are repeated for an opposite side. In the case of arterial occlusion, the curved microforceps should be used gently to push blood away from the site of the clamp going distally towards the flap. If the clamp is adequately applied, no influx of blood will be seen in the artery between the clamp and the curved microforceps.
14. The flap is held vertically by regular forceps, and the microscissors or fine-tip cautery is used to widen the passage by dissecting tissue along the clamped SIEA/SIEV bundle (Fig. 6E). The angle between the isolated clamped pedicle and the flap should be ~ 45–60°. The same step is repeated for the opposite side.
15. Both flaps are returned to their original horizontal position. The moisture level of the gauzes that are wrapped around each flap is checked. If needed, an additional amount of sterile DPBS is applied. If the underpad is wet, it should be replaced to avoid hypothermia of the animal.
16. At this point the flap is supplied by residual microvascular plexus. To remove this feed the entire base portion of the flap is cut superficially using the Iris Supercut scissors (Fig. 7A). NOTE: this step can be omitted for local/regional pedicled advancement or rotational flaps since these do not simulate a free flap situation.
17. The tissue at the base of the flap is cauterized while holding the flap vertically (Fig. 7B). At this point, the flap becomes connected to the animal only by the clamped pedicle (Fig. 7C). CRITICAL: Without pulling the pedicle (it may cause undue strain on the vessels), the flap is returned into its original horizontal position (Fig. 7D), and this step is repeated for the opposite side.

#### Partial Flap Closure

1. The 2% isoflurane flow should be decreased to 1%.
2. The flaps are sutured to the wound boundaries by using buried simple-interrupted suturing technique and non-absorbable silk suture (Additional File 2, Fig. S4) in the order and directions as suggested in Additional File 2, Fig. S5.
3. First, the bottom corners of a longer flap (which did not undergo biopsy excision) are sutured, and the same procedure is performed on a shorter flap (Additional File 2, Fig. S6). It is important to begin by suturing the base of the flap in order to minimize strain on the vascular pedicle.
4. Second, the upper corners of a longer flap are sutured followed by the center (Fig. 7E, left panel) and the remaining suturing points at the gaps (Fig. 7E, middle panel). A water-tight closure of the top and bottom parts of the flap is not necessary, but enough to prevent exposure of the clamp or vessel isolation loop and excessive accumulation of blood onto the future biopsies (Fig. 7E, right panel).
5. On the opposite side, the upper corners of a shorter flap are sutured using a non-absorbable silk suture. Finally, the center and the remaining points are sutured using an absorbable suture. NOTE: the guiding dots for suturing of this flap will not exactly match since the flap will be slightly advanced (Fig. 7F).
6. Isoflurane flow is decreased to 0% so that the animal would receive 100% oxygen. When it starts showing the signs of awakening on the operating table, it is returned to a cage to recover.
7. The instruments are cleaned from blood and dry-sterilized using the hot bead Germinator.
8. All waste is removed, and sharps are disposed in specified containers.
9. The cage is kept in the vicinity to monitor the animal’s well-being and activities more closely. If it starts extensively picking on the sutures, the Elizabethan collar should be applied.

#### Primary Reperfusion (REP_1_)

**Fig. 8:**
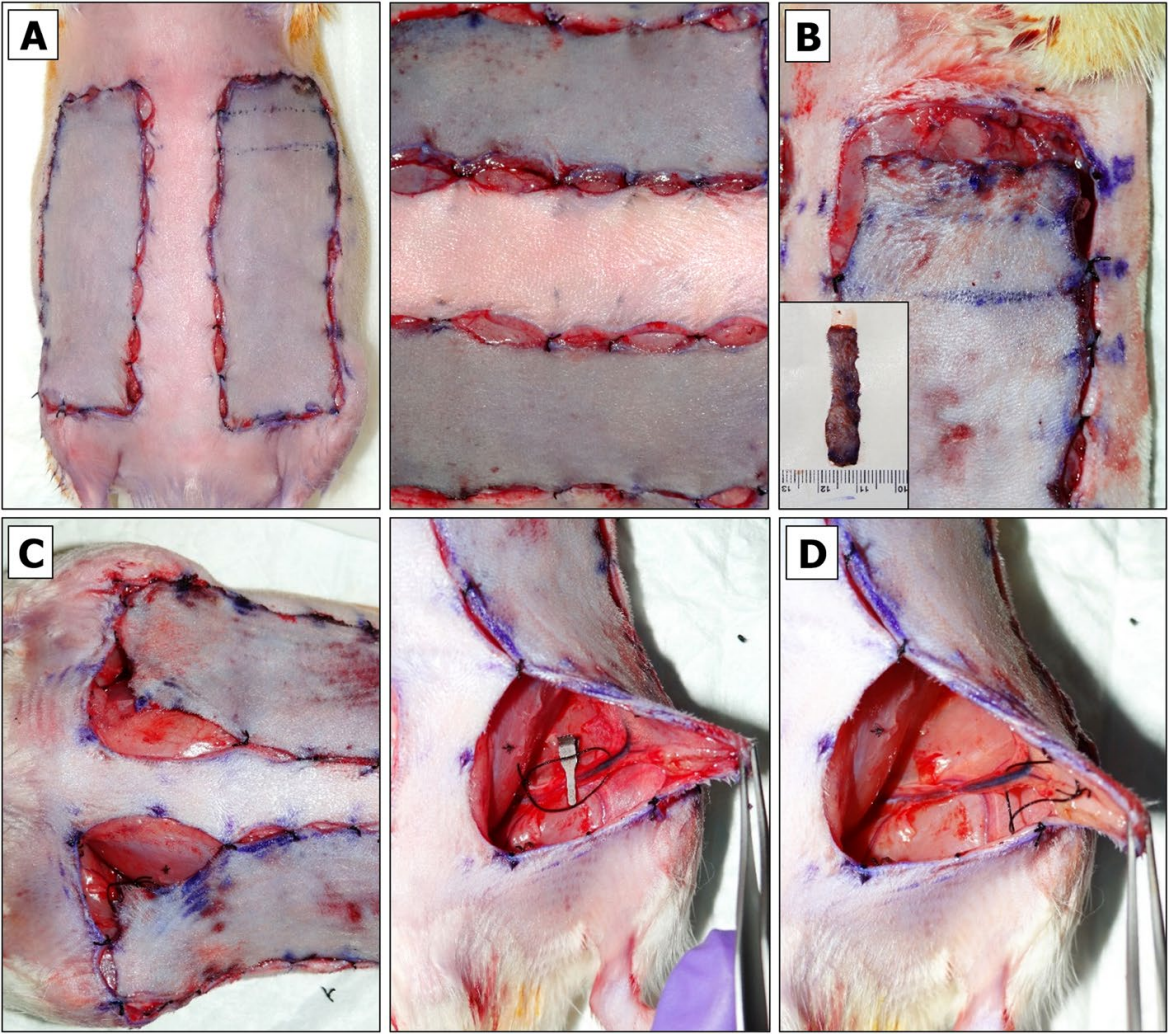
Primary flap reperfusion (REP_1_). **A** Macroscopic evaluation of BEFAF after 2 h (left panel) and 4 h (right panel) of PI. **B** B_PI_ biopsy excision. **C** Flap preparation for clamp removal. **D** Inspection of blood flow restoration in unclamped pedicle prior to REP_1_

1. Fifteen minutes prior to the planned end of PI, the animal is placed into the anesthetic chamber for up to 4 min.
2. Meanwhile, two new underpads are placed onto a heating pad.
3. 3% isoflurane flow is redirected into the nose cone.
4. The sedated animal is removed from the chamber and placed onto the heating pad in a prone position. The lubricate ointment is applied onto both eyes prior to turning the animal over in a supine position.
5. Isoflurane flow is adjusted to the maintaining 2% (for larger animal) or 1% (for smaller animal).
6. The flaps are gently wiped with DPBS-moistened gauze pad to remove any aggregated blood. Then the top underpad can be removed.
7. The degree of ischemia is inspected visually in both flaps. After 2 h of PI, flap color should be significantly darker than the surrounding skin, and even more so after 4 h of PI (Fig. 8A). NOTE: if the color does not significantly differ, it may indicate that the vascular clamping was insufficient or ineffective. With the extent of usage, the gripping power of reusable clamps gets weaker, and caution must be taken to prevent the clamp from falling off the pedicle in an active animal.
8. The suture is disrupted at points #4, #5, #6, #17 and #18 from the top portion of the longer (in this example, left) flap, followed by excision of the B_PI_ biopsy (Fig. 8B).
9. The sutures are removed from points #1 and # 9 (also #3 if needed more space) at the lower midline and an inner corner of the flap base on both sides (Fig. 8C, left panel).
10. Left flap is gently lifted (Fig. 8C, right panel) to remove the clamp by gripping it with the vascular clamp-applying forceps.
11. The pedicle is observed for restored blood flow (Fig. 8D). NOTE: we take note whether, upon clamp removal, there is some movement of venous blood. Small thrombi, if any, can be removed by applying a gentle sliding force with non-serrated microforceps. The vessels can be irrigated with 1% Lidocaine HCl solution applied dropwise after touching the pedicle.
12. Once the blood shows a sign of flow within the pedicle, the countdown timer is set and started for the desired primary reperfusion (REP_1_) interval (e. g. 2 h).
13. Steps 9 to 13 are repeated for the right flap. Then, flap corners are sutured back to their original position using silk suture.
14. The remaining sutures on the left flap are disrupted. The top portion is reconnected to the tissue using silk suture. IMPORTANT: Then, an absorbable suture is used to connect the center and remaining parts so that both flaps are advanced equally.
15. Steps 6 to 9 from “Partial flap closure” section are repeated.

#### Secondary Ischemia (SI)

**Fig. 9:**
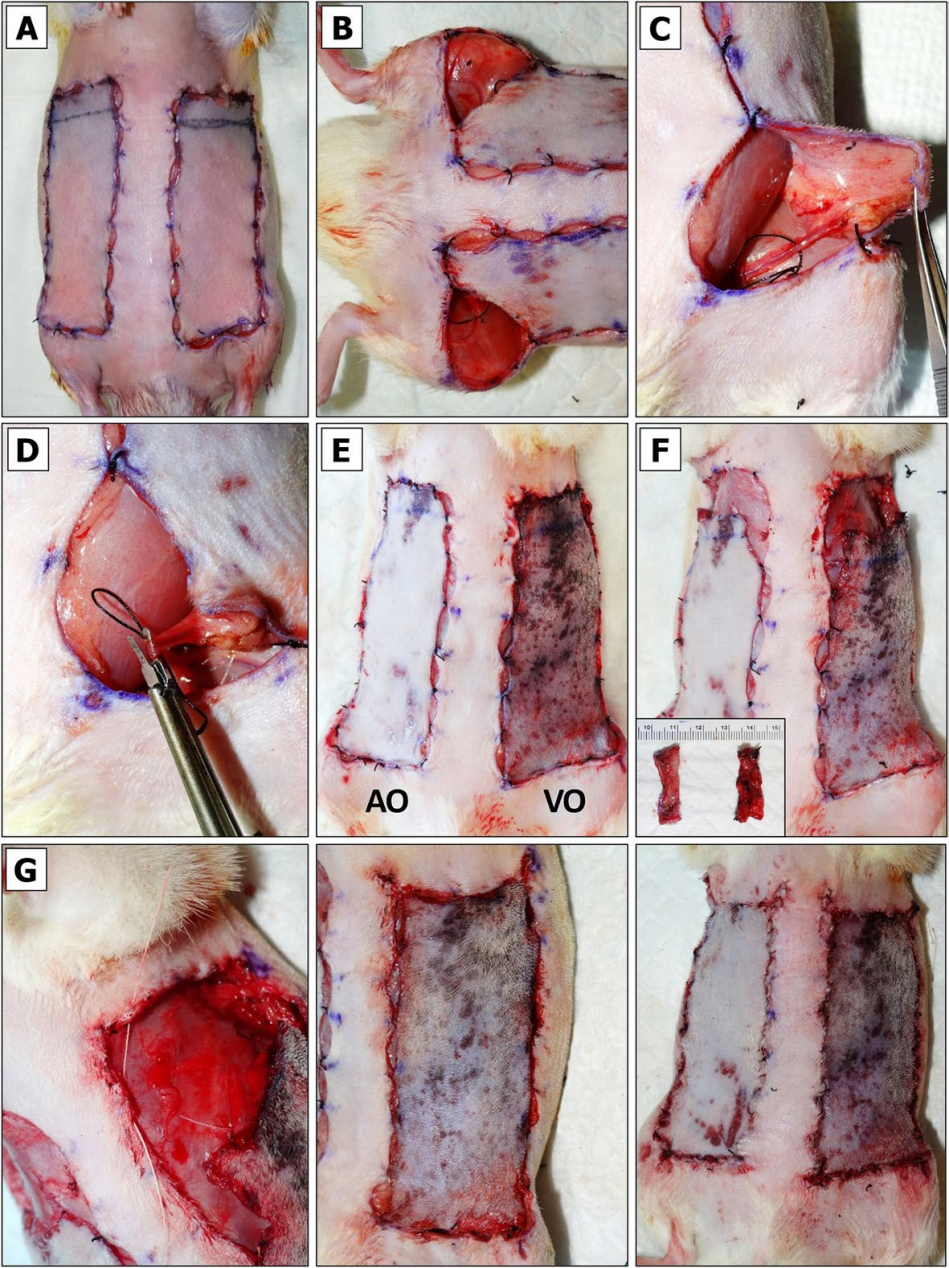
Induction of secondary ischemia (SI). **A** BEFAF appearance after REP_1_. **B** Flap preparation for selective vessel clamping. **C** Inspection of blood flow restoration in the pedicle prior SI. **D** SIEV clamping. **E** BEFAF appearance after selective SIEA (AO) or SIEV (VO) vessel occlusion. **F** B_AO_ and B_VO_ biopsy excision. **G** Final flap closure

1. Fifteen minutes prior to the planned end of REP_1_, the animal is anesthetized and prepared for the procedure as described previously.
2. 3% (for larger animal) or 2% (for smaller animal) isoflurane flow is redirected to the nose cone.
3. The appearance of both flaps is visually inspected to assess the approximate percent of restored blood flow. Figure 9A shows a case of efficient (~ 73%) reperfusion of a flap only 2 h after a designated 4 h PI time, which is well above the expected average [78].
4. The suturing points #2, #3 and #10 are disrupted at the lower sidewall and outer corners of flap base on both sides (Fig. 9B).
5. Left flap is gently lifted to inspect whether the blood flow is fully restored in the pedicle (Fig. 9C). Caution: some animals may develop spontaneous arterial or venous or mixed thrombosis after the PI (Additional File 2, Fig. S7). If a drop of heparin and 1% lidocaine does not resolve the thrombus and if the flow is not restored after a gentle push toward the groin area, the flap will be lost and should be excluded from the study. In such case, an opposite flap may be used as 4 h PI data point or any selected SI data point without a matching pair.
6. B-1 V vascular clamp is applied onto an isolated SIEV (Fig. 9D). While applying the clamp, the suture that separates both vessels is removed. The timer is set and started for desired venous occlusion (VO) period (e. g. 4 h).
7. B-2 V vascular clamp is immediately applied onto an isolated SIEA on the opposite side. The timer is set and started for desired arterial occlusion (AO) period (e. g. 4 h).
8. The bottom of both flaps is then closed using silk sutures.
9. Steps 6 to 9 from “Partial flap closure” section are repeated.

#### Secondary Reperfusion (REP_2_)

1. Fifteen minutes prior to the planned end of AO/VO, the animal is anesthetized and prepared for the procedure as described previously.
2. 2% (for larger animal) or 1% (for smaller animal) isoflurane flow is redirected to the nose cone.
3. The appearance of the flaps is examined to evaluate the efficacy of SI. VO flap should look significantly darker than AO flap (Fig. 9E). VO flap may be thickened by accumulated blood, which may form dark aggregates around the edges of the flap. These aggregates can be removed by flap irrigation with DPBS.
4. The sutures from the top portions of both left and right flaps are disrupted to excise the B_VO_ and B_AO_ biopsies, respectively (Fig. 9F).
5. The entire bottom of both flaps is opened widely to remove the clamps. The time of clamp removal is immediately indicated in the POAMS form to track the timing of secondary reperfusion (REP_2_).
6. The pedicles are observed for the restored flow. The thrombi are removed if needed, and both vessels are irrigated in 1% Lidocaine HCl applied dropwise.

#### Full Flap Closure

1. The bottom of both flaps is sutured using an absorbable suture.
2. The top of naïve wound bed tissue is narrowed by suturing the upper corners together (Fig. 9G, left panel). The top corners and top middle of each flap are connected to a narrowed tissue using an absorbable suture. Here one may use the corner stitch mattress suturing technique [79]. Next, the center and remaining intermediate points (Fig. 9G, middle panel) are connected together. The same is done for the opposite side.
3. At this point, PVA sponge implantation may take place. One PVA piece is inserted using Adson forceps underneath the P, M and D portions of each flap. Control sponges may be inserted in the outer area of a flap. Alternatively, a small pocket may be opened on the animal’s back.
4. A DPBS-moistened gauze is placed over the right flap. The left flap is positioned into place using a running suture technique with non-absorbable permanent suture (suggested directions of suturing are presented in Additional File 2, Fig. S8). The left flap is kept moistened and covered while the opposite side is sutured. NOTE: we do not recommend using proximate skin staplers with rotating head (e.g., Ethicon, #PRR35) to close the wound, especially in female rats that have thin and delicate skin.
5. The flaps and surrounding areas are wiped with moistened gauze to remove the traces of blood and betadine solution.
6. The animal is flipped into a prone position. 10 mL of saline is injected sc to supplement bodily fluids lost in the animal during this extensive surgical procedure.
7. Isoflurane flow is decreased to 0% so that the animal receives only 100% Oxygen.
8. The gross appearance of the flaps is recorded by digital photography (Fig. 9G, right panel).
9. Once the animal begins to awaken, we typically put a protective Elizabethan collar prior to returning the animal to a new padded cage with fresh food and water (Additional File 2, Fig. S9). The collar should be applied loosely so that it rotates around the neck.
10. The HDPE tubes with collected tissue specimen are placed in -80°C freezer for storage.
11. All waste is removed, sharps disposed in specified containers, and all surgical instruments are washed, cleaned, and autoclaved at this point.

### Post-surgical Animal Monitoring

When in distress, rodents can self-mutilate or cannibalize the ventral flap. If spotted on time, dehiscence may be fixed by suturing, but in the case of large opening the animal should be euthanized. When in doubt, the attending veterinarian should always be consulted. To prevent self-destructing behavior, Elizabethan collars should be used instead of restraining jackets that cause immobility and thus will compromise flap healing. Although male rats adapt to these collars quicker than female rats, a careful pre-operative introduction of the collars to all animals is strongly advised. Despite the pre-operative preconditioning with the collar, female rats will often refuse to eat/drink after the surgery but will quickly pick up on any exposed sutures once the collar is removed. Some leaner rats may access the bottom of the flap even in the presence of the collar (Additional File 2, Fig. S9). To avoid animal self-mutilation issues, many researchers choose to create the dorsal flaps as in the classical 1965 McFarlane experiment [80–84]. However, one should note that dorsal flaps represent RPFs and thus a different flap healing model compared to the APFs.

Surgery-related stress can be reduced by introducing an animal to a habitat with novel enrichment devices and dietary treats. While animal socialization is otherwise certainly encouraged, the post-operative animals (and in general retired breeder males) should be housed separately so that the collars may be used safely, and animal food/water intake would be tracked more accurately. Food pellets, dough diet and/or nutritional special needs recovery diet gels (commercially available from Bio-Serv (Flemington, NJ) or other vendors) should be placed on the floor postoperatively for easy access. Rodent diet may also be medicated. Post-surgical animal well-being should be constantly monitored at least twice per day. Animal weight should be recorded every other day. Post-surgical pain relief is available if it does not interfere with the aims of the study as some pain-relieving therapeutic compounds are known to impede or accelerate an inflammatory response and change the gene/protein expression patterns. The suggested example of POAMS is provided as Additional File 1.

### Non-survival Surgery

#### Preparation for Procedure

1. The HDPE tubes are labeled for appropriate specimen. A suggested label may contain a unique animal number, code for treatment, indication of left or right flap side (R/L), day of harvest and an abbreviated name of a harvested segment, e.g., “174,871, A, L7D”).
2. 10% buffered formalin container and histology cassettes are labeled for appropriate specimen.
3. A rubber bucket is filled with ice pellets. If PVA sponges were implanted, then 2.0 mL Eppendorf tubes are filled with PVA sponge lysis or other buffer, depending on the type of subsequent wound exudate (WE) analysis, and placed on ice. Flat-tip tweezers are prepared to handle the sponges.
4. Dewar flask is prefilled with LN_2_ for immediate tissue freezing. We suggest using curved HDPE tube grabbing scissors.
5. CO_2_ levels are checked in the euthanasia cart. Isoflurane and oxygen tank levels are checked in the anesthesia cart.

#### Flap Harvest

**Fig. 10:**
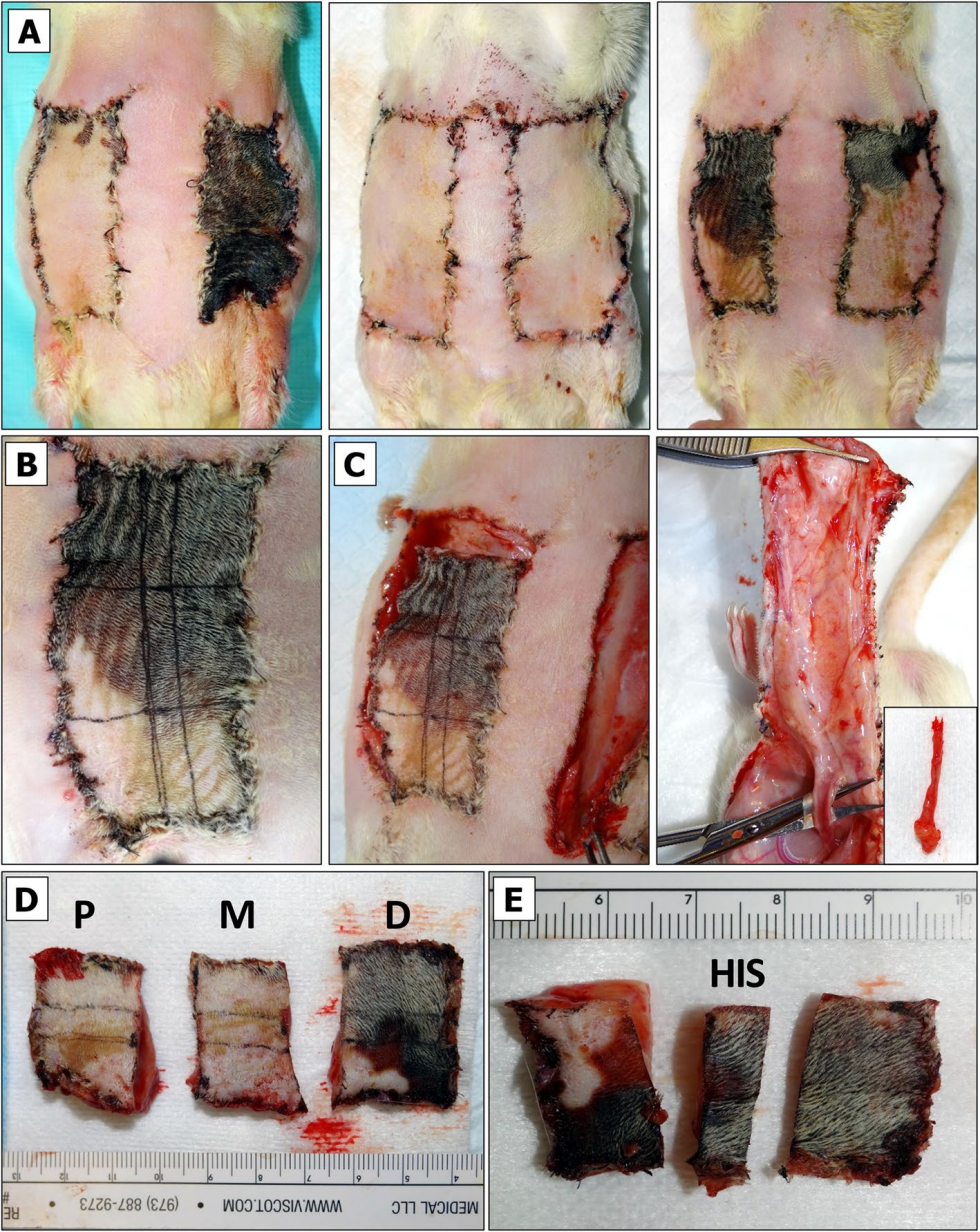
Flap tissue harvest. **A** Flap appearance and different surgical outcomes (full survival, partial or complete failure) at chosen end-point. **B** Flap division into thirds and longitudinal sections. **C** Removal of sutures and flap excision. **D** Inspection of pedicle function.** D** Physical flap division into the proximal (P), middle (M) and distal (D) portions.** E** Isolation of longitudinal section out of D portion of excised flap for the subsequent fixation and histological (HIS) or immunohistochemical (IHC) analysis

1. The animal is placed in the anesthetic sedation chamber prefilled with 4–5% isoflurane gas and kept there for up to 4 min.
2. 4% (larger rats) or 3% (smaller rats) isoflurane flow is redirected into the nose cone.
3. Once the animal is placed onto an underpad, which covers a heating pad, the digital photos of the flap appearance are taken. The functional outcome (full survival, partial failure, or total loss) of flaps exposed to SI should be evident by day 5, whereas flap fate following other treatments may be evaluated at later end-points. Figure 10A illustrates experimental situations, when the evaluation of flap survival percentage (FS%) by digital planimetry is not necessary (Fig. 10A, left panel – left flap, middle panel – both flaps) and when it is required (Fig. 10A, right panel – both flaps).
4. If the flaps are going to be divided into the proximal (P), medial (M) and distal (D) segments, a measuring ruler is placed on the top of a flap. Each flap is marked with either one dot, dotted or a solid line that would separate it into three equal portions. NOTE: this step should be performed on a living animal when the flaps are in fully extended position. If needed, the animal paws may be taped. If the IHC specimen is to be taken out of each portion, then two horizontal lines are drawn along the entire flap as shown in Fig. 10B.
5. All running sutures are disrupted and removed. One-by-one, the flaps are dissected along the wound margins and Mayo scissors are used to lift one flap after another from the wound bed. The status of the pedicle is inspected and POAMS form (Additional File 1) is used to indicate if it is functional or compromised. A fully functional vessel will be visibly dilated and pumping the blood (Fig. 10C). Obstructed vessel will have a dark blood clot or will be clear of the blood.
6. At this point, all PVA sponges may be retrieved if any were implanted previously. These sponges should be cleaned from the tissue prior to placing them into the pre-labeled Eppendorf tubes stored on ice. WE is collected though repeated compression of each sponge with flap-tip tweezers, and the samples are diluted if necessary.
7. If the flap is going to be divided into the P, M and D regions as shown in Fig. 10D, it should preferably be cut into the thirds while it is still attached to the pedicle. Alternatively, the entire flap may be cut at the base, transferred onto a sterile Petri dish kept on ice and divided over there. Optionally, the pedicle can be excised out of the P region for a separate histological evaluation of other types of analysis. Whichever technique the surgeon chooses, he/she should stay consistent and adhere to the chosen sequence of flap harvest procedure throughout the entire course of experiment.
8. For IHC, the middle longitudinal strip is cut out of each flap’s segment (Fig. 10E). A specimen is placed into a dedicated IHC casette skin facing up. Edema will be the most prominent in the P segment of the flap, making it the thickest segment out of three. CRITICAL: the tissue strip should be laid straight and should not be twisted. The cassette is submerged in 10% formalin and incubated at RT for at least 48 h.
9. The remaining flap segment is placed in a pre-labeled HDPE tube, which is then submerged in LN_2_. Even though the tissue may be small compared to the volume of the tube, it will less likely stick to the walls of the tube and will be easier to retrieve after freezing.
10. Other flap segments are processed in the same manner as described above.
11. For major vital organ harvest 5% (or greater) isoflurane flow is used to overdose the animal and then excise all organs of interest after its exsanguination. If organ harvesting is not required, then the gauze is placed over both excisional wounds and the animal is moved into the clean cage for euthanasia. The CO_2_ flow rate is adjusted at 5 L/min and exposure to CO_2_ is continued for at least five minutes after respiratory arrest. Euthanasia may be confirmed by cervical dislocation (for rats < 200 g in weight) or bilateral thoracotomy. NOTE: other methods of euthanasia may be used as per approved IACUC study protocol.
12. Animal carcass is placed in a biohazardous waste bag, which is disposed of according to Institutional LAS rules. Typically, this entails attaching a label with a specific information, logging animal death, leaving a carcass in a dedicated freezer or cold room, and concluding a unique animal cage tag.
13. The surgeon’s working area is cleaned and sanitized. All surgical tools are washed, placed in the autoclave pouches/bags or trays, and then sterilized in the autoclave.

## Results

The quantitative and qualitative analytical techniques of the collected biological specimens include but are not limited to planimetric flap survival analysis, histology (HIS) [85], immunohistochemistry (IHC), laser capture microdissection [86], flow cytometry (FC) [87], proteomic, metabolomic or transcriptomic analyses [88, 89]. These specimens may also be used to derive secondary analytes, such as EVs, which are typically analyzed for their biophysical properties (quantity, size, charge, optical redox ratio) and/or internal cargo consisting of proteins, lipids and nucleic acids [90–92].

The principles of planimetric analysis are described in Additional File 2, Fig. S10 and Fig. S11. Total and either necrotic or viable areas in the digital photos can be determined with the aid of a “Measurement Log”, “Quick Selection” and “Ruler” tools available in Adobe Photoshop or ImageJ software. The percentage of flap viability (FV%) or flap survival (FS%) is calculated as the necrotic flap area minus the total flap area divided by the total flap area (in square millimeters) multiplied by 100.

We also provide several simulated statistical flap survival data analysis examples in GraphPad Prism 9.5.0 software (Additional File 3 and Additional File 2, Fig. S12). The differences between the matched left and right flap survival may be estimated by the paired Student’s T-test (Additional File 3, Example 1, and Additional File 2, Fig. S12, upper left panel). The before and after treatment effects (e.g., baseline versus PI versus REP_1_ vs SI) within each flap of different animals may be evaluated by one-way repeated measures (RM) ANOVA (Additional File 3, Example 2, and Additional File 2, Fig. S12, upper right panel). For example, if the normalized phosphorylated Akt (p-Akt) protein expression effects were compared between the male and female animal groups consisting of 5 rats per each, we would be using two-way RM-ANOVA (Additional File 3, Example 3, and Additional File 2, Fig. S12, bottom left panel). A good source of explanation when and how to use fully-nested ANOVA without interaction is provided by John H. McDonald in his topic “Nested Anova” [93], which implies that if we would have 7 randomly selected rats, with 2 flaps created within each under different ischemic conditions, and if we would carry out 4 independent skin oxygenation measurements (one before the flap raising surgery in the spots of future-to-be flaps and then on POD 2, POD 5 and POD 9) with three repetitive measurements per each region of the flap each time, then our model could be analyzed either by “pure” Model II random-effects model-based nested ANOVA or by model I mixed-effects model-based nested ANOVA. In yet another example, if 14 male and 14 female non-littermate rats were randomly selected and assigned into 2 groups with 7 rats of each gender per each, and these groups would receive an external treatment (e g. tobacco vapors vs control water vapors) prior to BEFAF surgery, where one flap would be subjected to 4 h of PI alone and contralateral flap to 4 h PI, and then to 2 h of SI (e.g., AVO) after 12 h of REP_1_, the nested ANOVA model would treat male and female rats as sub-groups and their flaps—as sub-sub-groups nested within these sub-groups. Furthermore, the distinct P, M and D segments of these flaps, if measured individually, would be treated as sub-sub-sub-groups. But if an overall flap viability % was measured at a single time point, e.g., POD 9, we could apply three-way 2×2×2 RM-ANOVA (Additional File 3, Example 4, and Additional File 2, Fig. S12, bottom right panel). In the provided example, values stacked in sub-columns would represent a set of matched or repeated measures values (4 h PI vs 4 h SI). Means of cells that differ only by one factor would be compared using post-hoc Šidák test, reporting the multiplicity-adjusted p-values for each comparison at alpha = 0.05 (95% CI). Residual, homoscedasticity and quantile–quantile (QQ) plots would also be provided. An excellent feature of this type of analysis using GraphPad is the option to calculate cell/row/column means and grand means, which aid in plotting different results while considering or ignoring subject’s gender (see Additional File 3).

**Fig. 11:**
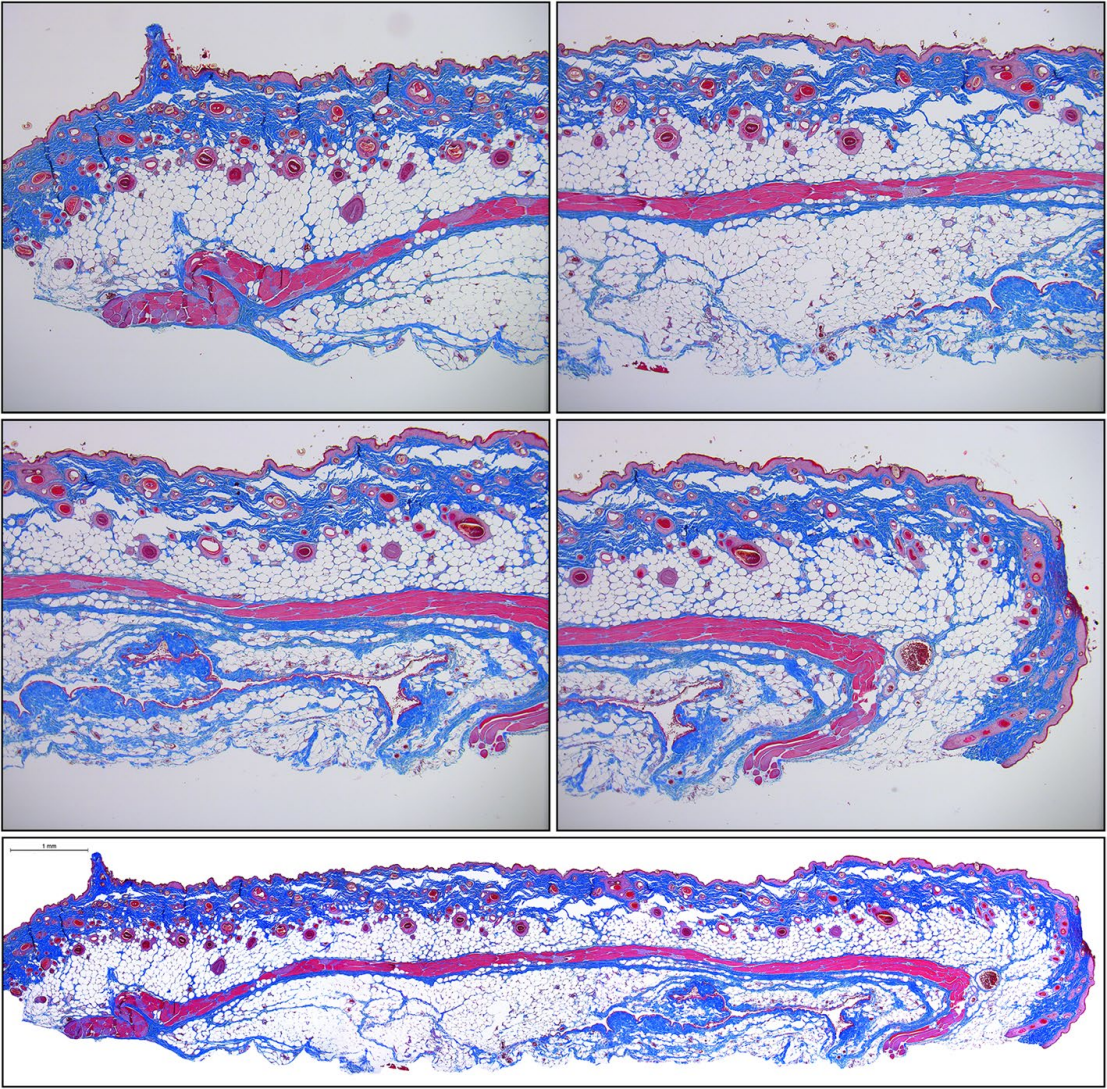
Individual unprocessed 4 × magnification micrographs of histological Masson’s Trichrome-stained sections of male rat’s middle left-side BEFAF segment before (upper panel) and after (bottom panel) manual image stitching procedure to generate one seamless image. This flap with an estimated 100% viability was harvested 48 h post-reconstructive surgery, where the left-side flap was exposed to 4 h PI, 2 h REP_1_ and 4 h of SI (AVO), whereas right-side flap was exposed to 4 h PI, 6 h REP_1_ and 4 h of SI (AVO). The digital stitching procedure started with opening all three raw unprocessed images in Adobe Photoshop and extending the canvas of the first image to the right side. Then a second image was dragged as a layer onto the first image trying to align and eliminate overlapping features. Lastly, the third and fourth images were placed underneath. The exposure of each layer was adjusted to match the previous layer using “Levels” properties. The darker edges were eliminated by using “Dodge” tool. Finally, the layers were merged and cropped to eliminate blank spaces on the top and bottom sides. The background of the final image was cleaned from debris by selecting the background with the “Quick Selection” tool, deleting it, and replacing it with the white color. The final image was then adjusted for contrast and sharpness. Scale bar = 1 mm

**Fig. 12:**
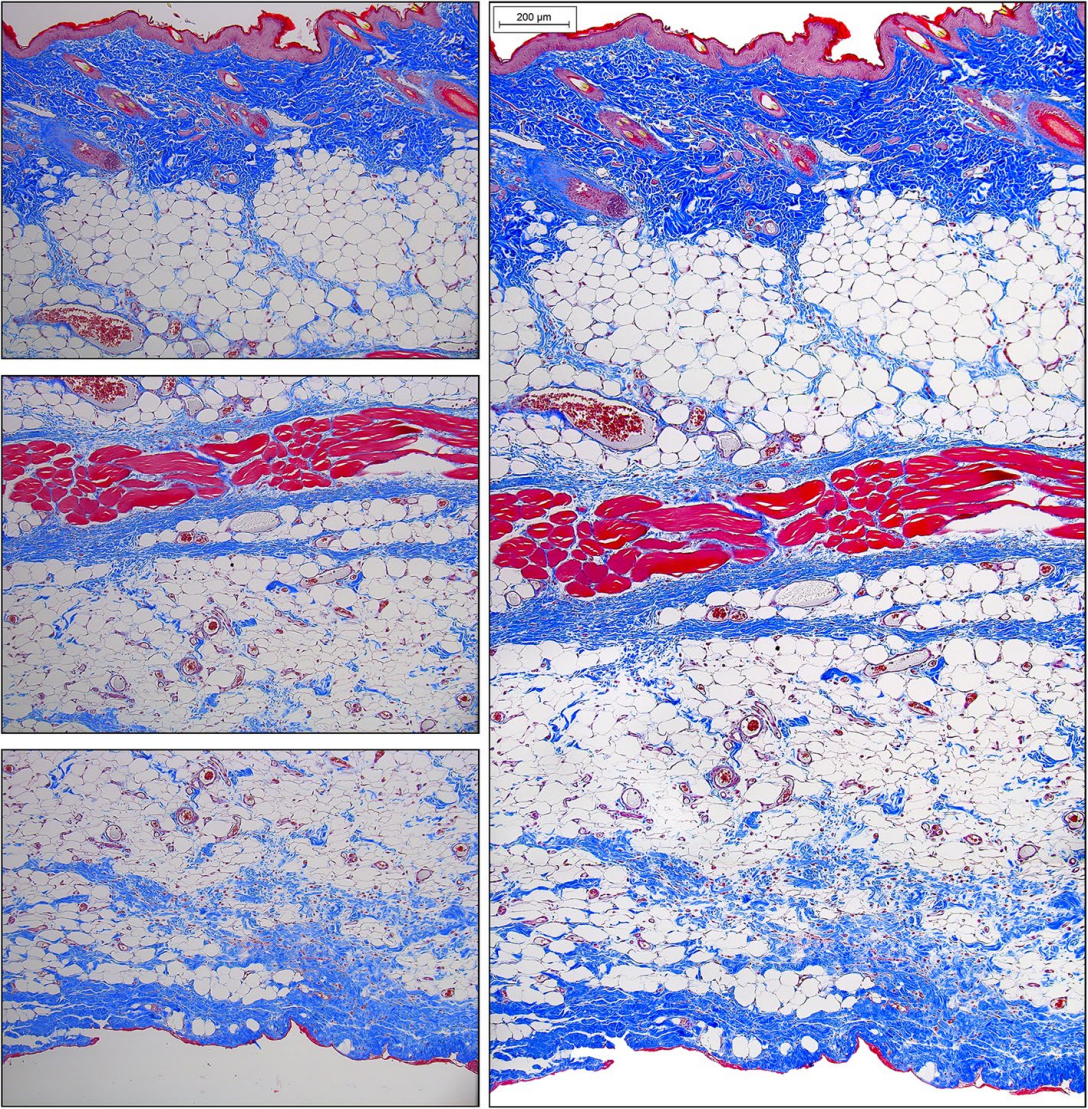
Individual unprocessed 10 × magnification micrographs of histological Masson’s Trichrome-stained top, center, and bottom layer of male rat’s distal left-side BEFAF segment before (left panel) and after (right panel) manual image stitching procedure to generate one seamless image. The digital stitching process starts with opening all three raw unprocessed images in Adobe Photoshop and extending the canvas of the first image down. Then a second image was dragged as a layer onto the first image trying to align and eliminate overlapping features (indicated by yellow arrows). Lastly, the third image was placed underneath. The exposure of each layer was adjusted to match the previous layer using “Image > Adjustments > Levels” tool by sliding the input levels to the left or to the right. The darker edges of each image (if any) were eliminated by applying “Dodge” tool. Finally, the layers were merged and cropped to eliminate blank spaces on the left and/or right sides. The final seamless image was adjusted for contrast, exposure, and sharpness. Scale bar = 200 µm

The tissue sections are quite large and there will be room to place only two replicate sections on each slide for HIS analysis. We prefer MT stain over H&E, as it readily reveals new and old collagen structures, extent of inflammatory infiltration, edema, and newly formed vessels. Depending on the degree of necrosis, the left and right sides of tissue section may appear heterogenous. If there is no possibility to digitize the slides using an automated or semi-automated hardware/software, then manual image collection via conventional or polarized light microscopy may be performed in a following manner: (a) the image of the entire flap segment is taken at the lowest (4x) magnification and medium to low sharpness; (b) the upper, middle, and lower sections of the P, M or D flap regions are taken at higher magnification (10× or 20x) making sure these sections slightly overlap; (c) The images are then combined together using Adobe Photoshop or any other graphic design software to produce one seamless high-quality image (Figs. 11 and 12).

**Fig. 13:**
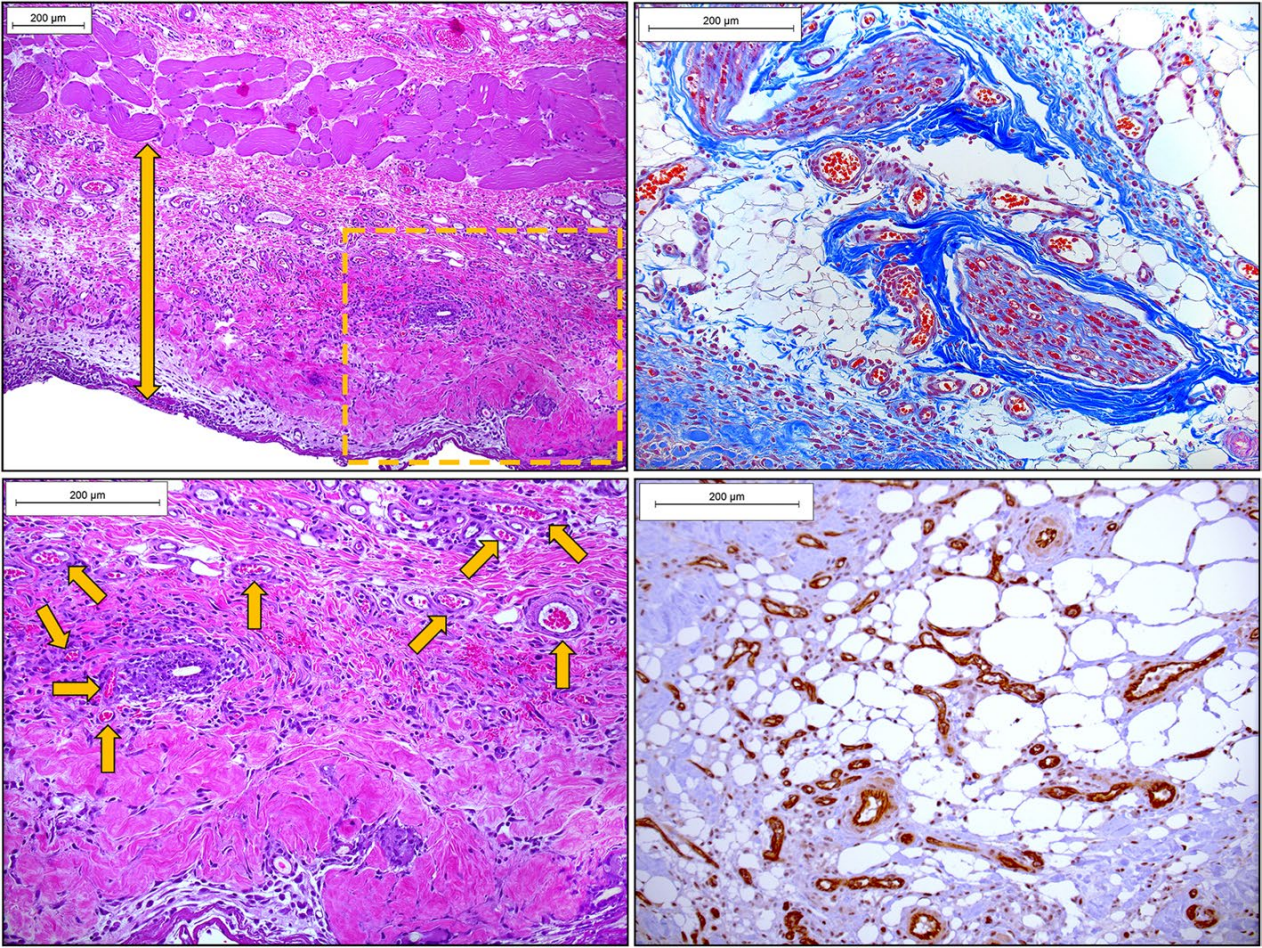
Left panel. Representative 10× (upper panel) and 20× (bottom panel) magnification micrographs of the bottom layer of Hematoxylin and Eosin (H&E)-stained histological section of male rat’s middle right-side BEFAF segment 7 days post-surgery, where both flaps were subjected to 4 h PI, 2 h REP1 and 4 h of SI, namely, VO (left flap) or AO (right flap). The deep fascia layer underneath *Panniculus carnosus* contains the granulation tissue (GT). GT thickness (indicated by yellow arrows) and vascularity (best visible in the 20 × zoom image of yellow rectangular region) will vary depending on the flap harvest time and the severity of the ischemic insult. Scale bar = 200 µm. Right upper panel. Representative 20 × magnification micrograph of the bottom layer of Masson’s Trichrome (MT)-stained histological section of proximal right-side BEFAF segment next to the pedicle 5 days post-surgery where both flaps were subjected to 4 h of PI, 2 h (right flap) or 6 h (left flap) REP_1_ and 4 h of SI (AVO). Both flaps survived 100% and had functional pedicles. At this magnification, the perfused blood vessels could be examined and counted manually. Bigger collagen-enclosed formations are regions of sprouting angiogenesis and neovasculogenesis. MT stain: blue color = collagen connective tissue fibers; dark red color – muscle and keratin; bright red color – red blood cells (erythrocytes); dark purple/black – cell nuclei; purple – cytoplasm; white – adipose (fat) cells. Bottom panel. The representative 20 × magnification micrograph of PECAM-1 (CD31) positive-vessels. 10% formalin-fixed 4 micron cut paraffin sections of the embedded distal segment of male SD rat flap 7 days after the BEFAF surgery, where the flaps were exposed to either 2 h or 6 h of PI, were stained with recombinant rabbit polyclonal anti-CD31 antibody (RM1006) (Abcam, #ab281583) at 1/400 dilution after heat-mediated antigen retrieval with Tris/EDTA buffer pH 9.0. Brown color reaction was detected with chromogen diaminobenzidine (DAB). Scale bar = 200 µm

**Fig. 14:**
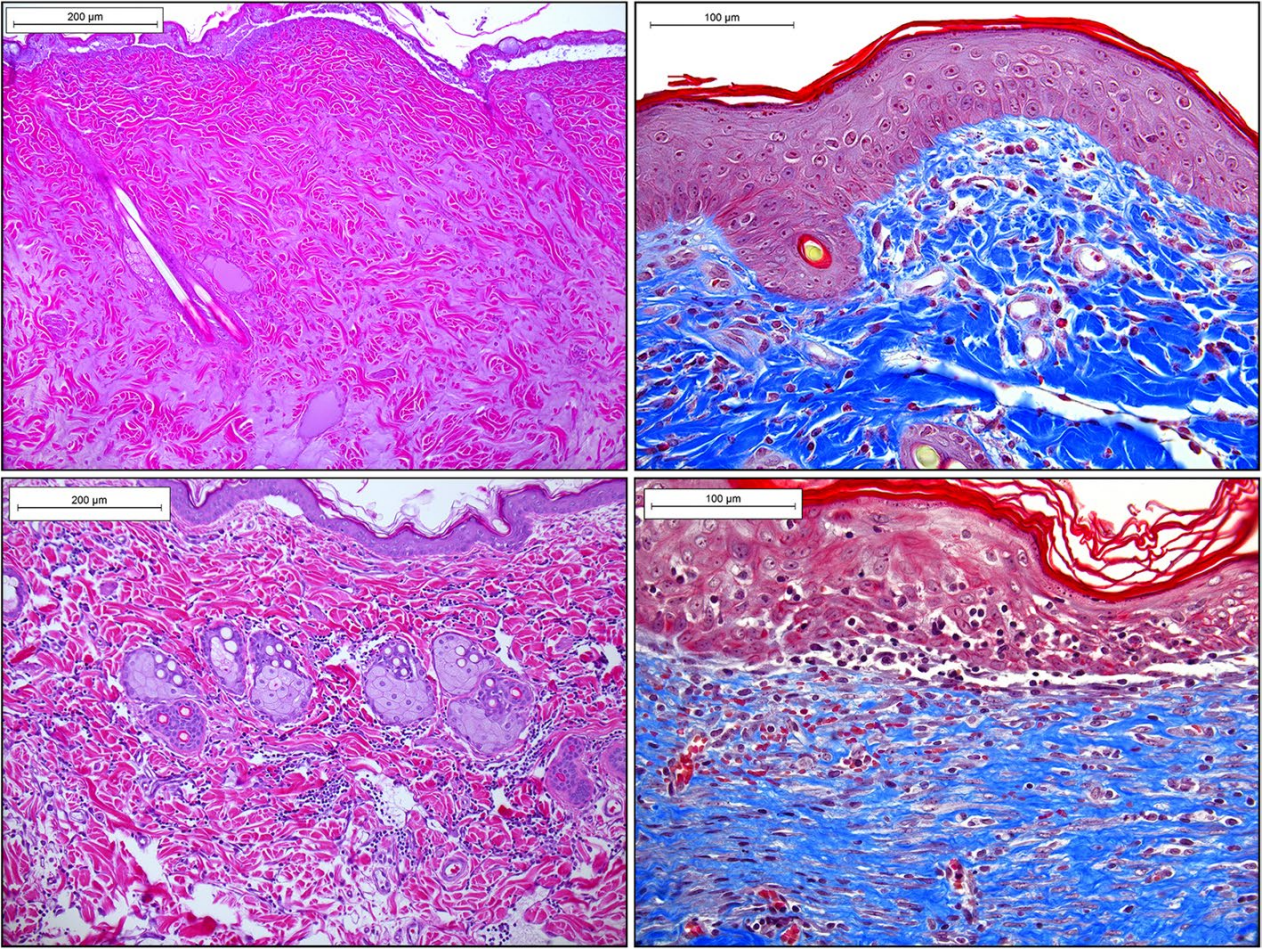
Left panel. Representative 20 × magnification microscopy images of top layer of Hematoxylin and Eosin (H&E)-stained histological sections of distal (upper panel) or proximal (bottom panel) left-side BEFAF segments 5 days post-surgery where both flaps were subjected to 4 h of PI, 2 h (right flap) or 6 h (left flap) REP_1_ and 4 h of SI (AVO). The D segment of left-flap failed which was confirmed by the dominance of acellular devitalized regions. The P segment of left-flap appeared healthy but showed a marked infiltration of inflammatory cells above the *Panniculus carnosus* layer. H&E stain: blue/dark purple – nuclei; pink – cytoplasm; magenta – collagen. Scale bar = 200 µm. Right panel. Representative 40 × magnification microscopy images of top layer of Masson’s Trichrome (MT)-stained histological section of middle right-side BEFAF segment 5 days post-surgery where both flaps were subjected to 4 h PI, 2 h REP1 and 4 h of SI, namely, AO (right flap, upper panel) or VO (left flap, bottom panel). At this magnification, the researcher can evaluate the integrity and health status of epidermis, as well as inflammatory cell infiltration into the upper layers of the skin. Scale bar = 100 µm

We stress the importance of presenting high resolution histological stain images of the flaps that are indispensable for diagnostic result interpretation in the full-thickness wound healing studies. Several images of the same area should be taken to reduce the risk of getting blurry poor-quality images (Additional File 2, Fig. S13). In our experience, the thickness of GT may be estimated from the 10 × magnification micrographs (Fig. 13, left upper panel), while 20 × and higher magnification micrographs are suitable for quantitative vessel analysis (Fig. 13, left bottom panel). While vascular density and angiogenic budding may be visualized in sectioned flap tissues stained with H&E (Fig. 13, left bottom panel) or MT dyes alone (Fig. 13, right upper panel), staining with specific endothelial phenotype markers such as PECAM-1, vWf and/or CD34 (Fig. 13, right bottom panel) may be preferred. Vascular inflammation that causes endothelial barrier disruption and tissue edema can also be studied using BEFAFs. Some proteins that are involved in endothelial barrier recovery, e.g. Hsp27 [94], are expressed in rat flaps (data not shown). 20 × and higher magnification micrographs are suitable for the gross tissue cellularity analysis (Fig. 14, left panel), including the presence of mature adipocytes and tissue infiltration with nucleated hematopoietic cells, namely neutrophils and macrophages (identifiable by their characteristic polymorphic and euchromatic nuclei, respectively, and by their positive staining reaction with CD45) (Fig. 14, right panel). Collagen deposition and collagen fiber alignment can be examined at 10 × magnification following MT or Sirius-Red staining (which can distinguish between different collagen types). Currently there are several automated free and open-source ImageJ and more sophisticated MATLAB software plugins created to analyze different types of scientific HIS/IHC images.

Additional File 2, Fig. S14 confirms that proteins implicated in flap tissue healing and regeneration processes (e.g., MMP-9, VEGF, Angiogenin etc.) can be isolated directly from the collected WE or from the fluid extracted from the implanted PVA sponges. The isoleucine and hydroxyproline quantity in acid hydrolysates of each PVA implant will reflect total protein and collagen content. Specific cell populations can be isolated by FC from the WE filtered through a 70 µm cell strainer. For instance, a purified (> 95%) population of wound macrophages as determined by F4/80 staining can be yielded by magnetic cell sorting using mouse anti-CD11b tagged microbeads [95]. A mixture of WE with specific lysis buffer to determine the latent and active matrix metalloproteinase profile by gelatin zymography [96] and various growth factor/cytokine/chemokine levels by ELISA [97] or IB may provide critical information on the healing trajectory of a wound and its suitability for advanced biological therapies [98–101].

Finally, one may choose to isolate proteins, mRNA or EVs from different flap segments using the materials and isolation procedures as suggested in Additional File 2. These biological cues may be analyzed in individual animals, but to minimize technical assay-to-assay variations, it is generally acceptable to summarize the individual animal responses following biological sample pooling.

**Fig. 15:**
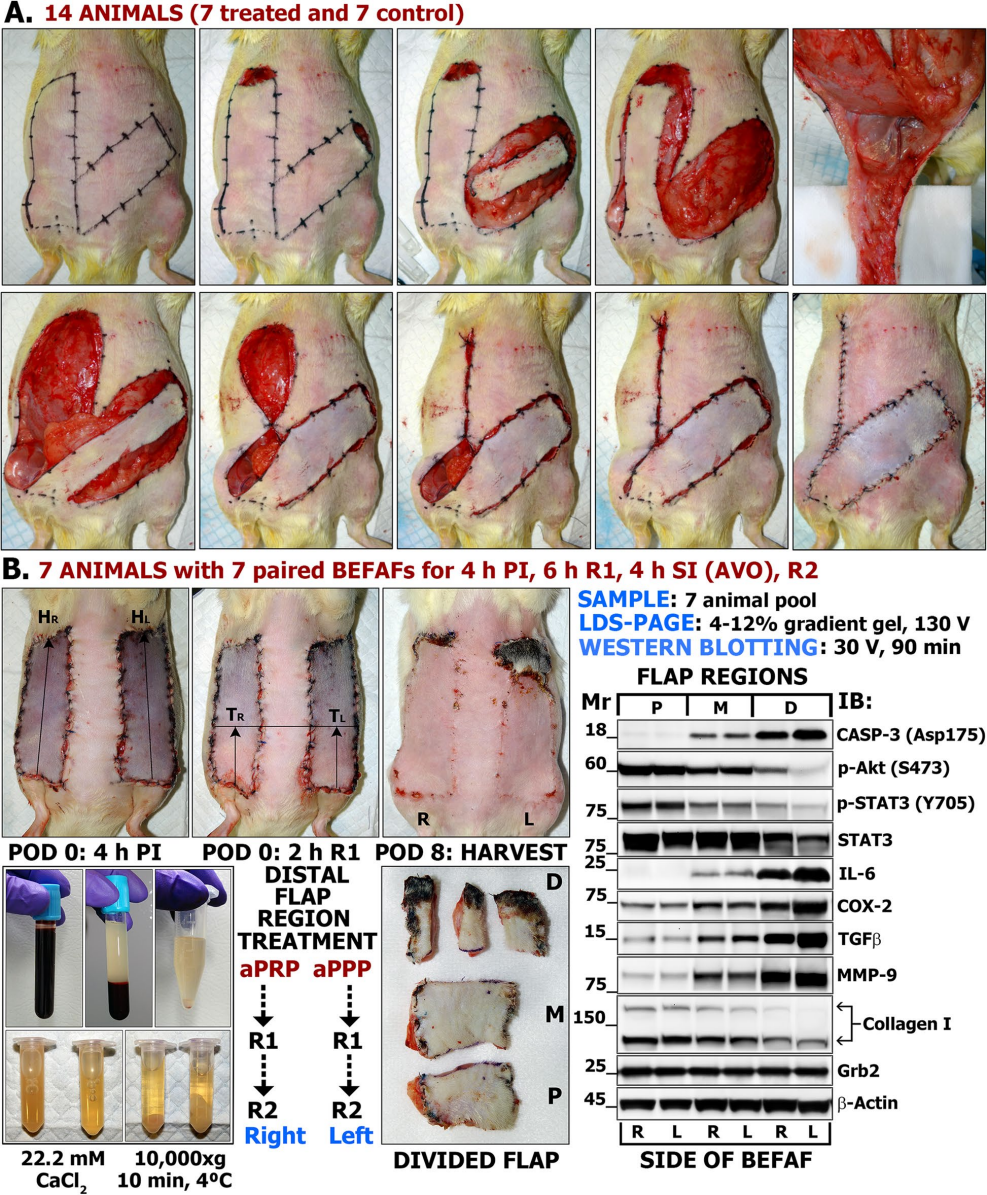
Application of conventional unilateral versus BEFAF model to explore the effects of prophylactic/therapeutic surgical flap treatment on tissue susceptibility to secondary ischemia. Experimental design: the SIEV and SIEA of the pedicle are clamped with a single atraumatic non-serrated S&T 11 mm vascular clamp for 2 h to induce primary ischemia (PI). After 2 h the clamp is released, and the flap is allowed to reperfuse for 6 h (R1) prior to repeated SIEV and SIEA clamping to induce a secondary ischemia (SI) by arteriovenous occlusion (AVO) lasting for 4 h. Once the SI is alleviated, the secondary reperfusion (R2) begins until the endpoint (POD 8). Experimental and control treatments are introduced prior to R1 and R2 events by intradermal injection into the distal flap region. **A** In a traditionally used ischemic flap model, a single fasciocutaneous flap is created and rotated around a pivotal axis point (the base of the pedicle may remain intact or may be severed to minimize a residual microvascular inflow) per animal. Power analysis suggests that if the flap survival percentage response within each subject after SI is normally distributed with standard deviation of 10, and assuming the attrition rate is 10%, then if the true difference in the experimental and control means is 20 as to be calculated by independent two-tailed Student’s T-test, we would need to study 7 experimental and 7 control subjects (total *N* = 14 animals) to be able to reject the null hypothesis that the population means of two groups are equal with probability (power) of 0.9. The Type I error probability associated with this test of this null hypothesis is 0.05. **B** Left panel. To perform the similarly powered experiment with twice as few animals per group, two 3 × 9 cm BEFAF flaps were created within each animal: one flap was assigned to the treatment, and the opposite flap – to the control group. Experimental treatment solutions were prepared from blood collected through cardiac puncture of previously sacrificed inbred Lewis strain rats (*N* = 12). Mixed arteriovenous blood was collected in 4.5 mL BD Vacutainer® Sodium Citrate (0.105 M, 3.2%) glass tubes that were centrifuged at 400 × g for 10 min at 4°C to separate plasma from red blood cells. Plasma with a buffy coat layer was collected into sterile 5 mL Eppendorf tube and spun at 1,000 × g for 10 min to sediment platelets and leukocytes. Half of supernatant was collected into 2 mL Eppendorf tube (*platelet poor plasma, PPP*). The remaining supernatant was gently mixed with the pellet and transferred into a separate 2 mL Eppendorf tube (*platelet rich plasma, PRP*). PPP and PRP solutions were activated by adding 1 M CaCl_2_ to the final concentration of 22.2 mM and incubating the tubes in a dry bath at 37°C for 1 h. Growth factors (GFs) were released from the precipitated platelets into the liquid fraction after final centrifugation step at 10,000 × g for 10 min. Supernatants were passed through sterile 0.22 µm pore size syringe filter, combined into a single aPPP or aPRP stock solution and stored at -20°C until further use. Pooling was intended to eliminate or minimize the heterogeneity of aPRP or aPPP content so that all flaps would receive an equal concentration of GF-enriched or control filtrate. 250 μL of aPRP was injected to the distal portion of inner right-side flap (R), while the 250 μL of aPPP (control) was injected in the similar location of the left-side flap (L) prior to the start of R1 and R2 events. At POD 8, the paired flaps were photographed, harvested, and divided into equal proximal (P), medial (M) and distal (D) regions that in turn were subdivided into left, central, and right segments. Right panel. The left segment of P, M or D regions was flash frozen, crushed into powder, lysed, pooled from seven 10-month old retired breeder Lewis strain male rats (mean weight = 542 g), and used for protein separation by lithium dodecyl-sulfate polyacrylamide gel electrophoresis (LDS-PAGE) at 130 V constant in 4–12% gradient NuPAGE gels followed by protein transfer onto 0.22 μm nitrocellulose membranes at 30 V constant for 90 min, and protein expression analysis by immunoblotting (IB) using the primary antibodies against indicated total or phosphorylated (p-) forms of antigens of interest and horseradish peroxidase-conjugated secondary antibodies. Chemiluminescent proteins bands were visualized by a Kodak Image Station 440CF equipped with the Caresteam image analysis software (Kodak, New York, NY) following 5 min strip incubation with 20 ml SuperSignal™ West Dura Extended Duration Substrate (TFS, # 34076)

Figure 15 demonstrates the bilateral flap model’s advantage over a traditional unilateral flap model in an experimental design where the superiority, equivalence, or non-inferiority effects of the desired preventative/prophylactic treatment is being evaluated. In this practical example of BEFAF model validation, the experimental treatment was based on activated platelet-rich plasma (aPRP), whereas a control treatment consisted of equal volume of activated platelet-poor plasma (aPPP). PRP is plasma enriched with a platelet concentration above that typically contained in whole blood. The platelets are best known for their importance in clotting blood. At the same time, alpha granules in platelets contain numerous growth factors (GFs) [102]. Besides their well-established roles in growth, migration, chemotaxis, division and differentiation processes, GFs can protect distinct types of body cells against ischemic or oxidative damage in vitro [103–107]. Consequently, different fields of medicine have increasingly experimented with aPRP substances containing 5–10 times greater amounts of GFs [108–110]. Upon release from calcium, thrombin and/or type I collagen-activated platelets [110], those cues have been shown to speed up the healing process of excisional full-thickness wounds [111–113]. aPRP administration has been shown to improve the survival of naïve pedicled RPFs or APFs in different experimental animal models in vivo [114–116]. Similarly, human patients with and without diabetes receiving aPRP injections or patch treatments saw a significant reduction in their wound areas [117, 118].

In a traditional experimental design, e.g., using a unilateral rotational APF as shown in Fig. 15A, a total of 14 animals (7 animals/group) would be theoretically required to evaluate the significance of experimental treatment effect on ischemic flap survival. But in the BEFAF model, where a distal portion of the right flap was treated with aPRP, while the left side was treated with control aPPP substances, animal number needs were reduced by half (Fig. 15B). The efficacy of PI, SI and reperfusion between the flaps could be visually documented to disclose any peri-operative variations of tissue response to physical manipulation (Fig. 15B, upper left panel). In the provided example, the efficacy of PI and the rate of primary reperfusion that was visually assessed after 2 h post-PI showed no noticeable difference between the left-side and the right-side BEFAFs (Fig. 15B, upper left panel, left and middle images). After 6 h both flaps were entirely perfused, and ready to be subjected to the SI insult (AVO). This is important, because in the event of significant dissimilarity or spontaneous thrombosis following the PI, the treatment outcome becomes arguably invalid. At the end of the study, marking a designated wound healing stage (e.g. at POD 8), FV% was evaluated by the planimetric analysis (Fig. 15B, upper left panel, right image). To standardize the detection of molecular and cellular tissue responses, the flaps were harvested and divided into three parts to separate D, M and P regions. Each region was then cut into three equal weight segments – the left segment is used for protein extraction, the right segment – for mRNA or EV isolation and the middle segment – for HIS (Fig. 15B, bottom right panel). In this experimental design, the control tissue could be obtained from the ventral unwounded centerline, and the dilated pedicle could be carefully dissected from P region for HIS evaluation.

As shown in the right panel of Fig. 15B, a vast array of rat species-reactive and compatible antibodies is available to assess tissue survival, inflammation, proliferation and even remodeling status by semi-quantitative Western Blotting technique. When assessing basal/constitutive or inducible signaling activation status of kinases, phosphatases, and especially transcription factors in injured tissues, it is a good practice not only to examine the extent of protein phosphorylation on their activating residues, but also to quantify the total (phosphorylated and non-phosphorylated) level of proteins, because their stability and expression dynamics may depend on wound healing stage as well as functional interactions with other proteins. For example, while the peak activation of signal transducer and activator of transcription protein-3 (STAT3) due to an inflammatory reaction and ischemia mostly occurs at the first stage of wound healing [119, 120], it is also prominent in later stages of wound repair, such as angiogenesis and re-epithelization as it can be re-activated by the vascular epidermal growth factor (VEGF) receptors [121, 122]. For example, from analysis of Fig. 15B, it can be suggested that despite sustained inflammatory cytokine levels (e.g., IL-6) which theoretically should activate JAK2/STAT3 pathway [123], the decreased phosphorylation of STAT3 on activating Tyr705 residues in the D flap region that failed to heal following severe ischemic insult and control treatment could be mainly attributed to the STAT3 protein degradation. This depletion likely occurred due to high protease activity and as a result of keratinocyte, fibroblast, and vascular endothelial cell death, as manifested by the augmented Caspase-3 Asp175 phosphorylation and severely delayed type I collagen fiber deposition at this later stage of wound healing, compared to the non-necrotic P region of the same flap containing high activated pro-survival Akt kinase levels that function upstream of proteolytic caspases. Caution must also be taken to interpret the fold-differences of protein expression results based on traditional house-keeping protein normalization. Some of those proteins might not be as stable as expected during the wound healing and deep tissue regeneration process. In our experience, the expression of growth factor receptor bound protein 2 [Grb2; UniProt database Accession No: P62994) and to a lesser degree, beta-Actin (UniProt: P60711) were relatively stable over the excisional large ventral wound healing process, whereas the glyceraldehyde-3-phosphate dehydrogenase (GAPDH; UniProt: P04797) and alpha-tubulin (UniProt: P68370) levels fluctuated a lot and were not suitable for protein normalization.

**Fig. 16:**
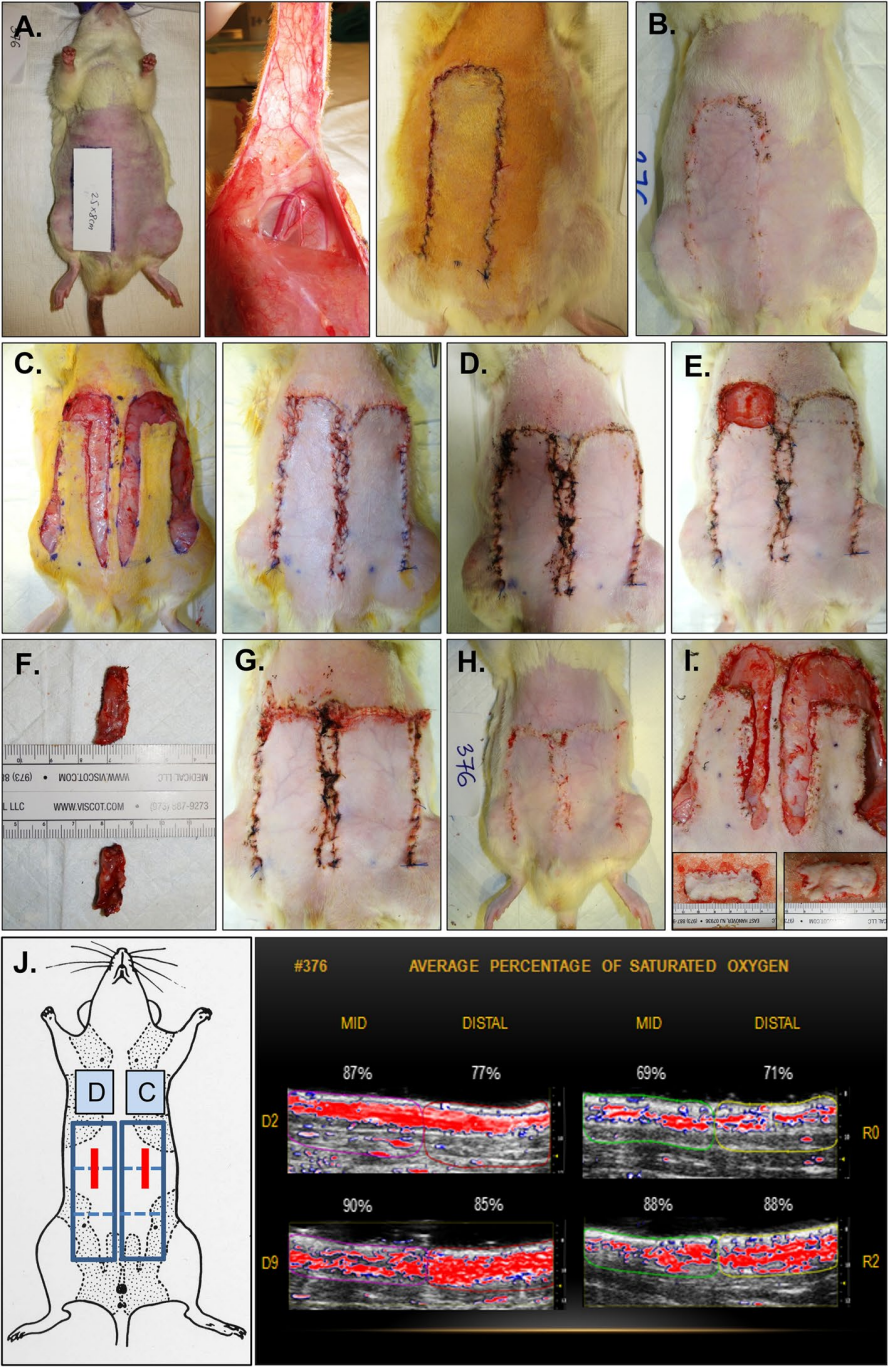
Application of BEFAF model to explore the effects of pre-conditioning surgical flap delay technique. A representative workflow of flap delay technique using pedicled flaps in 10-month-old male SD rat is shown. **A** Left flap was raised, placed back onto the resultant wound, and left to fully heal for 7 days. **B** Delayed flap healing status on 7th POD. **C** BEFAF surgery was performed on the left and right sides of the animal creating regular non-advanced flaps. During this type of surgery, the microvascular supply at the base of the flap (pedicle) is left intact. **D** Flaps were allowed to heal under normal conditions for 2 days. **E**–**F** On 2nd POD for the right flap and on the 9th POD for the delayed left flap, biopsies were taken from the distal portion of each flap for molecular analysis. **G** The flaps were advanced and allowed to heal for 7 more days. **H** Flap healing efficacy was evaluated on 9th POD for the control (C) flap and POD 16th POD for the delayed (D) flap. **I** Flaps were harvested for additional comparative and correlative analysis with flap reoxygenation that was evaluated by the combined photoacoustic (PA) and Doppler ultrasound (US) imaging (**J**). Ultrasound coupling gel was applied to the animal skin before the images were captured on selected regions (left panel) at the 12 mm focal depth using the Vevo2100 LAZR imaging system (VisualSonics Inc, Toronto, Canada), which operates LZ-250 transducer (21 MHz) frequency transducer and has 20 Hz tunable near-infrared light laser (680–970 nm). Besides B-Mode, which displays a greyscale two dimensional cross-section of tissue, 2-D images were acquired using ‘Oxy-Hemo’ mode, which collects data at 750 and 850 nm wavelengths, and then creates and displays a parametric distribution map of oxygenated Hemoglobin (oxyHb) and total hemoglobin (tHb) at a rate of 1 Hz (right panel).The imaging probe was positioned between the medial and distal segments on 1) the day of flap delay surgery on the R flap to take baseline flap oxygen saturation level measurement designated as R0; 2) on 2nd POD after left-side flap delay on the D flap to take acute reoxygenation response measurement designated as D2; 3) on 2.^nd^ POD after right-side flap raising surgery on the R flap to take acute reoxygenation response measurement (R2) and on the same day on the D flap to assess a secondary reoxygenation response designated as D9

In a similar fashion, BEFAFs may be utilized to evaluate the biological effects of a preconditioning surgical delay technique, which triggers the expression and activation of angiogenic genes [124] and potentially stimulates cell-to-cell communication through EVs. In this procedure, the left or right flap is raised, placed back onto the resultant wound, and left to fully heal for 7 – 14 days. Thereafter BEFAF surgery is performed on both sides of the animal creating a desired type of flap. These flaps are allowed to heal under physiologically normal or perturbed conditions. Pre-surgical and post-surgical responses of interest (e.g., the rate and degree of tissue reoxygenation) as well as tissue survival are then compared between the control (acutely raised) and paired experimentally pre-conditioned (delayed) flap tissue at designated POD(s) (Fig. 16). The expected EV size distribution derived from 2 g of naive abdominal flap is shown in Additional File 2, Fig. S15, proving that the isolation, separation, and detection of relatively polydisperse crude small and large EV sub-population fractions from collagenase-digested fasciocutaneous rat tissues is feasible.

## Discussion

Undoubtedly, both small and large animal models play a vital role in biomedical and veterinary sciences research. The significance, as well as limitations, of small animal models have been discussed in multiple reviews, now spanning decades [125–129]. While some concerns and debates have been raised regarding poor pre-clinical animal model translation into human clinical trials or routine medical practice [130, 131] potentially due to the lack of evaluation of methodology in the surgical animal studies [129, 132, 133], the scientific and didactical value of such models cannot be denied. Animal models have historically been used for basic and applied research, testing the tolerance and efficacy of pre-clinical treatments, implants and medical devices, and toxicity screening during pharmaceutical drug or vaccine development [43, 134]. They are essential for exploring cell survival and death mechanisms, as well as learning/training specific techniques and acquiring tissue handling, suture ligation, anastomosis skills, and more [135–137]. Surgical animal models have been instrumental in understanding the intricacies of diabetic ulcer and burn wound healing, cartilage repair, multifactorial musculoskeletal and cardiovascular disorders, vascular grafting choice, influence of co-morbidities and IRI on superficial or excisional wound closure, deep tissue repair and regeneration capacity. These models contribute significantly to the fields of transplant, plastic, and reconstructive surgery, extending beyond learning basic anatomy or microsurgery techniques [135, 138–140].

The standardized and continuously updated protocols detailing the scope and duration of procedures aid as effective teaching tools. They help in establishing and maintaining a reproducible animal model suitable to answer fundamental basic and translational research questions, gaining competence in pre-operative/post-operative animal care, post-operative complication handling techniques, and learning to apply the three R’s (Reduction, Refinement, Replacement) principles [134].

However, tissue flap models that aim to teach specific surgical skills and simultaneously study reparative processes still lack firm guidelines and standardization [141, 142]. The presented reconstructive surgery model using BEFAFs shows promise as a more effective, less time consuming and cheaper alternative for standardized validation of therapeutic targets, diagnostic and prognostic biomarkers as well as clinical treatment strategies than conventional unilateral wound healing animal models. This model is based on compound APFs and can be readily modified to represent either pedicled or free flap under varied clinical situations. One may assess the impact of endogenous or exogenous factors such as sex/gender, age, diet, physical activity, metabolic and chronic diseases on quantitative measures such as the rate of flap reoxygenation, vascularization, cell metabolism, migration, signaling and overall tissue survival. The BEFAF model is ideally suited to explore the molecular profiles associated with ischemic susceptibility or tolerance and to evaluate biomarkers for early prediction of flap outcome. Moreover, the procedure can be scaled and reproduced using other animal species (e.g., mice, rabbits, minipigs).

The concept of a model featuring symmetrical, three-sided, caudally based bilateral skin flaps in rats was first described by Ozcan et al. [143]. Their model created the RPFs resulting in a predictable and persistent necrosis. Elegantly, these RPFs have been used to assess the efficacy of preventative therapy (superoxide dismutase). Similarly, the tolerance of bilateral RPFs to the absence of vascular flow through the main feeding vessel and to the genetically modified autologous fibroblast therapy was investigated by Machens et al. [144]. Because these previous studies utilized RPF model, the flaps did not undergo a transient PI event – a prerequisite if one must mimic the autologous tissue transfer and IRI. In addition, the previously described models were not adapted to take intermittent biopsies for longitudinal or repeated measures data analysis and did not simulate temporary flap failure and salvage procedure.

As demonstrated by Figs. 15 and 16, BEFAFs, which effectively simulate the microvascular free flaps subject to spontaneous vascular complications, can be used to reveal effective therapies for prevention of flap loss or improvement of flap survival after re-exploration and salvage. Aside from an evaluation of flap healing rate, the animals may successfully undergo non-invasive in vivo microscopy. For instance, differences in left- and right-side flap vascularization and reoxygenation rates may be examined by combined photoacoustic and Doppler ultrasound imaging, while dynamic metabolic changes in tissues may be assessed by intravital microscopy [84, 145]. Temporal levels of intradermal nitrogen or oxygen may be measured by using specific needle-type microsensors or implants [146].

Finally, the BEFAF model can be adapted to evaluate a relative contribution of the recipient wound bed to APF survival. In such a case, a sterile silicone sheet is placed between the flap and the abdominal muscle. The sheet is sutured to the edges of a flap to prevent its movement, which may cause a physical damage to the pedicle [147]. Although some wound bed isolation studies conducted on single animals showed a significant reduction of flap survival rates [148, 149], other investigators did not see a significant difference in functional flap outcome, but agreed that induction of potent pro-inflammatory response by a foreign material might conceal the true driving forces of the healing process [150]. Ultimately, if superficial wound healing—not flap survival—is a preferable measure of outcome, then unipedicle or bipedicle ventral bilateral flaps can be created to establish a gradual ischemic environment followed by punching a series of excisional wounds. These wounds may be further reinforced using silicone splinters to overcome the excisional wound healing by contraction [151]. A similar bilateral flap approach may be applied to the dorsal RPFs that are described in the classical 4 × 10 cm McFarlane’s model [80, 152]. Theoretically, even APFs may be rotated along their axis to end up on the dorsal side and vice versa: the caudally based RPFs may be transposed towards a ventral side, but we did not test whether such transposition flap model is feasible, and it remains unclear if rodent skin would be flexible enough to cover two simultaneously created excisional wounds.

While the results of standardized studies using BEFAFs may be appropriate to guide recommendations for refining perioperative and post-operative flap management in specific human patient populations undergoing extensive reconstructive surgeries, this model presents challenges and has some limitations. The first or foremost challenge is vascular dissection. Using microsurgical techniques, SIEA and SIEV vessels can be divided, and anastomosis can be performed under a microscope to directly simulate free flap surgery. As shown by other reports, this technique is feasible in rats that have smaller caliber vessels ranging from 0.3 mm to 0.8 mm in diameter, but it requires using a microvascular surgery microscope or high magnification loupes [153–158]. To simplify this highly specialized procedure and make it more readily accessible to non-medical novice and less experienced researchers, anastomosis can be simulated by using atraumatic vascular clamps, as described in this protocol. Undoubtedly, the physical separation of epigastric vessels using a suture thread presents a risk of damaging the vessel with a needle. In our hands, the non-medical researcher who had used magnifying loupes to perform a physical vessel separation procedure by passing a suture and a needle between the SIEA and SIEV, experienced 20% attrition per 20 raised flaps.

Next, in BEFAF model, the survival/failure rate and efficacy of re-integration of right flap in response to the desired treatment regimen can be compared to that of saline-treated left flap control or vice versa. However, based on the results of our experiments using the methylene blue dye injection into the distal flap areas, the diffusion may impact the opposite side if the amount of injected solution exceeds 500 µL (in rats). It is also expected that the survival percent of left and right untreated flaps may slightly vary. Although this naturally occurring within-subject variation typically does not exceed 10% of total flap area, an excessive manipulation of flap pedicles or flap corners can introduce unwanted heterogenous effects. Moreover, a degree of hypoxia induced in the distal portions of the flaps may differ not only between two different strains of animals but also between the same-strain animals that are received from different vendors. For instance, we noticed that Lewis strain rats have relatively thin blood with less time needed for reperfusion, thus their flaps had a better chance of survival than the same type flaps created in SD strain rats. In turn, SD strain rats obtained from Charles River Laboratories were more susceptible to flap failure after the SI compared to SD rats received from Envigo. Thus, for purely procedural repeatability studies, all surgeries should be preferably performed using littermate animals obtained from the same vendor. Sex-, age- and diet-based differences should be considered as well: our preliminary data suggest that middle aged male rat flaps are more susceptible to IRI than female flaps, that young less obese rats heal quicker than elderly counterparts, and that while liquid versus solid chow diet does not have a significant impact on advancement flap survival per se, there are some changes in differential gene expression following the PI (manuscripts in preparation). Therefore, looking at combined versus isolated effects between fixed or random factors during the multifactorial statistical flap survival and molecular tissue response data analysis is recommended. Moreover, we noticed that not all rats produce similar amount of WE during the flap healing process, but the underlying causes of this phenomenon needs to be investigated in future studies.

Finally, in some experimental settings, rodents are exposed to repetitive anesthesia, which may induce respiratory failure and overdose-related death, especially in light weight animals. Susceptibility to overdose may also depend on animal gender: in our hands, female rats were less prone to overdosing than male rats. If the animal stops breathing during anesthesia, cardiopulmonary resuscitation is possible. Repetitive checking of animal vital signs, timely decrease, or cessation of anesthesia and sufficient time gaps between serial animal sedation events decreases fatal outcomes.

## Conclusions

Accurate comparisons between standardized complex wound healing studies would add to the translational knowledge base of complex tissue healing under normal and pathological conditions. BEFAFs may be used to investigate the spatiotemporal cellular and molecular responses to complex tissue injury and interventions simulating clinically relevant flap complications (e.g., secondary arterial, venous, or mixed ischemia) and therapeutic or surgical treatments (e.g., flap delay) in the presence or absence of confounding risk factors (e.g., substance abuse, irradiation, diabetes) or favorable wound-healing promoting activities (e.g., exercise). This technically challenging but feasible reconstructive surgery model eliminates inter-subject variability, while concomitantly minimizing the number of animals needed to achieve adequate statistical power. Overall flap experiments designed for studying the effects of SI may be lengthy, require good time management skills, visual aids, awareness, and stamina. They may appear too laborious for novice trainees but have the merit of simulating real-life clinical surgeries sometimes lasting more than 8 h. The described protocol of ischemic BEFAF model may serve as an aid for teaching basic vascular microsurgery techniques that focus on technical and non-technical skill acquisition in both medical and non-medical trainees.

### Supplementary Information

**Additional file 1.** Animal perioperative and post-operative record.

**Additional file 2: Figure S1.** Surgical tools used in flap raising surgery. **Figure S2.** A variation of BEFAF model using a quadruplicate biopsy design, biopsy excision and the temporary protection of wound bed after a partial excision of the tissue. **Figure S3.** Examples of vessel ligation, split SIEA isolation and reinforced pedicle clamping using two clamps (D). **Figure S4.** Principles of simple-interrupted suturing. **Figure S5.** Temporary sutures placement order and directions for the left-side BEFAF with real-time suturing order examples. **Figure S6.** Visual directions on how to pass a needle through the corners of left and right base of the BEFAF. **Figure S7.** Example of flap failure due to the development of spontaneous venous thrombosis. **Figure S8.** Principles of simple continuous running suturing at the end of the survival surgery. **Figure S9.** Animal recovering after major survival surgery. **Figure S10.** Preparation for digital planimetric analysis using commercial “Adobe Photoshop” software. **Figure S11.** Digital planimetric analysis using commercial “Adobe Photoshop” software. **Figure S12.** Examples of statistical simulated flap-study related data analysis. **Figure S13.** Non-acceptable and acceptable quality of raw unprocessed micrographs of Masson’s Trichrome-stained histological sections of rat’s BEFAF. **Figure S14.** Protein expression in rat's BEFAF wound exudate or PVA sponge fluid. **Figure S15.** Expected size distribution and concentration of extracellular vesicle (EV) subpopulations isolated from unperturbed abdominal rat skin.

**Additional file 3. **Examples of statistical flap data analysis and visualization.

## Data Availability

Data sharing is not applicable to this article as no datasets were generated or analyzed during the current study.

## Abbreviations

AO: Arterial occlusion
APF: Axial pattern flap
AVO: Arteriovenous occlusion
BEFAF: Bilateral epigastric fasciocutaneous advancement flap
D: Distal
DPBS: Dulbecco's Phosphate-Buffered Saline
EVs: Extracellular vesicles
FC: Flow cytometry
FV: Flap viability
FS: Flap survival
GF: Growth factor
GT: Granulation tissue
HDPE: High-density polyethylene
H&E: Hematoxylin and eosin (stain)
HIS: Histology
IB: Immunoblotting
IHC: Immunohistochemistry
LN_2_: Liquid nitrogen
M: Middle/medial
MT: Masson’s Trichrome (stain)
NC: Nucleic acid
P: Proximal
PBS: Phosphate-Buffered Saline
PI: Primary ischemia
POAMS: Post-operative animal monitoring sheet
POD: Postoperative day
PPE: Personal protective equipment
PVA: Polyvinyl alcohol
REP_1_: Primary reperfusion
REP_2_: Secondary reperfusion
RPF: Random pattern flap
SC: Subcutaneous
SD: Sprague–Dawley (rat strain)
SI: Secondary ischemia
SIEA: Superficial inferior epigastric artery
SIEV: Superficial inferior epigastric vein
siRNA: Small interfering ribonucleic acid
VO: Venous occlusion
WE: Wound exudate
