# Supplementary material for: Standardized Pre-clinical Surgical Animal Model Protocol to Investigate the Cellular and Molecular Mechanisms of Ischemic Flap Healing
Source: Biol Proced Online. 2024 Jan 17;26:2. doi: 10.1186/s12575-023-00227-w (PMC10792889; doi:10.1186/s12575-023-00227-w)
Supplement: Supplementary file 1 — Additional file 1. Animal perioperative and post-operative record. [file 12575_2023_227_MOESM1_ESM.pdf]

ANIMAL PERIOPERATIVE AND POST-OPERATIVE RECORD

Animal # (LABEL)		Initial Weight	
Date of Arrival		Protocol #	
Vendor		PI name	
Animal Sex (M/F)	☐ Female<br>☐ Male	Animal DOB and age	
Surgery Date		Animal Diet	☐ LIQUID CONTROL<br>☐ LIQUID TREATED<br>☐ REGULAR CHOW<br>☐ SPECIAL CHOW
Surgery begin:			
Surgery end:			
Analgetics administrated			
Time of administration		Experimental group	
Dose of analgetics		Right flap	Left flap
Saline dose			
Surgery Notes	Right flap	Left flap	
Imaging dates			
Biopsy dates			
Sacrifice date		Final outcome	

DAY 1 - Rat weight (grams) ______________________________ Food intake ________________________________

DATE		DATE	
1st check TIME/INITIALS		2nd check TIME/INITIALS	
Porphyrin deposits	☐ EYES<br>☐ NOSE	Porphyrin deposits	☐ EYES<br>☐ NOSE
Chewing	☐ TAIL<br>☐ RIGHT LEG<br>☐ LEFT LEG<br>☐ RIGHT FLAP<br>☐ LEFT FLAP	Chewing	☐ TAIL<br>☐ RIGHT LEG<br>☐ LEFT LEG<br>☐ RIGHT FLAP<br>☐ LEFT FLAP
Reluctance to move	☐ YES<br>☐ NO<br>☐ PARALYSIS	Reluctance to move	☐ YES<br>☐ NO<br>☐ PARALYSIS
Arched back	☐ YES<br>☐ NO	Arched back	☐ YES<br>☐ NO
Shaking	☐ YES<br>☐ NO	Shaking	☐ YES<br>☐ NO
Flap dehiscence	☐ NO<br>☐ YES, RIGHT SIDE<br>☐ YES, LEFT SIDE	Flap dehiscence	☐ NO<br>☐ YES, RIGHT SIDE<br>☐ YES, LEFT SIDE
Flap appearance	☐ CYANOSIS/ISCHEMIA<br>☐ NECROSIS	Flap appearance	☐ CYANOSIS/ISCHEMIA<br>☐ NECROSIS
Flap viability %	Left side | Right side	Flap viability %	Left side | Right side
Other notes			

DAY 2 - Rat weight (grams) _______________________________ Food intake: None Poor Good Excellent _____ ml

**DATE**			**DATE**		
**1st check TIME/INITIALS**			**2nd check TIME/INITIALS**		
**Porphyrin deposits**	☐ EYES<br>☐ NOSE		**Porphyrin deposits**	☐ EYES<br>☐ NOSE	
**Chewing**	☐ TAIL<br>☐ RIGHT LEG<br>☐ LEFT LEG<br>☐ RIGHT FLAP<br>☐ LEFT FLAP		**Chewing**	☐ TAIL<br>☐ RIGHT LEG<br>☐ LEFT LEG<br>☐ RIGHT FLAP<br>☐ LEFT FLAP	
**Reluctance to move**	☐ YES<br>☐ NO<br>☐ PARALYSIS		**Reluctance to move**	☐ YES<br>☐ NO<br>☐ PARALYSIS	
**Arched back**	☐ YES<br>☐ NO		**Arched back**	☐ YES<br>☐ NO	
**Shaking**	☐ YES<br>☐ NO		**Shaking**	☐ YES<br>☐ NO	
**Flap dehiscence**	☐ NO<br>☐ YES, RIGHT SIDE<br>☐ YES, LEFT SIDE		**Flap dehiscence**	☐ NO<br>☐ YES, RIGHT SIDE<br>☐ YES, LEFT SIDE	
**Flap appearance**	☐ CYANOSIS/ISCHEMIA<br>☐ NECROSIS		**Flap appearance**	☐ CYANOSIS/ISCHEMIA<br>☐ NECROSIS	
**Flap viability %**	**Left side**	**Right side**	**Flap viability %**	**Left side**	**Right side**
**Other notes**					

DAY 3 - Rat weight (grams) _______________________________ Food intake: None Poor Good Excellent _____ ml

**DATE**			**DATE**		
**1st check TIME/INITIALS**			**2nd check TIME/INITIALS**		
**Porphyrin deposits**	☐ EYES<br>☐ NOSE		**Porphyrin deposits**	☐ EYES<br>☐ NOSE	
**Chewing**	☐ TAIL<br>☐ RIGHT LEG<br>☐ LEFT LEG<br>☐ RIGHT FLAP<br>☐ LEFT FLAP		**Chewing**	☐ TAIL<br>☐ RIGHT LEG<br>☐ LEFT LEG<br>☐ RIGHT FLAP<br>☐ LEFT FLAP	
**Reluctance to move**	☐ YES<br>☐ NO<br>☐ PARALYSIS		**Reluctance to move**	☐ YES<br>☐ NO<br>☐ PARALYSIS	
**Arched back**	☐ YES<br>☐ NO		**Arched back**	☐ YES<br>☐ NO	
**Shaking**	☐ YES<br>☐ NO		**Shaking**	☐ YES<br>☐ NO	
**Flap dehiscence**	☐ NO<br>☐ YES, RIGHT SIDE<br>☐ YES, LEFT SIDE		**Flap dehiscence**	☐ NO<br>☐ YES, RIGHT SIDE<br>☐ YES, LEFT SIDE	
**Flap appearance**	☐ CYANOSIS/ISCHEMIA<br>☐ NECROSIS		**Flap appearance**	☐ CYANOSIS/ISCHEMIA<br>☐ NECROSIS	
**Flap viability %**	**Left side**	**Right side**	**Flap viability %**	**Left side**	**Right side**
**Other notes**					

DAY 4 - Rat weight (grams) ______________________________ Food intake: None Poor Good Excellent _____ ml

DATE		DATE	
1st check TIME/INITIALS		2nd check TIME/INITIALS	
Porphyrin deposits	☐ EYES<br>☐ NOSE	Porphyrin deposits	☐ EYES<br>☐ NOSE
Chewing	☐ TAIL<br>☐ RIGHT LEG<br>☐ LEFT LEG<br>☐ RIGHT FLAP<br>☐ LEFT FLAP	Chewing	☐ TAIL<br>☐ RIGHT LEG<br>☐ LEFT LEG<br>☐ RIGHT FLAP<br>☐ LEFT FLAP
Reluctance to move	☐ YES<br>☐ NO<br>☐ PARALYSIS	Reluctance to move	☐ YES<br>☐ NO<br>☐ PARALYSIS
Arched back	☐ YES<br>☐ NO	Arched back	☐ YES<br>☐ NO
Shaking	☐ YES<br>☐ NO	Shaking	☐ YES<br>☐ NO
Flap dehiscence	☐ NO<br>☐ YES, RIGHT SIDE<br>☐ YES, LEFT SIDE	Flap dehiscence	☐ NO<br>☐ YES, RIGHT SIDE<br>☐ YES, LEFT SIDE
Flap appearance	☐ CYANOSIS/ISCHEMIA<br>☐ NECROSIS	Flap appearance	☐ CYANOSIS/ISCHEMIA<br>☐ NECROSIS
Flap viability %	Left side / Right side	Flap viability %	Left side / Right side
Other notes			

DAY 5 - Rat weight (grams) ______________________________ Food intake: None Poor Good Excellent _____ ml

DATE		DATE	
1st check TIME/INITIALS		2nd check TIME/INITIALS	
Porphyrin deposits	☐ EYES<br>☐ NOSE	Porphyrin deposits	☐ EYES<br>☐ NOSE
Chewing	☐ TAIL<br>☐ RIGHT LEG<br>☐ LEFT LEG<br>☐ RIGHT FLAP<br>☐ LEFT FLAP	Chewing	☐ TAIL<br>☐ RIGHT LEG<br>☐ LEFT LEG<br>☐ RIGHT FLAP<br>☐ LEFT FLAP
Reluctance to move	☐ YES<br>☐ NO<br>☐ PARALYSIS	Reluctance to move	☐ YES<br>☐ NO<br>☐ PARALYSIS
Arched back	☐ YES<br>☐ NO	Arched back	☐ YES<br>☐ NO
Shaking	☐ YES<br>☐ NO	Shaking	☐ YES<br>☐ NO
Flap dehiscence	☐ NO<br>☐ YES, RIGHT SIDE<br>☐ YES, LEFT SIDE	Flap dehiscence	☐ NO<br>☐ YES, RIGHT SIDE<br>☐ YES, LEFT SIDE
Flap appearance	☐ CYANOSIS/ISCHEMIA<br>☐ NECROSIS	Flap appearance	☐ CYANOSIS/ISCHEMIA<br>☐ NECROSIS
Flap viability %	Left side / Right side	Flap viability %	Left side / Right side
Other notes			

DAY 6 - Rat weight (grams) ______________________________ Food intake: None Poor Good Excellent _____ ml

DATE		DATE	
1st check TIME/INITIALS		2nd check TIME/INITIALS	
Porphyrin deposits	☐ EYES<br>☐ NOSE	Porphyrin deposits	☐ EYES<br>☐ NOSE
Chewing	☐ TAIL<br>☐ RIGHT LEG<br>☐ LEFT LEG<br>☐ RIGHT FLAP<br>☐ LEFT FLAP	Chewing	☐ TAIL<br>☐ RIGHT LEG<br>☐ LEFT LEG<br>☐ RIGHT FLAP<br>☐ LEFT FLAP
Reluctance to move	☐ YES<br>☐ NO<br>☐ PARALYSIS	Reluctance to move	☐ YES<br>☐ NO<br>☐ PARALYSIS
Arched back	☐ YES<br>☐ NO	Arched back	☐ YES<br>☐ NO
Shaking	☐ YES<br>☐ NO	Shaking	☐ YES<br>☐ NO
Flap dehiscence	☐ NO<br>☐ YES, RIGHT SIDE<br>☐ YES, LEFT SIDE	Flap dehiscence	☐ NO<br>☐ YES, RIGHT SIDE<br>☐ YES, LEFT SIDE
Flap appearance	☐ CYANOSIS/ISCHEMIA<br>☐ NECROSIS	Flap appearance	☐ CYANOSIS/ISCHEMIA<br>☐ NECROSIS
Flap viability %	Left side / Right side	Flap viability %	Left side / Right side
Other notes			

DAY 7 - Rat weight (grams) ______________________________ Food intake: None Poor Good Excellent _____ ml

DATE		DATE	
1st check TIME/INITIALS		2nd check TIME/INITIALS	
Porphyrin deposits	☐ EYES<br>☐ NOSE	Porphyrin deposits	☐ EYES<br>☐ NOSE
Chewing	☐ TAIL<br>☐ RIGHT LEG<br>☐ LEFT LEG<br>☐ RIGHT FLAP<br>☐ LEFT FLAP	Chewing	☐ TAIL<br>☐ RIGHT LEG<br>☐ LEFT LEG<br>☐ RIGHT FLAP<br>☐ LEFT FLAP
Reluctance to move	☐ YES<br>☐ NO<br>☐ PARALYSIS	Reluctance to move	☐ YES<br>☐ NO<br>☐ PARALYSIS
Arched back	☐ YES<br>☐ NO	Arched back	☐ YES<br>☐ NO
Shaking	☐ YES<br>☐ NO	Shaking	☐ YES<br>☐ NO
Flap dehiscence	☐ NO<br>☐ YES, RIGHT SIDE<br>☐ YES, LEFT SIDE	Flap dehiscence	☐ NO<br>☐ YES, RIGHT SIDE<br>☐ YES, LEFT SIDE
Flap appearance	☐ CYANOSIS/ISCHEMIA<br>☐ NECROSIS	Flap appearance	☐ CYANOSIS/ISCHEMIA<br>☐ NECROSIS
Flap viability %	Left side / Right side	Flap viability %	Left side / Right side
Other notes			
