# Supplementary material for: Standardized Pre-clinical Surgical Animal Model Protocol to Investigate the Cellular and Molecular Mechanisms of Ischemic Flap Healing
Source: Biol Proced Online. 2024 Jan 17;26:2. doi: 10.1186/s12575-023-00227-w (PMC10792889; doi:10.1186/s12575-023-00227-w)
Supplement: Supplementary file 2 — Additional file 2: Figure S1. Surgical tools used in flap raising surgery. Figure S2. A variation of BEFAF model using a quadruplicate biopsy design, biopsy excision and the temporary protection of wound bed after a partial excision of the tissue. Figure S3. Examples of vessel ligation, split SIEA isolation and reinforced pedicle clamping using two clamps (D). Figure S4. Principles of simple-interrupted suturing. Figure S5. Temporary sutures placement order and directions for the left-side BEFAF with real-time suturing order examples. Figure S6. Visual directions on how to pass a needle through the corners of left and right base of the BEFAF. Figure S7. Example of flap failure due to the development of spontaneous venous thrombosis. Figure S8. Principles of simple continuous running suturing at the end of the survival surgery. Figure S9. Animal recovering after major survival surgery. Figure S10. Preparation for digital planimetric analysis using commercial “Adobe Photoshop” software. Figure S11. Digital planimetric analysis using commercial “Adobe Photoshop” software. Figure S12. Examples of statistical simulated flap-study related data analysis. Figure S13. Non-acceptable and acceptable quality of raw unprocessed micrographs of Masson’s Trichrome-stained histological sections of rat’s BEFAF. Figure S14. Protein expression in rat's BEFAF wound exudate or PVA sponge fluid. Figure S15. Expected size distribution and concentration of extracellular vesicle (EV) subpopulations isolated from unperturbed abdominal rat skin. [file 12575_2023_227_MOESM2_ESM.pdf]

ADDITIONAL FILE 2

1. OPTIONAL MATERIALS AND METHODS

1.1 Isolation of tissue-derived proteins

1. 100-500 mg of frozen flap tissue.
2. Protease inhibitor cocktail (e.g., Roche's Complete ULTRA Mini EasyPack Tablets by Sigma-Aldrich, # 05892970001).
3. Phosphatase inhibitor cocktail (e.g., Roche's PhosSTOP by Sigma-Aldrich, # 4906845001).
4. Whole tissue cell lysis (WCL) buffer to lyse tissue cells:
   - 150 mM NaCl,
   - 50 mM HEPES (pH 7.4),
   - 1 mM EGTA,
   - 1% Triton-X-100
   - 0.5% sodium deoxycholate (SOD),
   - 0.1% Sodium-dodecyl sulphate (SDS),
   - 10% glycerol,
   - 70 mM of n-Octyl-β-D-Glucoside (OG) (optional)
   - ddH2O.
5. Store at +4ºC. Immediately before use, supplement with protease and phosphatase inhibitors (e.g., 1 tablet per 10 mL of WCL buffer).
6. Flat stainless steel micro weighing spatulas. Example: https://tinyurl.com/mrybdmth
7. Cooling rack for cryogenic vials (e.g., Corning, #432052) – to prechill spatulas.
8. 500 ml volume capacity porcelain mortar and pestle. Keep at -20°C.
9. 10 ml Potter-Elvehjem borosilicate glass tissue grinder with PTFE pestle (Thomas Scientific, Swedesboro, NJ) – to homogenize tissue powder in WCL buffer. Keep at 4°C.
10. Overhead stirrer (e.g., Caframo BDC2002) – to attach and operate the PTFE pestle.
11. Rubber bucket for ice – to cool down Potter-Elvehjem borosilicate glass tissue grinders.
12. 250-500 mL glass beaker with ice – to stabilize tissue grinder while pouring the fine powder.
13. Analytical precision balance (e.g., 120g x 0.001g capacity).
14. Small disposable weighing dishes.
15. Liquid nitrogen (LN_2) supply and portable LN_2 dewar;
16. Thick Styrofoam box or another LN_2-proof container
17. 1.5 ml or 2 mL Eppendorf tubes.

18. Refrigerated tabletop centrifuge.
19. -80°C freezer – to store whole tissue lysates.

Using prechilled porcelain mortar and pestle, frozen tissue is crushed into a fine powder. Using prechilled spatula (LN_2 is poured in the Styrofoam container and the spatulas are placed in the cooling cryogenic rack) the powder is scooped and poured in 10 ml Potter-Elvehjem borosilicate glass tissue grinder containing 1 – 2 ml of WCL and kept in a glass beaker with ice. Powder is homogenized with 10-20 strokes using overhead stirrer set to 2000 rpm. The lysate is transferred into 1.5 ml or 2 ml Eppendorf tube, which is then placed in the rubber bucket on ice. Other samples are processed as necessary. At the end, the lysates are centrifuged at 10,000×g for 10 min at 4°C to remove insoluble material. The supernatants are transferred into a new set of tubes that are either stored at -80°C or immediately used to prepare the samples for SDS-PAGE or LDS-PAGE followed by a traditional or Multistrip Western Blotting as described previously [1].

1.2 Isolation of tissue-derived mRNA

For the isolation/purification of large and small RNA from flap biopsies, we use commercial phenol-free Animal Tissue RNA Purification Kit (Norgen, ON, Canada, #25700) supplemented with RNAse-Free DNAse I kit (Norgen, #25710). We follow the manufacturer’s recommendations but introduce minor adjustments. Briefly, ~150 mg of each frozen skin sample is crushed into fine powder under liquid nitrogen using mortar and pestle. The powder is collected into prechilled 10 mL size Potter Elvehjem tissue grinder containing 1 ml of ice-cold RL lysis buffer. The tissue is then homogenized using motorized overhead stirrer attached to the PTFE pestle, which is continuously wiped with RNAse AWAY reagent surface decontaminant (Thermo Fisher Scientific, #7003) before and after processing of each sample. The homogenate is then diluted with 850 µL of nuclease-free molecular biology grade water, transferred into 2 mL DNA low-binding Eppendorf tubes and treated with 75 µL RNAse-free 20 mg/ml proteinase K aqueous solution at 55°C dry-heat bath for 30 min. Proteinase K aids in the removal of the various proteins present in fiber-rich tissues including collagen, contractile proteins, and connective tissues. The digested sample is then centrifuged at 14,000×g for 2 min to filter out insoluble debris. To reduce viscosity, the supernatant of each sample is passed through the individual disposable QIAshredder spin-columns (Qiagen, #79656). The filtrate is mixed with 100% ethanol at 1:1 vol/vol ratio and subjected to on column RNA binding, on-column DNAse treatment, column wash and RNA elution steps. The nucleic acid (NC) concentration, purity ratio and quality are measured by NanoDrop spectrophotometer and Qubit 4 fluorometer in triplicate (Qubit RNA IQ Assay, Thermo Fisher Scientific). The NanoDrop requires just 1 µL of sample and by simply measuring at 230, 260 and 280 nm, one can

obtain the total amount of NC present (260 nm), any protein contamination (280 nm) and any phenol or other solvent contaminants (230 nm) present in the sample. The Qubit RNA XR (extended range) and HS (high-sensitivity) assay dyes that selectively bind only to intact RNA can also be used to quantitate RNA. NC concentrations are measured using the fluorescence signal of the sample and a calibration curve that is generated from standard samples of known concentration and fit to appropriate regression models. Alternatively, RNA integrity is measured by Agilent 2100 bioanalyzer (Agilent Technologies Inc, PaloAlto, CA).

1.3 Isolation of fasciocutaneous tissue-derived extracellular vesicles (EVs)

1. 1000-2000 mg of freshly excised flap tissue.
2. Refrigerated tabletop centrifuge – for sample pre-clearance and isolation of large-size EVs.
3. Ultracentrifuge with applicable rotor – for isolation of medium and small-size EVs.
4. Humidified 37°C and 5% CO_2 incubator.
5. 0.5 ml, 2 ml, 15 ml, and 50 mL Eppendorf Protein LoBind centrifuge tubes.
6. 36 ml polyallomer ultracentrifuge tubes.
7. Single-channel pipettes and ultra-low adhesion pipette tips.
8. 5 ml, 10 ml, and 25 ml serological glass (recommended) or plastic pipettes.
9. 70 µm cell strainers (Corning, #431751) and 40 µm cell strainers (Corning, # 431750).
10. Disposable sterile 50 ml tube top vacuum filter system with cellulose acetate 0.45 µm filters (Corning #430314 or CELLTREAT Scientific Products, #229709).
11. 0.45 µm pore size cellulose acetate micro-centrifugal filters (e.g., Thermo Scientific, #F25172).
12. Razor blades.
13. Sterile 100x20 mm tissue culture dishes – for cutting the tissue into fine pieces.
14. Liberase TM Research grade (Roche) (0.5 mg/ml final concentration in HBSS) – for dissociation of skin tissue into single-cell suspension and release of EVs. Alternatively, use a mixture of Collagenase IV (1000 U/ml) and dispase II (2.5 U/ml) or Collagenase I alone (20 mg/ml).
15. 0.1 mg/ml DNase I (Sigma-Aldrich) (optional).
16. Tube revolver/rotator with speed adjustment and rotisseries/paddles fit for 15 ml or 50 ml tubes (e.g., Thermo Scientific, #88881001) – used at 25 rpm, 37°C and 5% CO_2 to facilitate tissue digestion.
17. 100 mM RNAse-free EDTA solution (used at 1 mM final concentration) – to stop digestive reaction.
18. 1X HyClone™ 0.1 µM filter-sterilized PBS without calcium and magnesium (pH 7.0 – 7.2) (Cytiva, #SH30256.01).

19. Tissue EV isolation buffer (TEIB): 250 mM D-Sucrose; 10 mM triethanolamine (TEA) in 1X PBS. Sterile 0.22 µm filter and store at 4°C. Alternatively, use 0.22 µm-filtered sterile RPMI without FBS.
20. Primary EV resuspension buffer (PBS-TR): 1X PBS supplemented with 25 mM of ≥ 99% pure D(+)-Trehalose anhydrous (Chem-Impex Int., Wood Dale, IL, Cat # 31116), and then filtered through the 25 mm hydrophilic 0.05 µm pore-size PES syringe filter with 1.0 µm GF pre-filter (Tisch Scientific, Cleves, OH, Cat # SF18256)). D-Trehalose was shown to prevent EV aggregation during their short-term storage at 4°C or cryodamage during long-term storage at -80°C [2].
21. Tabletop refrigerated centrifuge
22. Ultracentrifuge
23. Nanoparticle analyzer (e.g., NanoSight NS300 (Malvern Panalytical, Malvern, UK) and/or Anton-Paar Litesizer 500) – to analyze isolated EV size distribution and/or concentration.

Due to sample processing variability, it is recommended to isolate EVs using at least 3 technical replicate (TR) samples in parallel. Tissues are washed from blood in PBS, weighted, cut into small ~5 mm pieces, and placed into 15 ml Protein LoBind tubes containing 10 ml of enzymatic digestion solution with/without DNAse I. Tissues are incubated for 2 h in tube revolver/rotator at 25 rpm, 37°C and 5% CO_2. To stop enzymatic reaction, 100 µL of 100 mM EDTA is added to reach 1 mM final concentration. Each digestate is passed through 70 µm cell strainer, centrifuged at 400×g for 10 min at 4°C, and thereafter passed through 40 µm cell strainer, followed by centrifugation at 800×g for 10 min at 4°C and addition of TEIB to a final 40 mL volume and further centrifugation at 2,000×g for 30 min at 4°C. Removal of larger particles by vacuum-assisted filtration using 0.45 µm cellulose acetate filters is optional but strongly recommended. After this step, the filtrates may be stored at 4°C for a week or processed immediately. Freezing is not recommended, especially if EVs are to be analyzed for the metabolic processes. For the isolation of larger size EVs (microvesicles, MV), 34 ml of solution is centrifuged at 14,000×g for 1 h at 4°C. 33 ml of supernatant (SN) is transferred to a new set of 36 ml polyallomer tubes, and the remaining solution is discarded. The tubes are inverted to remove residual fluid for 3 min, wiped gently, and the resulting pellet (PT) at the bottom of the tube is resuspended in 250 µL (for 1000 mg wet weight tissue sample) to 500 µL (for 2000 initial wet weight sample) of PBS-TR. The volume of PBS-TR may be adjusted to yield a more or less concentrated primary analyte (PA). The resulting PT suspension is marked as MV-MIX. It is then centrifuged at 2,500×g for 10 min at 4°C to remove larger insoluble aggregates. 200 µL or 450 µL of SN is transferred to a new tube. The resulting PA is labeled as MV-2.5K-SN-F (if the digestate was filtered) or as MV-2,5K-SN (if the digestate weas not filtered). Smaller-size EVs (exosomes, EXO) are isolated from 34 ml of transferred SN at 120,000×g for 1 h at 4°C. After removal of main and residual fluids, the PT is resuspended in 125 µL (for 1000 mg wet weight tissue sample) or 225 µL (for 2000 initial wet weight sample) of PBS-

TR. The resulting PT suspension is marked as EXO-MIX. It may then be centrifuged at 10,000×g for 10 min at 4°C to remove larger insoluble aggregates. 100 µL or 200 µL of SN is transferred to a new tube. The resulting PA is labeled as EXO-10K-SN-F (if the digestate was filtered) or as EXO-10K-SN (if the digestate was not filtered). A small amount of PA is then diluted in PBS to yield a final analyte (FA) with an optimal particle density for downstream DLS and/or NTA analysis using manufacturer's recommendations. In our hands, both skin-derived MV and EXO particles could be detected at camera level set to 14 in 2 ml samples that were diluted 100 or 3200 times, respectively, and moving across the field of view at arbitrary syringe pump speed (SPS) set to 35 (See **Figure S13**). If the EV solution is further used to extract and analyze cargo via immunoblotting, it should be supplemented with protease/phosphatase inhibitors and immediately lyzed prior to long-term lysate storage at -80°C.

1.4 PVA sponge implantation and processing to extract wound exudate material

1. Sterile Ivalon PVA Ear Wick 9 mm x 15 mm (e.g., Fabco, #Q605201)
2. Sterile 100x20 mm Tissue culture dish – to place, soak and dissect the sponges.
3. Sponge soaking buffer: sterile 1X PBS.
4. Disposable sterile scalpel (e.g., Fisher Scientific, #02-688-78) – for cutting the PVA sponges.
5. Flat-tip forceps to squeeze the sponges and release WE.
6. PVA sponge lysis buffer for protein solubilization: prepare 100 mL of 50 mM HEPES, pH 7.5; 150 mM NaCl; and 1% Triton X-100. Keep at 4°C for up to 6 months. Supplement with EDTA-free protease inhibitors right before use.
7. 0.45 µm pore size cellulose acetate micro-centrifugal filters (e.g., Thermo Scientific, # F25172).

Pour soaking buffer in a sterile 100x20 mm tissue culture dish. Soak each sponge and dissect an enlarged piece into 3 pieces.

2. SUPPLEMENTAL FIGURES

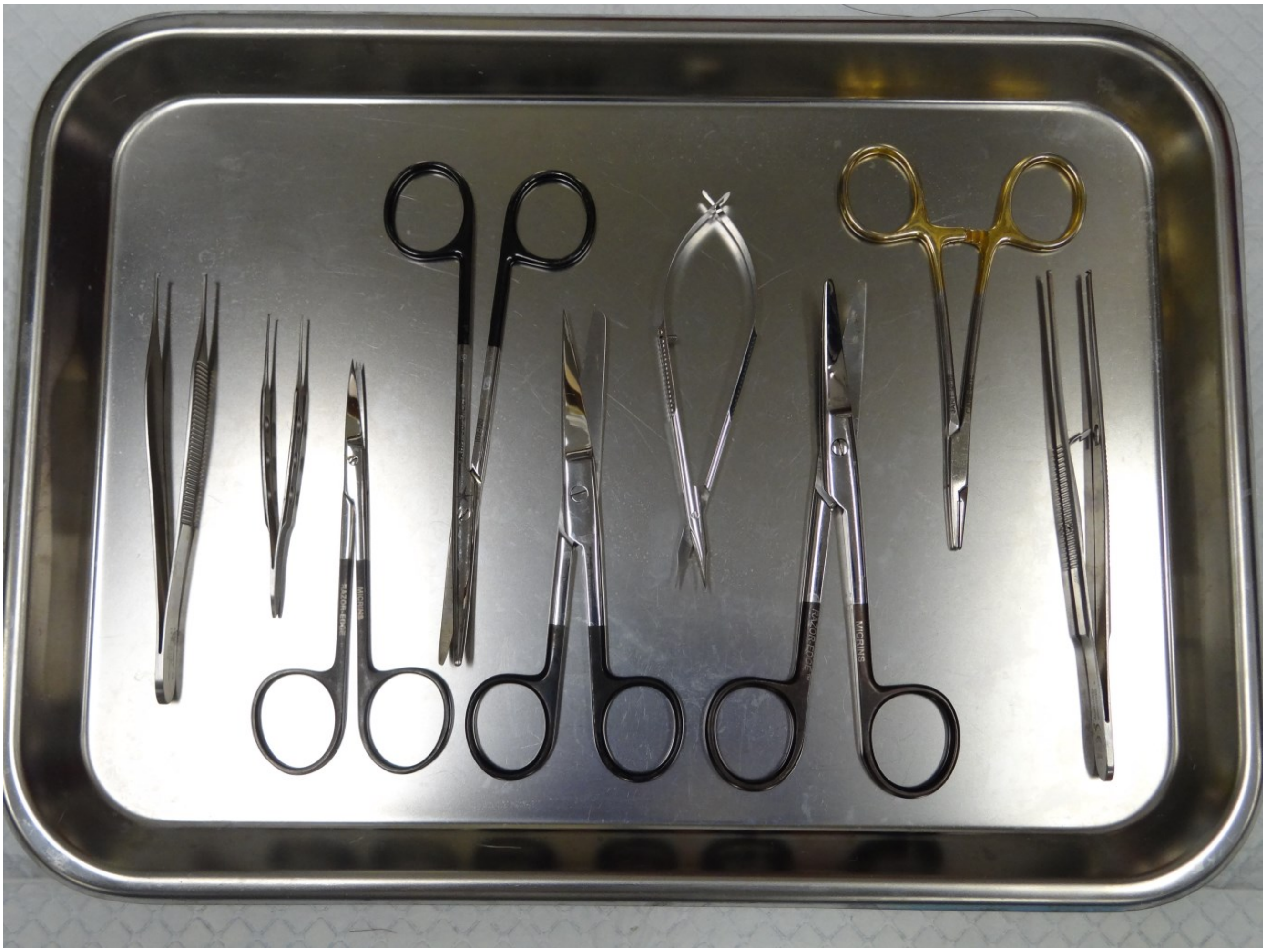

Figure S1. Surgical tools used in flap raising surgery. From left to right: Adson tissue forceps, Micro/fine forceps, Iris Supercut scissors, Mayo dissecting scissors (curved), Metzenbaum-Nelson scissors (straight), Noyes Spring Scissors, Micrins razor-edge dissecting scissors (straight), Halsey Micro Needle Holder, Vascular Clamp Applying Forceps.

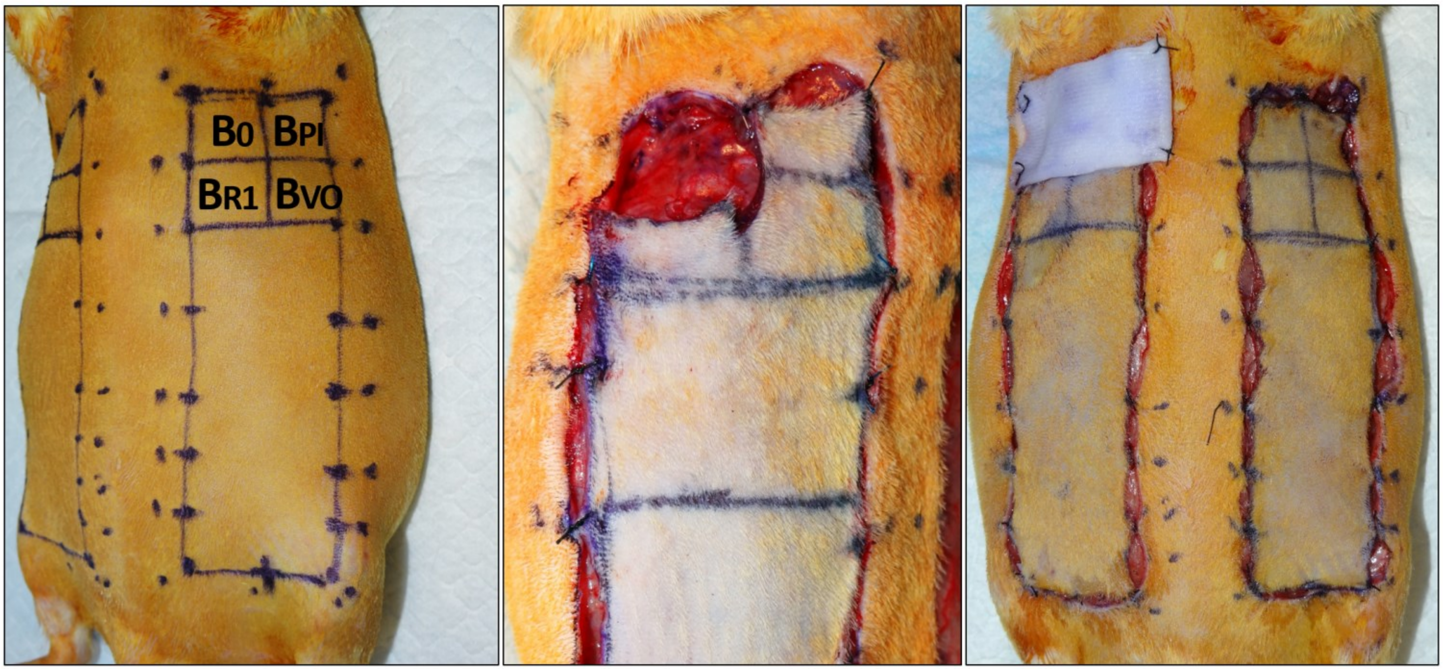

Figure S2. A variation of BEFAF model using a quadruplicate biopsy design (left panel), biopsy excision (middle panel) and the temporary protection of wound bed after a partial excision of the tissue (right panel).

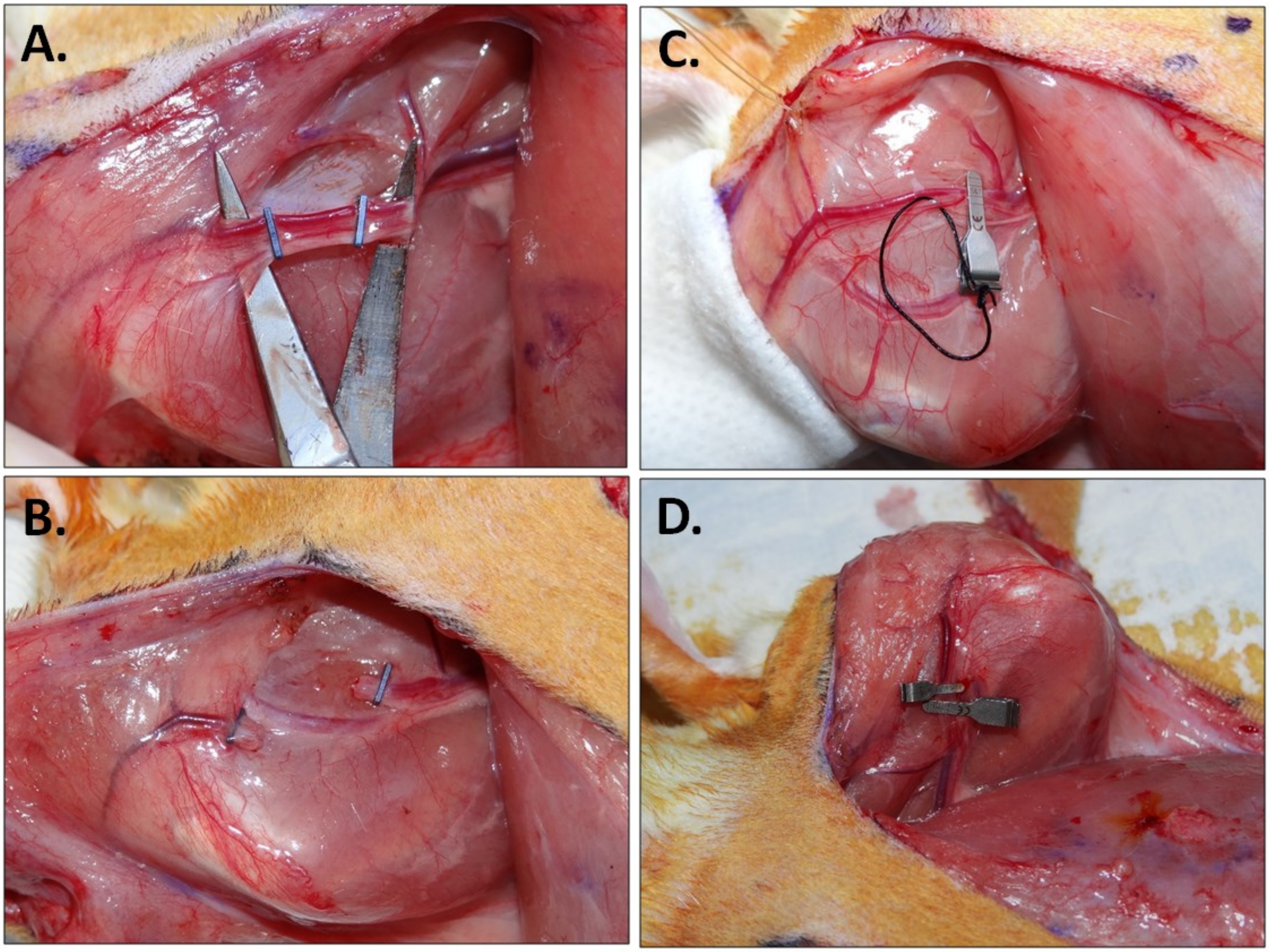

Figure S3. Examples of vessel ligation (A, B), split SIEA isolation (C) and reinforced pedicle clamping using two clamps (D).

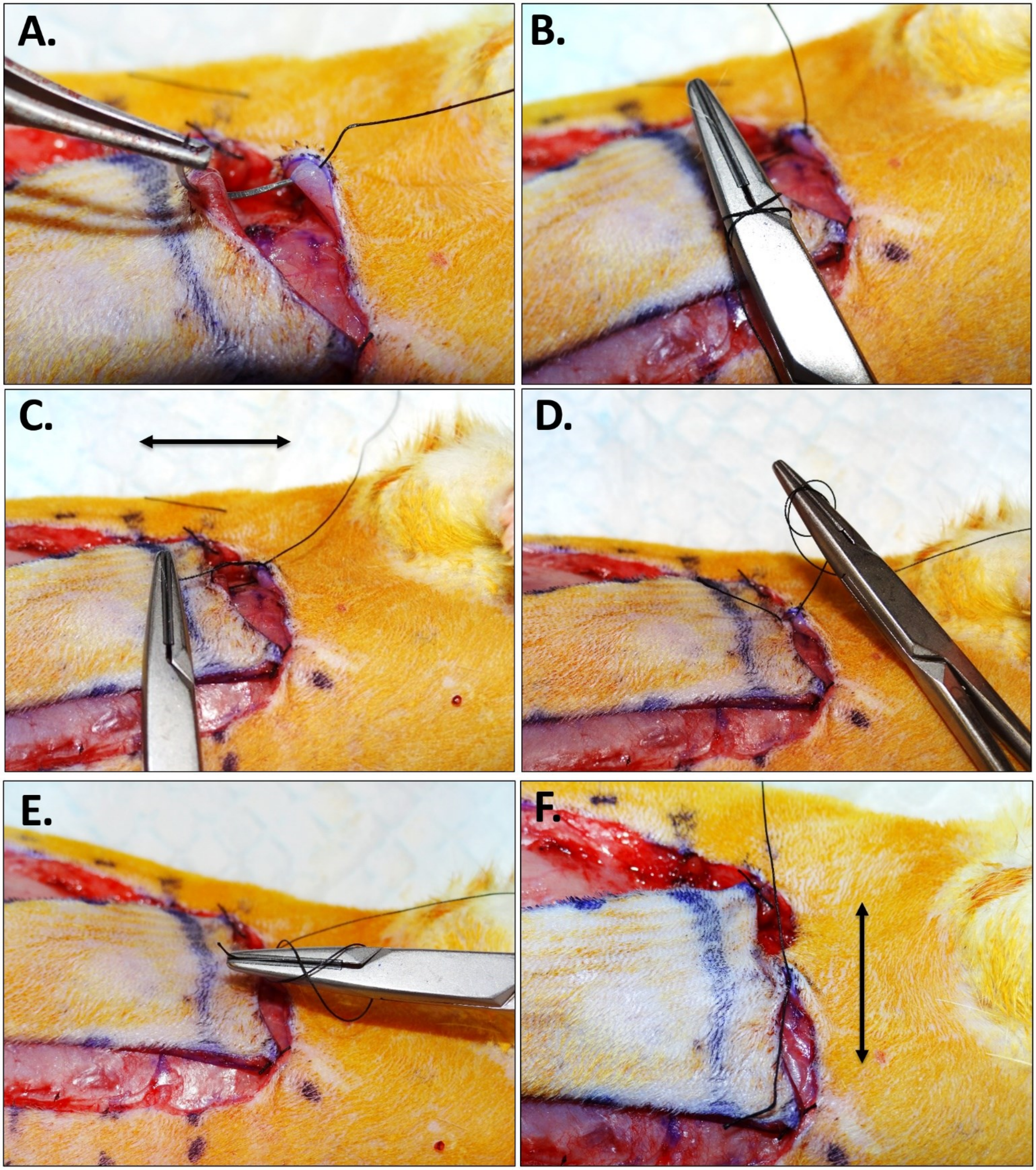

Figure S4. Principles of simple-interrupted suturing. A. Pass a needle through the inner tissue to avoid piercing the skin. **B.** Throw a couple of loops onto the tip of needle holding scissors and grab an end of the suture to pass it through these loops**. C.** Close the knot in the direction shown**. D.** Make two additional loops on the scissors**. E-F.** Grab the end of a suture and close the knot in the direction shown. Cut the sutures, leaving short ends at the base of the knot**.**

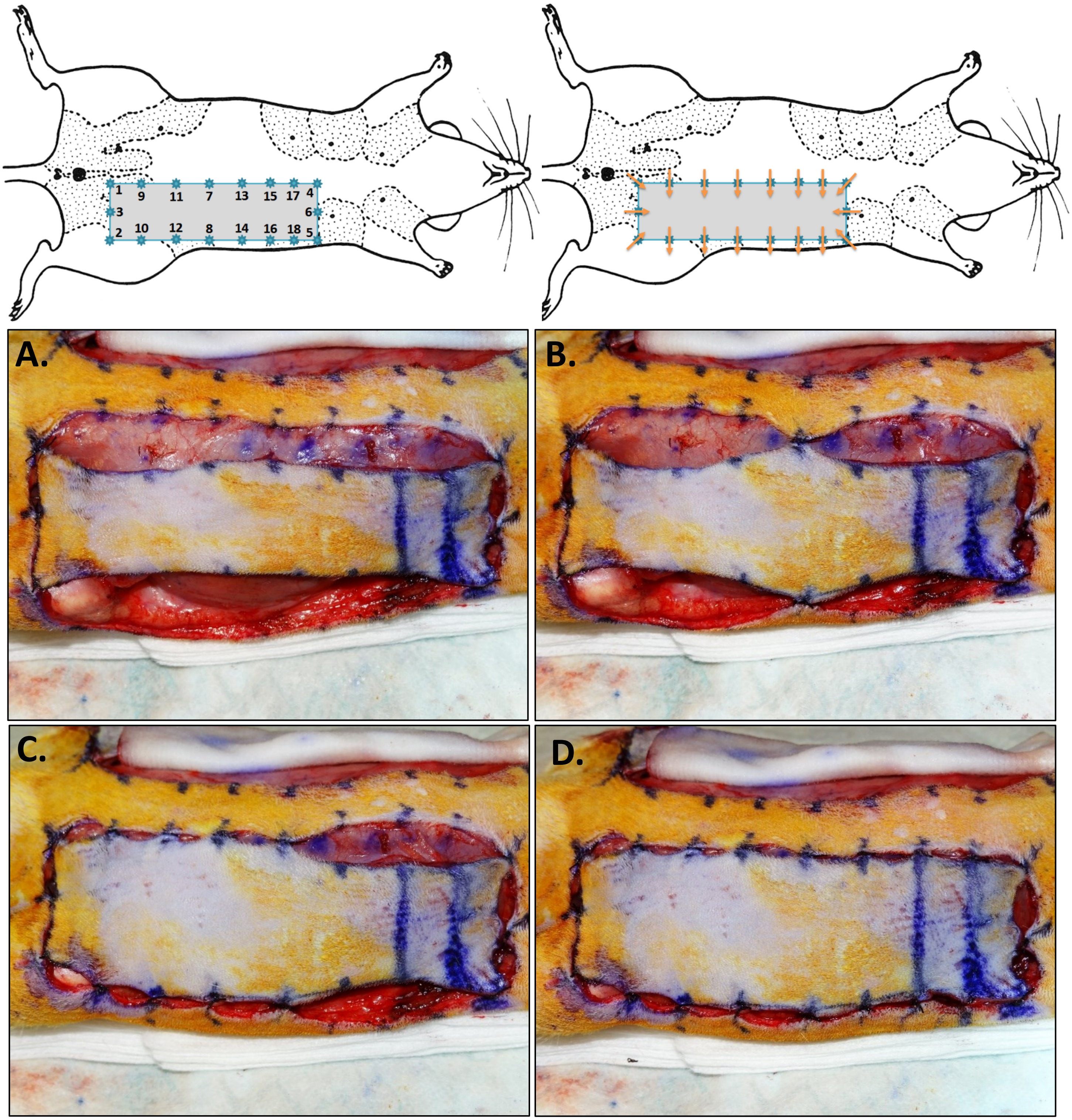

Figure S5. Temporary sutures placement order (upper left panel) and directions (upper right panel) for the left-side BEFAF with real-time suturing order examples (bottom panel) covering suture placement at points 1-6 (A), 7-8 (B), 9-12 (C) and remaining 13-18 (D). Multiple suture points help to achieve even distribution of flap tension. Horizontal lines at the end of BEFAF indicate future biopsy excision areas.

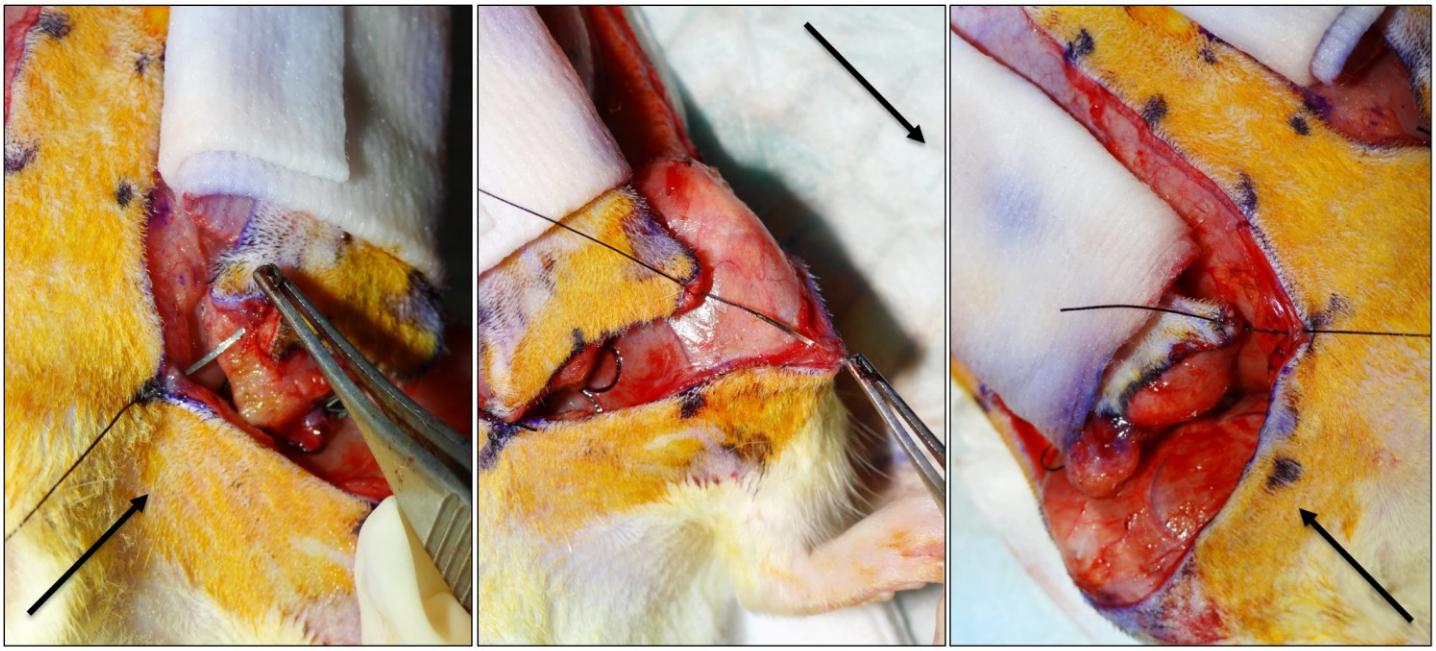

Figure S6. Visual directions on how to pass a needle through the corners of left (left and middle panel) and right (right panel) base of the BEFAF. The suturing directions (represented by black arrows) for the remaining corner of the right flap (not shown) are identical to that indicated in the left image.

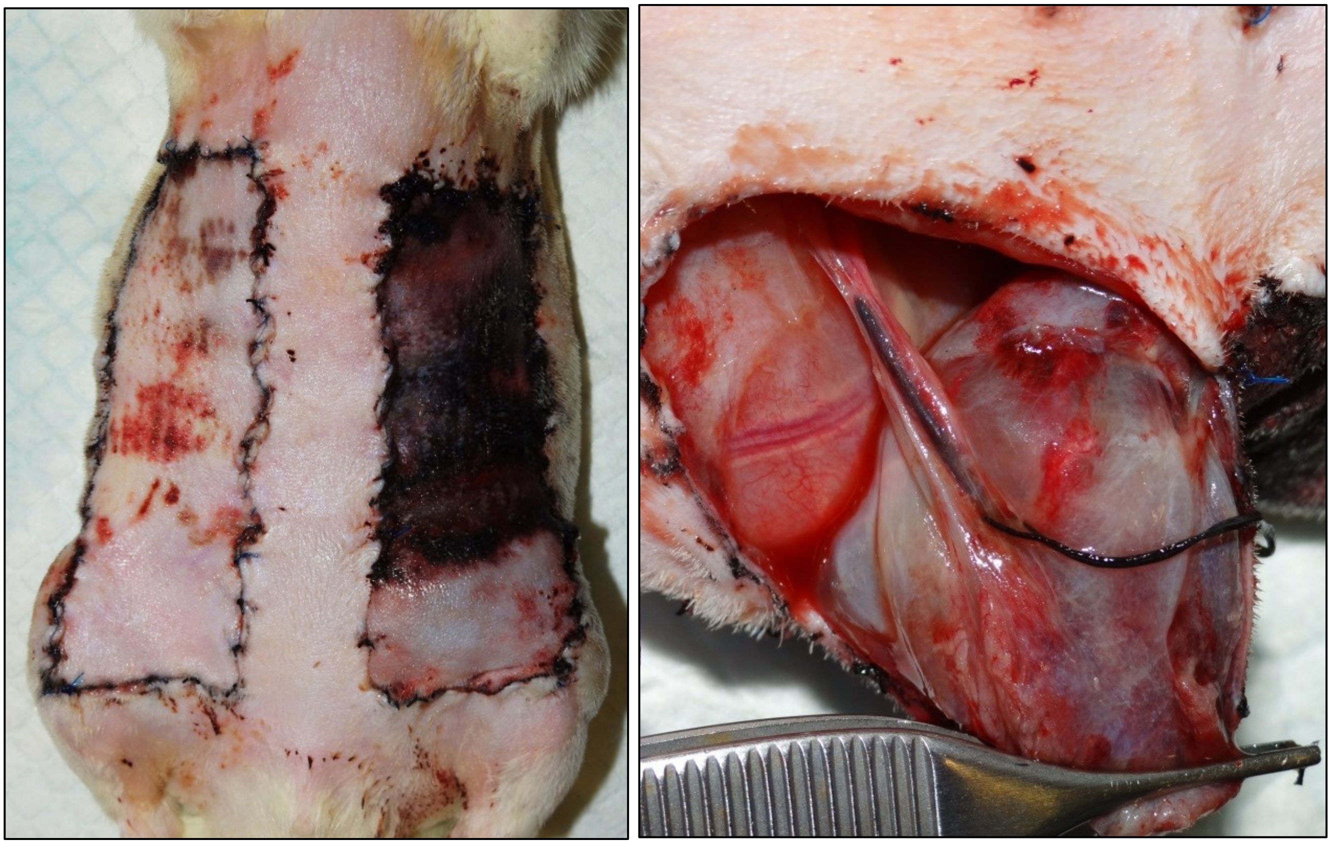

Figure S7. A photograph of male SD rat who's left BEFAF did not recover from the 4 h of PI after 2 h of REP_1 due to the development of spontaneous venous thrombosis, which was confirmed by close inspection of the pedicle (left panel) before any attempt to salvage a flap.

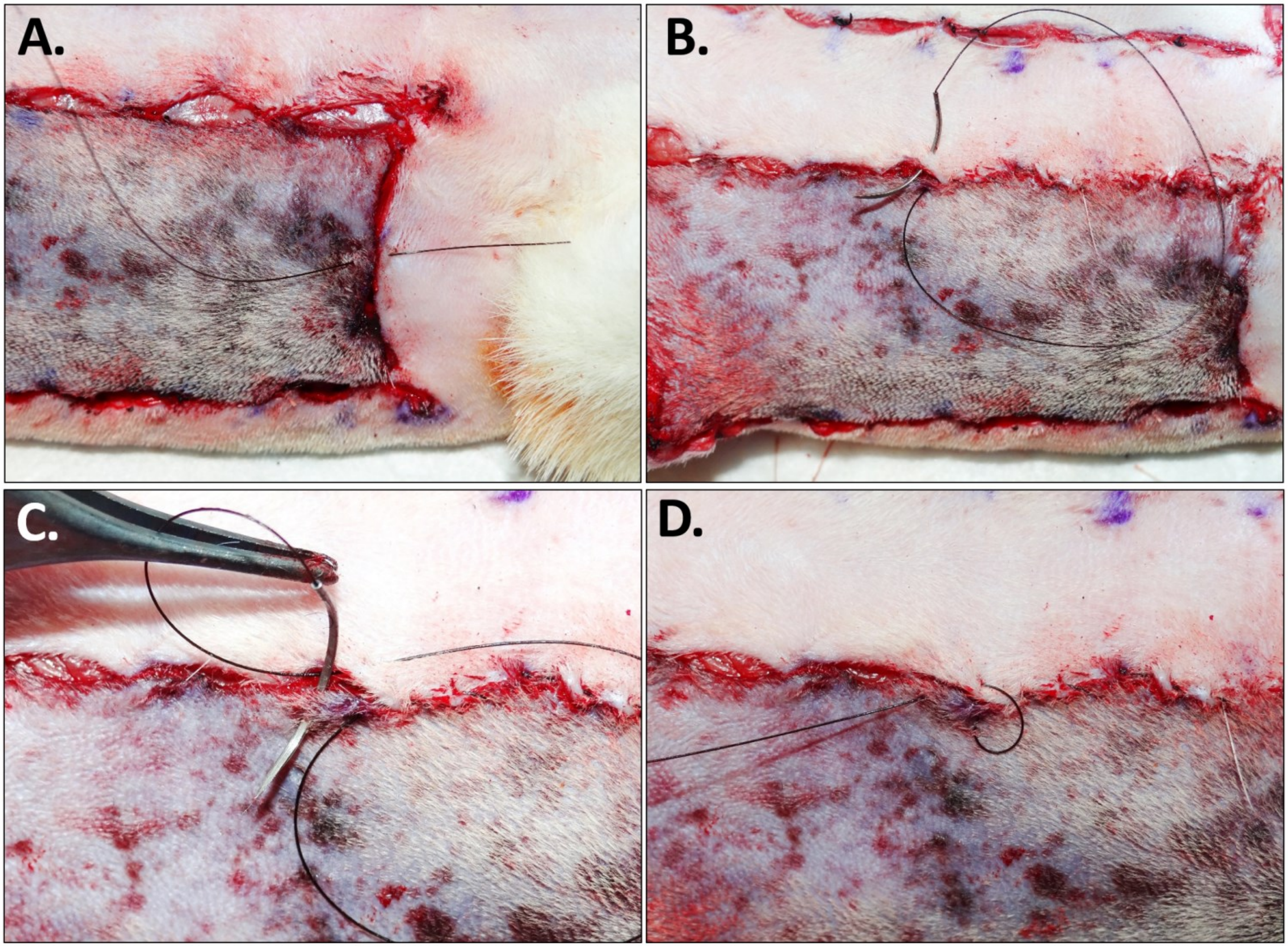

Figure S8. Principles of simple continuous running suturing at the end of the survival surgery.

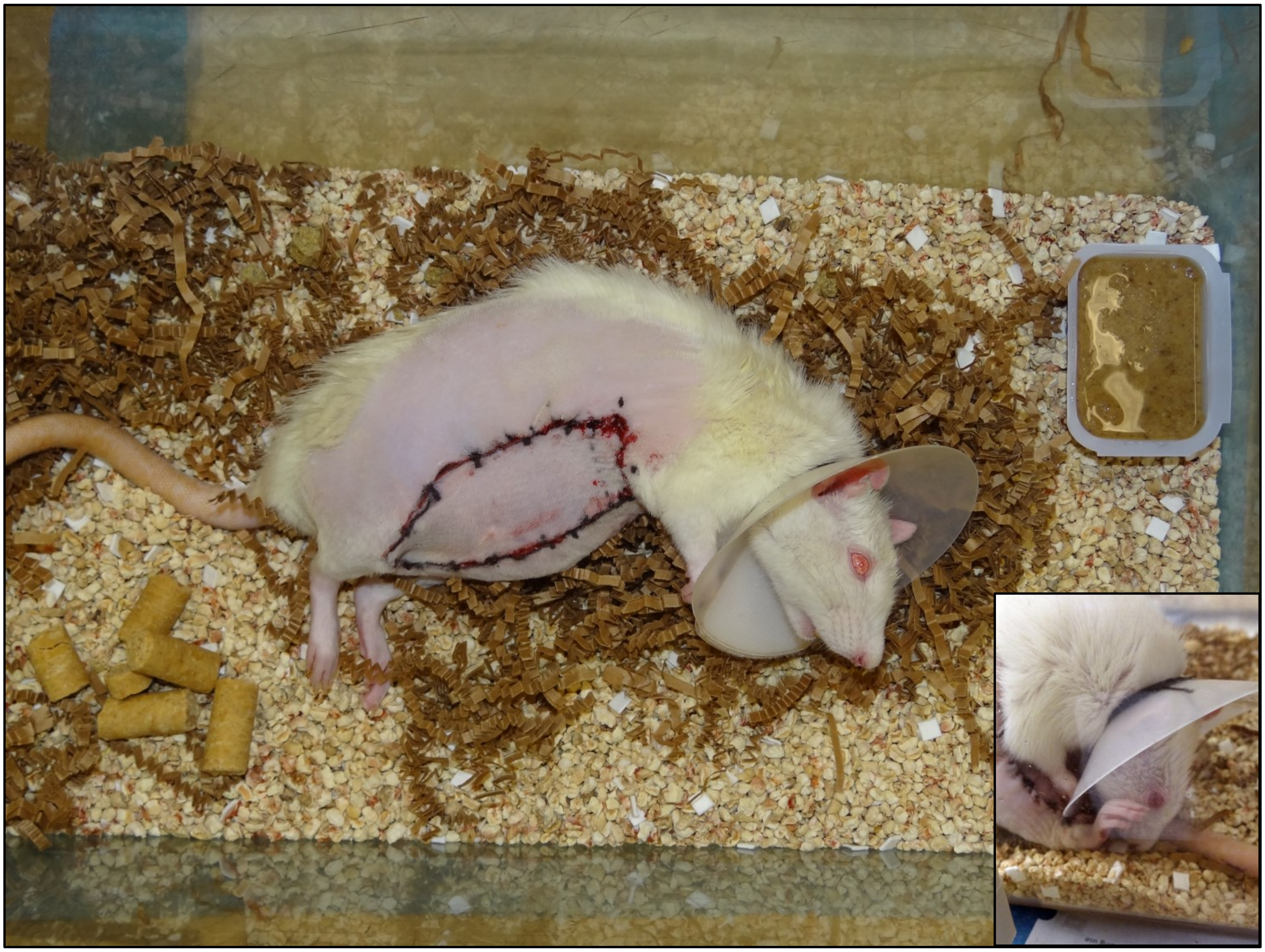

Figure S9. Animal recovering after major survival surgery in the cage supplied by water, regular chow diet, Nutra-Gel Diet™ diet and bacon softies. Once the anesthesia wears off, the animal resumes regular activities. Despite the placement of protective collar, leaner female rats may start picking up on sutures (inlet image).

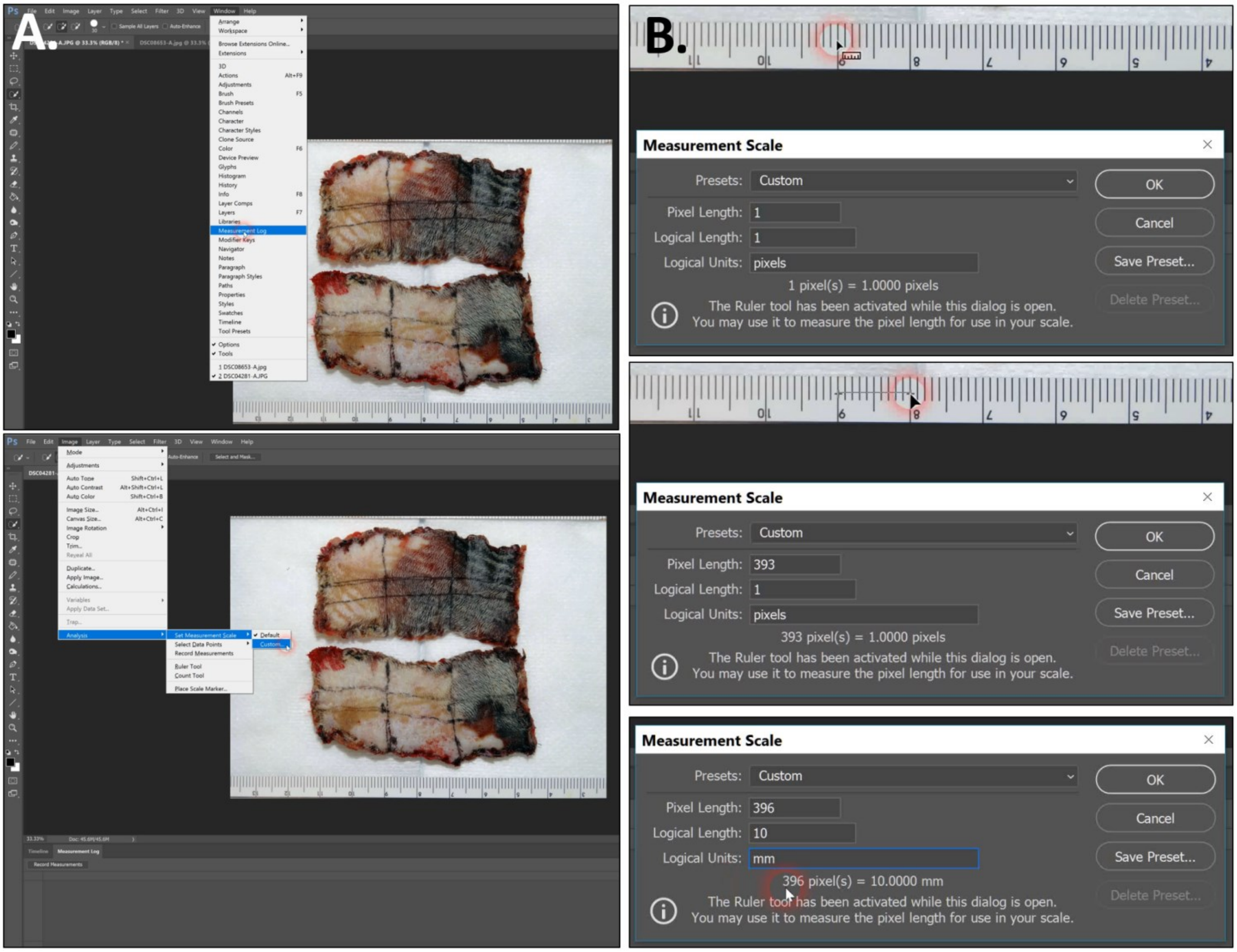

Figure S10. Preparation for digital planimetric analysis using commercial "Adobe Photoshop" software. A. Upper panel. Open a digital photo of one or both flaps placed next to a precision ruler and activate "Measurement Log" panel, which is under "Window" menu. **Bottom panel.** Set a custom measurement scale in millimeters. Open "Image" menu, "Analysis" panel and "Set Measurement Scale" subpanel. Then click on "Custom". **B.** A digital Ruler tool should automatically appear along with an opened custom "Measurement Scale" panel. Place the origin point of the Ruler tool on the precision scale (upper panel) and drag it while holding "Shift" button as a straight line for 10 mm (middle panel). After stopping, enter the value "10" and "mm" in the input tabs "Logical Length" and "Logical Units", respectively. Click OK.

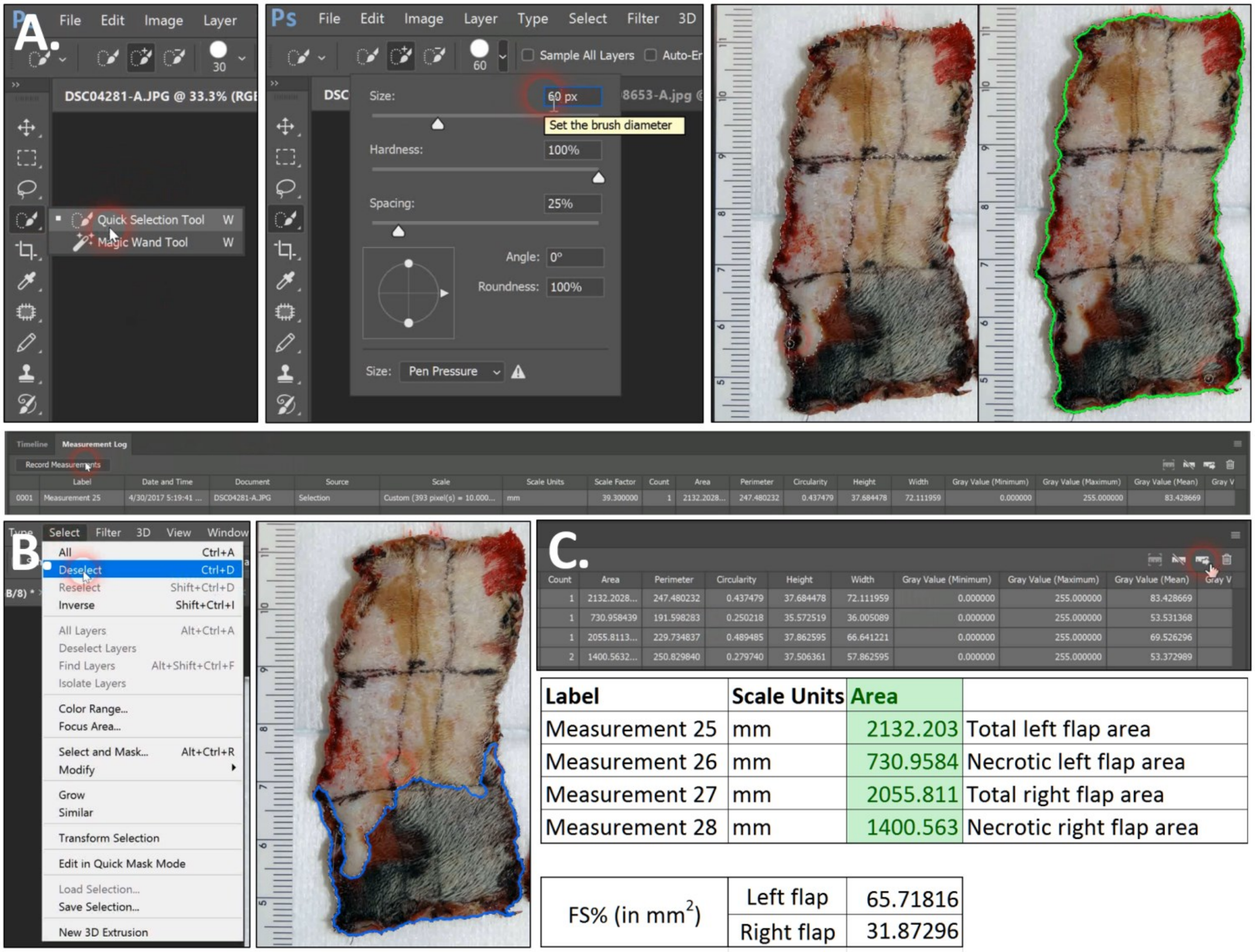

Figure S11. Digital planimetric analysis using commercial "Adobe Photoshop" software. A. Estimation of total flap area. Using "Quick Selection" tool located in the left toolbar **(upper left panel)** and a fine "60px" diameter brush (**upper middle panel**), select the total area of the flap (**upper right panels**). Delete the unwanted selected regions by holding "Alt" button. Flap margins are shown by bright green lines. Once all flap margins are included in the selection, click on the "Record Measurements" button in the "Measurement Log" window. Once the values appear in the window **(bottom panel**), proceed to the calculation of either necrotic or viable flap area. **B**. **Gross estimation of necrotic flap area**. Deselect previous selection of the flap by clicking on "Deselect" panel under "Select" window (**left panel**). Using "Quick Selection" tool and a fine "30px" diameter brush, select visibly necrotic flap areas (**right panel**). Delete the unwanted selected regions by holding "Alt" button. Click on the "Record Measurements" button in the "Measurement Log" window. Repeat the same steps for another flap. **C. Export of data.** Recorded measurements can be exported as .txt file **(upper panel**) to the chosen destination and then opened in MS Office Excel program as a datasheet (**bottom panel**). Unwanted data fields may be hidden. The percentage of flap survival (FS%) is calculated as the total flap area minus the necrotic flap area divided by the total flap area (in square millimeters) multiplied by 100.

	Left	Right
Rat 1	57	25
Rat 2	65	32
Rat 3	71	47
Rat 4	86	50
Rat 5	60	34
Rat 6	74	44
Rat 7	80	37
Rat 8	90	55

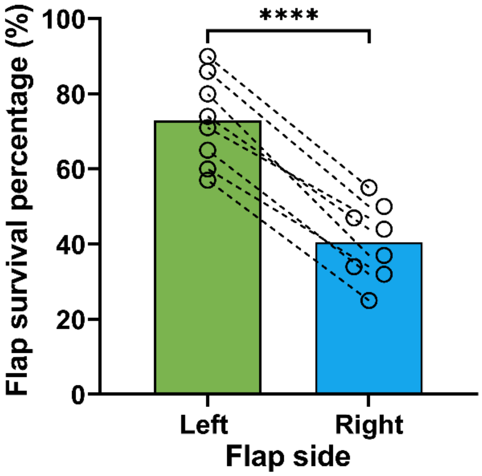

	Baseline	PI	REP1	SI
Male 1	21	55	180	162
Male 2	27	49	174	170
Male 3	23	52	190	179
Male 4	29	42	210	200
Female 1	31	50	211	185
Female 2	30	44	200	190
Female 3	28	36	197	180
Female 4	30	40	205	184

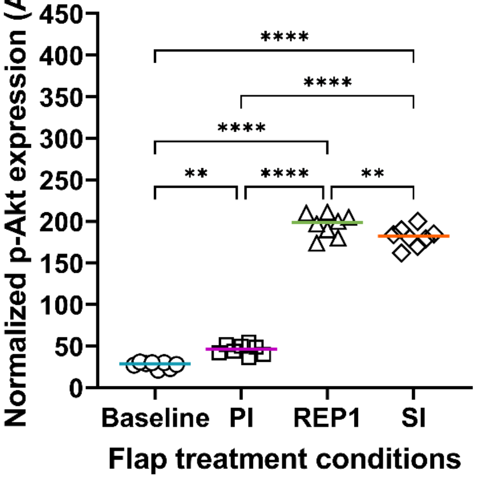

	Female					Male				
	S1	S2	S3	S4	S5	S1	S2	S3	S4	S5
Baseline	20	25	21	27	23	29	31	30	28	30
PI	50	47	55	49	52	60	64	65	62	66
REP1	70	68	72	74	67	222	211	200	197	205
SI	80	86	90	92	84	185	178	164	180	170

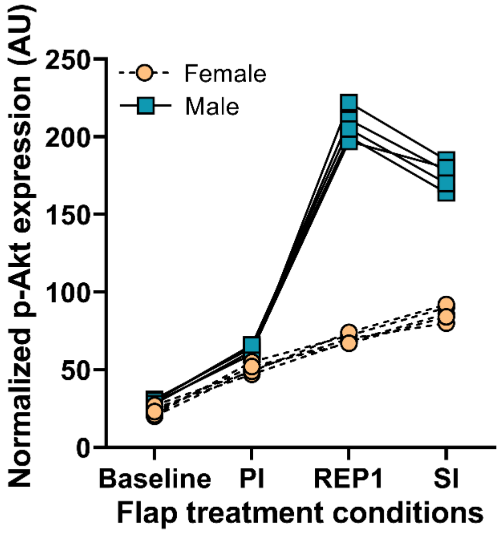

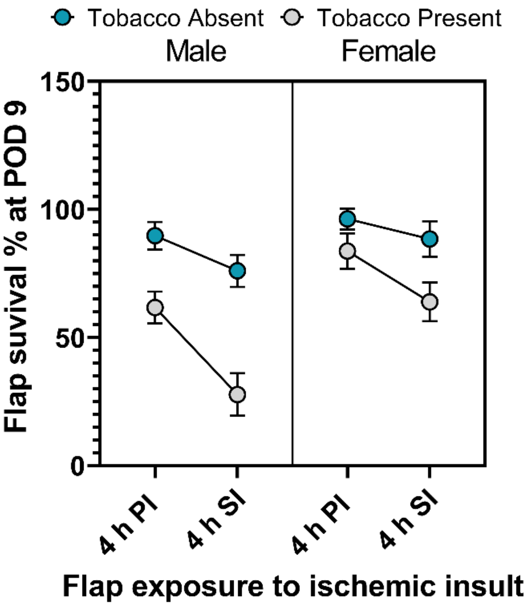

	Male Tobacco Absent							Male Tobacco Present						
	S1	S2	S3	S4	S5	S6	S7	S1	S2	S3	S4	S5	S6	S7
4 h PI	90	95	85	92	97	82	87	60.0	65.0	55.0	68.0	60.0	54.0	70.0
4 h SI	70	84	76	72	80	68	82	28.0	16.0	35.0	40.0	25.0	20.0	30.0

	Female Tobacco Absent							Female Tobacco Present						
	S1	S2	S3	S4	S5	S6	S7	S1	S2	S3	S4	S5	S6	S7
4 h PI	100	90	100	95	97	92	100	90	80	85	85	90	70	86
4 h SI	100	80	85	88	94	82	90	68	62	75	70	61	58	53

Figure S12. Examples of statistical simulated flap-study related data analysis. Upper left panel. Example1: paired T-test of left and right fap survival of multiple subjects (N = 8). **Upper right panel.** Example 2: comparison of flap conditions independent of animal gender by RM-ANOVA (N = 8). **Bottom left panel.** Example 3: comparison of gender-dependent flap response/signals by 2-way RM-ANOVA (N = 5 per each group). **Bottom right panel.** Example 4: comparison of gender-dependent flap response/signals by 3-way ANOVA (N = 7 per each group).

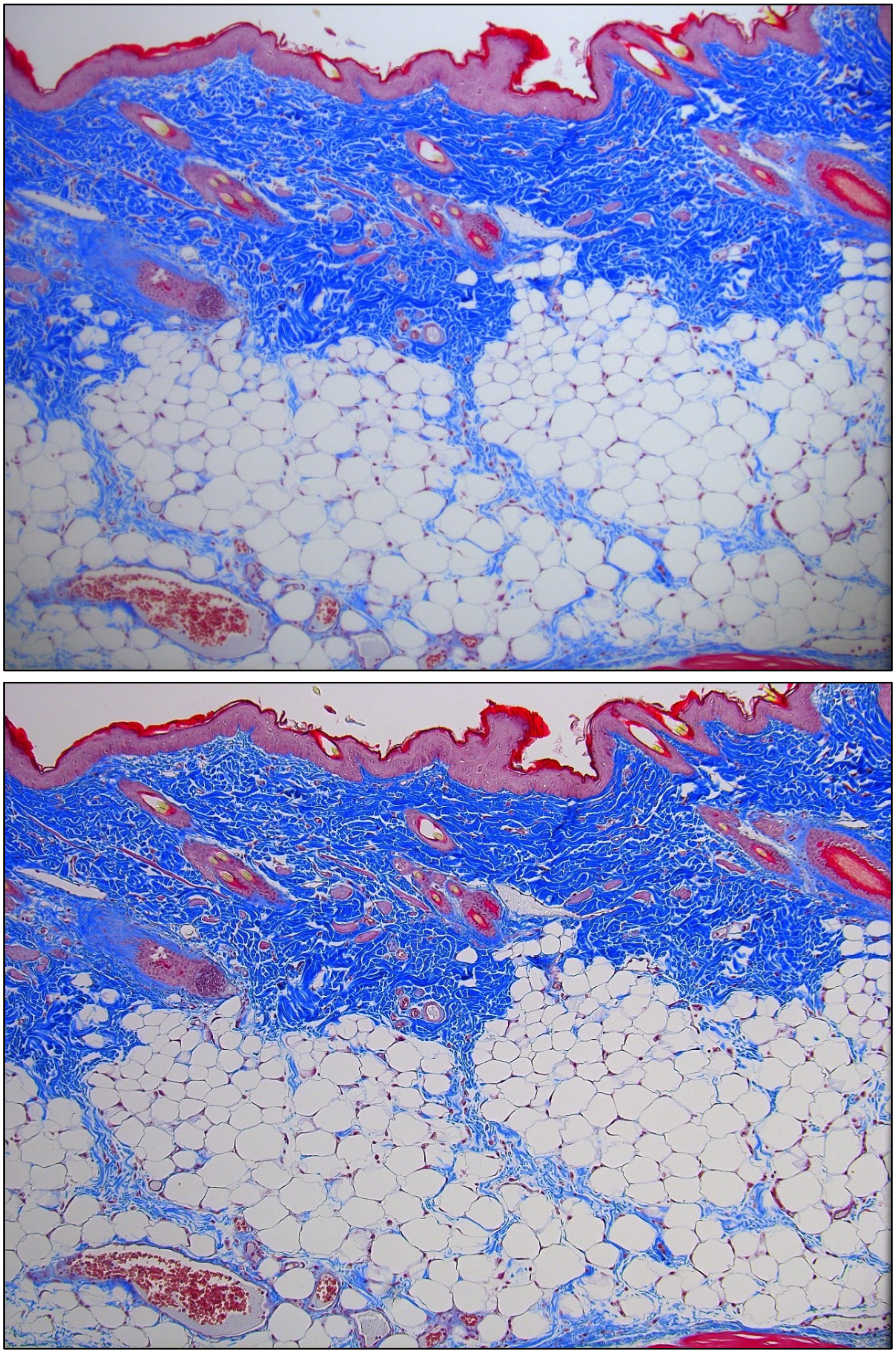

Figure S13. Non-acceptable (upper panel) and acceptable (bottom panel) quality of raw unprocessed micrographs of Masson's Trichrome-stained histological sections of rat's BEFAF. The subpar quality image is too blurry, while the proper one has an adequate focus and contrast. Both micrographs suffer from a minor vignetting artefact mostly pronounced on the lower right side of the image.

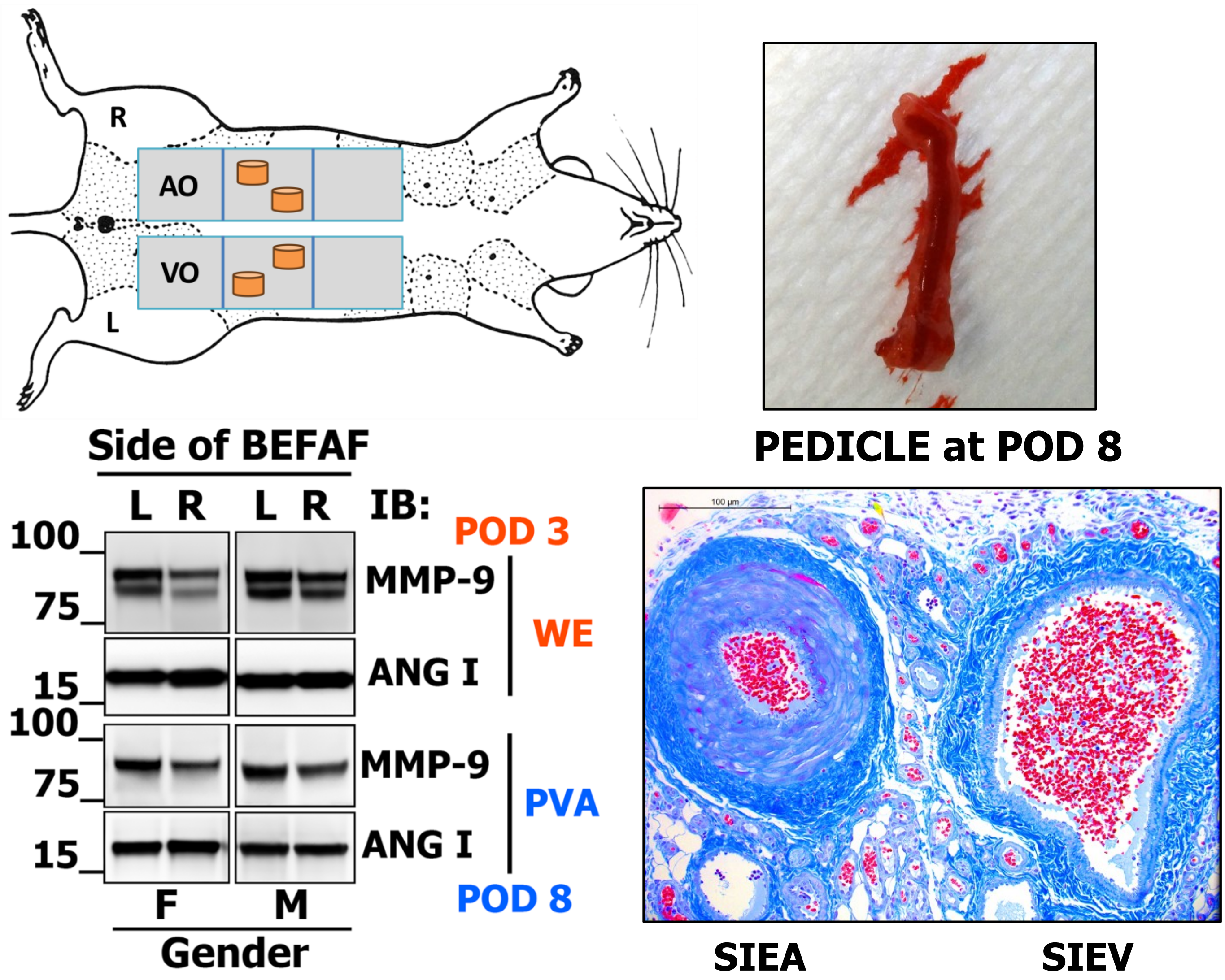

Figure S14. Protein expression in bilateral epigastric fasciocutaneous flap (BEFAF) wound exudate (WE) or PVA sponge fluid. At POD 0, both flaps of adult male (M) or female (F) SD rats were subjected to 2 h global primary ischemia followed by 6 h primary reperfusion. Left-side flap (L) was then subjected to 2 h of secondary ischemia (SI) induced by venous occlusion (VO), whereas right-side flap (R) was subjected to 2 h arterial occlusion (AO). Two sterile PVA sponges were implanted underneath the middle portions of each flap as shown in the **diagram**. At POD 3, WE was collected from the pre-opened flaps. At POD 8, both flaps were excised. The PVA sponges were cleaned from fibrotic tissue and squeezed using tweezers to release the entrapped fluid. An equal volume of 0.45 µm pre-filtered WE or PVA fluid was lysed to extract the proteins. **Left panel** shows immunoblotting (IB) results for the relative MMP-9 (as 92 kDa proenzyme and 84 kDa cleaved active enzyme forms) and Angiogenin expression levels that were detected by using the primary rabbit polyclonal anti-MMP-9 (BioVision, Waltham, MA, #3529) or anti-ANG 1 (C-1) (Santa Cruz Biotechnology, Santa Cruz, CA, # sc-74528) antibodies at 1:500 or 1:1000 dilution, respectively. As a proof of vascular flow recovery post-SI, flap pedicles were dissected, fixed in 4% neutral buffered paraformaldehyde for 48 h and then stained with Masson's Trichrome stain. A representative micrograph of female rat superficial inferior epigastric artery (SIEA) or superficial inferior epigastric vein (SIEV) with entrapped blood cells recorded at 20x magnification is shown in the **right panel**. Erythrocytes are shown in bright red. Collagen is stained in blue.

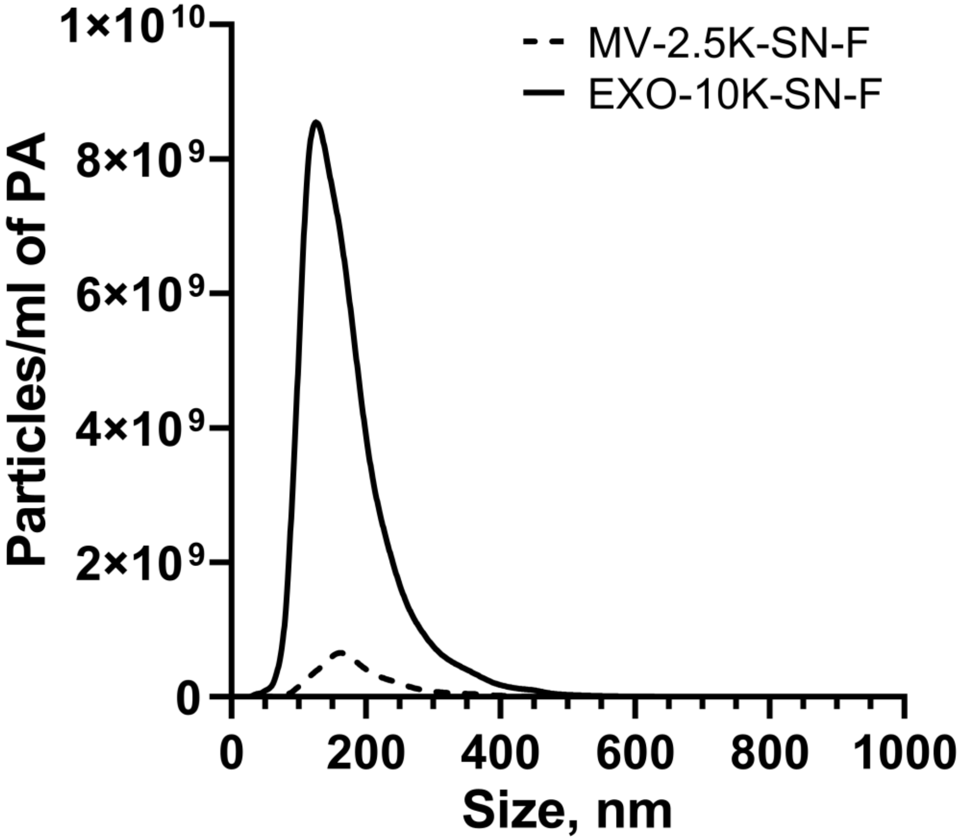

Figure S15. Expected size distribution and concentration of MV-2.5K-SN-F (*dashed line*) and EXO-10K-SN-F (*solid line*) particles derived from 2000 mg of unperturbed control condition abdominal rat skin. Nanoparticle tracking analysis was performed at fixed camera level = 14, detection threshold = 3 and syringe pump speed = 35 settings over 7 repeated measurements with 60 s video capture time in 100-fold or 3200-fold diluted primary analyte (PA) samples using PBS, respectively.

3 . SUPPLEMENTARY REFERENCES

1. Kiyatkin, A. and E. Aksamitiene, *Multistrip western blotting to increase quantitative data output.* Methods Mol Biol, 2009. **536**: p. 149-61.
2. Bosch, S., et al., *Trehalose prevents aggregation of exosomes and cryodamage.* Sci Rep, 2016. **6**: p. 36162.
